# Supplementary material for: Nickel-catalyzed asymmetric hydrogenation for the preparation of α-substituted propionic acids
Source: Nat Commun. 2024 Jun 28;15:5482. doi: 10.1038/s41467-024-49801-0 (PMC11213955; doi:10.1038/s41467-024-49801-0)
Supplement: Supplementary file 1 — Supplementary Information [file 41467_2024_49801_MOESM1_ESM.pdf]

Supplementary information

Nickel-Catalyzed Asymmetric Hydrogenation for Preparation of α-Substituted Propionic Acids

Supplementary Methods

General Details

All hydrogenation reactions were performed in an autoclave under an atmosphere of hydrogen. Solvents were dried and distilled by standard procedures. Commercially available reagents were used without further purification. Nickel salt and ligands were purchased from chemical vendors and used directly without any treatment. α-substituted acrylic acids **1a** and **1t** are commercially available and were used as supplied.

^{1}H NMR, ^{13}C NMR, ^{19}F NMR spectra were recorded on a Bruker AVIII400 instrument with TMS as an inernal standard. Measurements were referenced to the solvent. NMR data is referenced as chemical shift (δ ppm), multiplicity (s = singlet, d = doublet, t = triplet, q = quartet, m = multiplet), coupling constant (Hz), integration. Flash column chromatography was performed using 200 - 300 mesh silica gel. Optical rotations were measured on a Rudolph Research Analytical Autopol VI automatic polarimeter using a 50 mm path-length cell at 589 nm. Enantioselectivity was measured by high performance liquid chromatography (HPLC) using Daicel Chiralcel OJ-H, AD-H and Agilent Eclipse XDB-18 column with hexane/2-propanol or MeCN/water as eluent.

Supplementary Note 1

Synthesis of α-Substituted Acrylic Acids

Procudure A[1]: Preparation of α-substituted acrylic acids 1b-1k, 1m-1s.

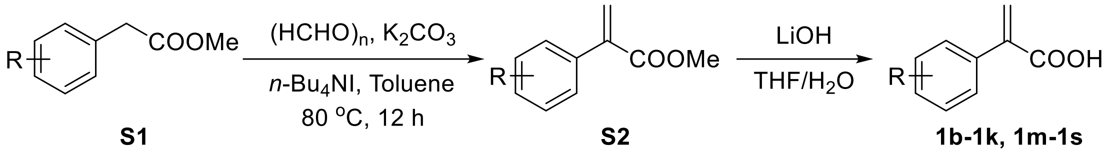

To a toluene solution (40 mL) of ester **S1** (30 mmol, 1.0 equiv.), K_2CO_3 (6.2 g, 45 mmol, 1.5 equiv.), tetrabutylammonium iodide (1.1 g, 3.0 mmol, 0.10 equiv.) and paraformaldehyde (1.4 g, 45 mmol, 1.5 equiv.) were added. The reaction mixture was stirred at 80 °C in an oil bath for 12 h, and monitored by TLC. Upon completion, the reaction was cooled to room temperature, and the resulting mixture was quenched with H_2O and extracted with ethyl acetate (3×30 mL). The combined organic solution was dried over anhydrous Na_2SO_4 and the solvent removed under reduced pressure. The resulting crude product was purified by flash chromatography on silica gel (eluent: PE:EA = 20:1) to give the corresponding *α*-substituted methyl acrylates **S2**.

A 100 mL round-bottom flask was charged with α-substituted methyl acrylate derivative **S2** (10 mmol, 1.0 equiv.), LiOH (1.2 g, 50 mmol, 5.0 equiv.), THF/H_2O (*v*:*v* =1:1, 40 mL) sequentially. The reaction mixture was stirred at 80 °C in an oil bath for 3 h. Upon completion, the reaction was cooled to room temperature. The resulting mixture was extracted with petroleum ether (3×30 mL). The aqueous phase was acidified with aqueous HCl (4.0 M, 12 mL) at 0 °C and extracted with EtOAc (3×30 mL). The combined organic solution was dried over anhydrous Na_2SO_4. The volatile compounds were removed under reduced pressure and recrystallized with EtOAc to afford pure α-substituted acrylic acids **1b-1k**, **1m-1s**.

Procudure B[1]: Preparation of α-substituted acrylic acids 1u-1y.

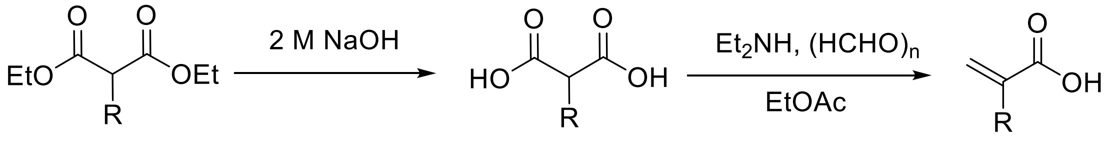

S3 **1u-1y** **1u-1y**

The substituted malonate **S3** (10 mmol, 1.0 equiv.) was added to a round bottle charged with aqueous NaOH (2.0 M, 15 mL), and the resulting mixture was refluxed for 2 h. The resulting solution was cooled to room temperature, and then acidified to pH = 1 with concentrated HCl at 0 °C. The resulting solution was extracted with EtOAc (3×30 mL). The organic phase was washed with brine, dried over anhydrous Na_2SO_4 and concentrated under reduced pressure. The crude product **S4** was used in the next step without further purification.

The diacid **S4** was dissolved in EtOAc (15 mL) and then cooled to 0 °C. Diethylamine (0.80 g, 11 mmol, 1.1 equiv.) was added dropwise to the resulting solution, followed by the addition of paraformaldehyde (0.45 g, 15 mmol, 1.5 equiv.). The resulting suspension was refluxed for 2 h. When the reaction was completed, the mixture was quenched with H_2O, acidified to pH = 1 with concentrated HCl, and extracted with EtOAc (3×20 mL). The combined organic phase was dried over anhydrous Na_2SO_4, and the solvent was removed under reduced pressure. The residue was purified by column chromatography (eluent: PE:EA = 10:1) to afford the pure products **1u-1y**.

Procudure C[2]: Preparation of α-substituted acrylic acids 1l.

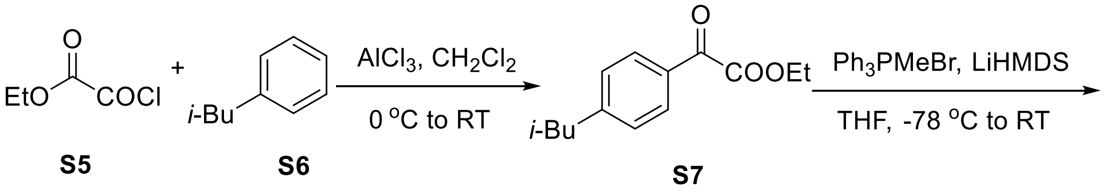

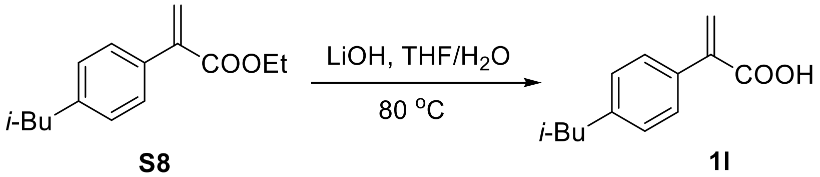

To a dried 250 mL two-neck round-bottom flask charged with aluminum chloride (16.0 g, 120 mmol, 2.40 equiv.) and anhydrous CH_2Cl_2 (300 mL), ethyl oxalyl monochloride **S5** (10.6 mL, 100 mmol, 2.00 equiv.) and isobutylbenzene **S6** (6.71 g, 50.0 mmol, 1.00 equiv.) were added at 0 °C. The reaction mixture was stirred at room temperature for 3 h, and monitored by TLC. Upon completion, the reaction mixture was quenched with crushed ice and aqueous HCl (4.00 M), and the mixture was extracted with CH_2Cl_2 (3×50 mL). The combined organic layers were washed with saturated sodium bicarbonate

solution and brine, dried over anhydrous Na_2SO_4 and evaporated under reduced pressure to afford crude α-ketoesters, which were purified by column chromatography (PE:EA = 20:1) to afford pure α-ketoesters **S7**.

To a dried 250 mL two-neck round-bottom flask, methyltriphenylphsphonium bromide (21.5 g, 60.0 mmol, 1.20 equiv.), dry THF (100 mL), and LiHMDS (50.0 mL, 1.00 M in THF, 50.0 mmol, 1.00 equiv.) were added under a nitrogen atmosphere at -78 ^{o}C. The reaction mixture was stirred at -78 ^{o}C for 20 minutes and then warmed to room temperature, stirring for another 1 h. The mixture was cooled to -78 °C and then α-ketone ester **S7** obtained in the previous step was added. After stirring for 1 h at -78 ^{o}C, the reaction mixture was warmed to room temperature and monitored by TLC. When the reaction was finished, aqueous HCl (4.00 M, 10.0 mL) was added and extracted with ethyl acetate (3×50.0 mL). The combined organic layers were separated, dried over anhydrous Na_2SO_4 and evaporated under reduced pressure. The residue was purified by column chromatography (PE:EA = 20:1) to deliver the α-substituted ethyl acrylate derivatives **S8**. The next procedures were to follow the steps of synthesis of **1b**.

Procudure D[1]: Preparation of α-substituted acrylic acids **1aa**, **1ab**.

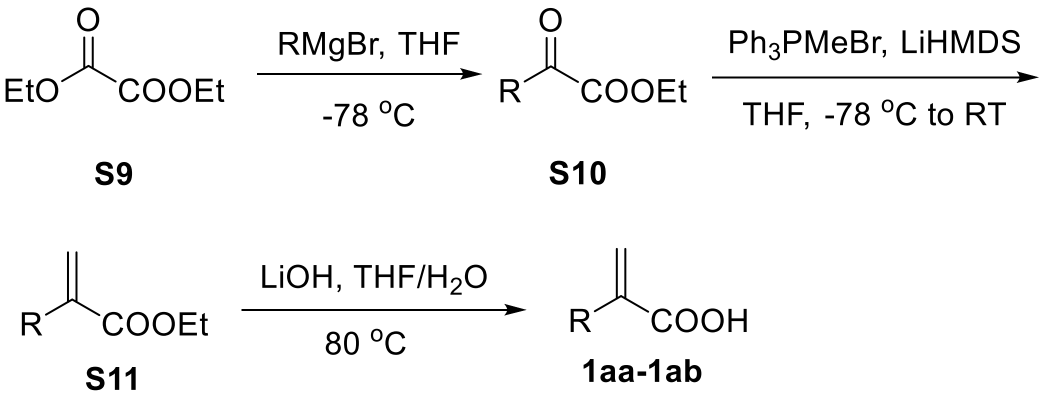

To a THF (40 mL) solution of diethyl oxalate **S9** (2.9 g, 20 mmol, 1.0 equiv.), the fresh Grignard reagents (prepared from corresponding aryl bromide) was added dropwise over 1 h at -78 °C. After stirring for 1 h at -78 °C, the mixture was warmed to room temperature and quenched with NH_4Cl solution. The aqueous layer was extracted with ethyl acetate (3×40 mL) and the combined organic layers were dried over anhydrous Na_2SO_4. The solvent was removed under reduced pressure and the crude ketoesters **S10** were directly used in the

next step without further purification. The next procedures were to follow the steps of synthesis of **1l**.

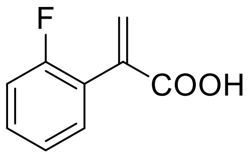

2-(2-Fluorophenyl)acrylic acid (1b)[3]

Following procedure A, white solid (2.09 g, 42%); **^{1}H NMR** (400 MHz, Chloroform-*d*) *δ* 10.66 (br, 1H), 7.27 – 7.18 (m, 2H), 7.09 – 6.91 (m, 2H), 6.55 (s, 1H), 5.90 (s, 1H).

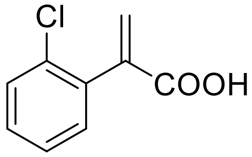

2-(2-Chlorophenyl)acrylic acid (1c)[4]

Following procedure A, white solid (3.44 g, 63%); **^{1}H NMR** (400 MHz, Chloroform-*d*) *δ* 7.50 – 7.44 (m, 1H), 7.40 – 7.30 (m, 3H), 6.40 (d, *J* = 0.8 Hz, 1H), 5.83 (d, *J* = 0.8 Hz, 1H).

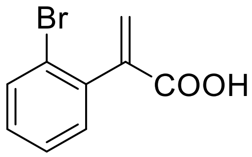

2-(2-Bromophenyl)acrylic acid (1d)[5]

Following procedure A, white solid (3.20 g, 47%); **^{1}H NMR** (400 MHz, DMSO-d_6) *δ* 12.68 (br, 1H), 7.63 (d, *J* = 7.9, 1H), 7.45 – 7.35 (m, 1H), 7.34 – 7.24 (m, 2H), 6.40 (s, 1H), 5.79 (s, 1H).

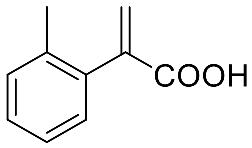

2-(*o*-Tolyl)acrylic acid (1e)[6]

Following procedure A, white solid (2.40 g, 46%); **^{1}H NMR** (400 MHz, Chloroform-*d*): *δ* 11.82 (br, 1H), 7.30 – 7.12 (m, 4H), 6.64 (d, *J* = 1.5 Hz, 1H), 5.83 (d, *J* = 1.5 Hz, 1H), 2.23 (s, 3H).

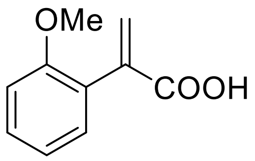

2-(2-Methoxyphenyl)acrylic acid (1f)[5]

Following procedure A, white solid (3.10 g, 58%); **^{1}H NMR** (400 MHz, Chloroform-*d*) δ 9.61 (br, 1H), 7.27 – 7.23 (m, 1H), 7.19 – 7.10 (m, 1H), 6.91 – 6.86 (m, 1H), 6.82 (d, *J* = 8.3, 1H), 6.36 (s, 1H), 5.78 (s, 1H), 3.72 (s, 3H).

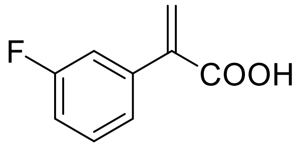

2-(3-Fluorophenyl)acrylic acid (1g)[4]

Following procedure A, white solid (2.64 g, 53%); **^{1}H NMR** (400 MHz, DMSO-*d*6) δ 7.45 – 7.37 (m, 1H), 7.33 – 7.25 (m, 2H), 7.21 – 7.15 (m, 1H), 6.30 (d, *J* = 0.8 Hz, 1H), 6.06 (d, *J* = 0.8 Hz, 1H).

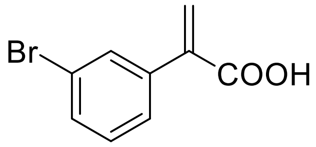

2-(3-Bromophenyl)acrylic acid (1h)[4]

Following procedure A, white solid (3.90 g, 57%); **^{1}H NMR** (400 MHz, Chloroform-*d*) δ 7.63 – 7.62 (m, 1H), 7.54 – 7.50 (m, 1H), 7.40 – 7.38 (m, 1H), 7.26 – 7.25 (m, 1H), 6.60 (s, 1H), 6.07 (s, 1H).

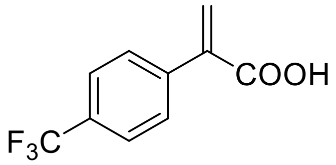

2-(4-(Trifluoromethyl)phenyl)acrylic acid (1i)[1]

Following procedure A, white solid (2.72 g, 42%); **^{1}H NMR** (400 MHz, Chloroform-*d*) δ 7.64 (d, *J* = 8.0 Hz, 2H), 7.56 (d, *J* = 8.1 Hz, 2H), 6.66 (s, 1H), 6.11 (s, 1H).

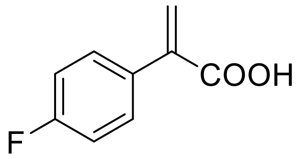

2-(4-Fluorophenyl)acrylic acid (1j)[1]

Following procedure A, white solid (3.03 g, 61%); **^{1}H NMR** (400 MHz, DMSO-d_6) δ 12.84 (br, 1H), 7.53 – 7.43 (m, 2H), 7.24 – 7.14 (m, 2H), 6.25 (d, J = 1.1 Hz, 1H), 5.97 (d, J = 1.1 Hz, 1H).

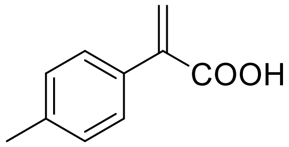

2-(*p*-Tolyl)acrylic acid (1k)[1]

Following procedure A, white solid (2.82 g, 58%); **^{1}H NMR** (400 MHz, Chloroform-*d*): δ 12.24 (br, 1H), 7.32 (d, J = 7.1 Hz, 2H), 7.19 (d, J = 7.3 Hz, 2H), 6.55 (s, 1H), 6.01 (s, 1H), 2.38 (s, 3H).

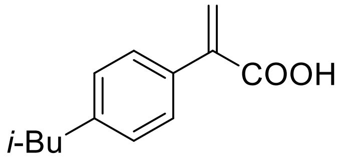

2-(4-Isobutylphenyl)acrylic acid (1l)[7]

Following procedure C, white solid (5.20 g, 51%); **^{1}H NMR** (400 MHz, Chloroform-*d*) δ 7.35 (d, J = 7.8 Hz, 2H), 7.13 (d, J = 7.8 Hz, 2H), 6.48 (s, 1H), 5.99 (s, 1H), 2.47 (d, J = 7.2 Hz, 2H), 1.91 – 1.81 (m, 1H), 0.91 (d, J = 6.6 Hz, 6H).

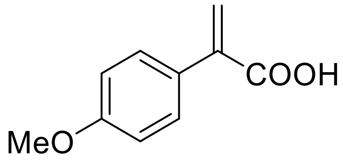

2-(4-Methoxyphenyl)acrylic acid (1m)[1]

Following procedure A, white solid (3.47 g, 65%); **^{1}H NMR** (400 MHz, Chloroform-*d*) δ 7.45 – 7.39 (m, 2H), 6.94 – 6.88 (m, 2H), 6.45 (d, J = 0.8 Hz, 1H), 5.98 (d, J = 0.8 Hz, 1H), 3.83 (s, 3H).

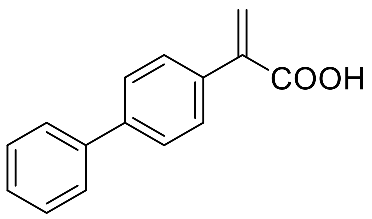

2-([1,1'-Biphenyl]-4-yl)acrylic acid (1n)[1]

Following procedure A, white solid (3.90 g, 58%); **^{1}H NMR** (400 MHz, DMSO-*d_6*): *δ* 7.72 – 7.62 (m, 4 H), 7.52 (d, *J* = 8.1 Hz, 2 H), 7.44 (t, *J* = 7.6 Hz, 2 H), 7.35 (t, *J* = 7.5 Hz, 1 H), 6.56 (s, 1 H), 6.02 (s, 1 H).

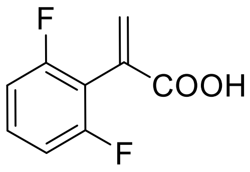

2-(2,6-Difluorophenyl)acrylic acid (1o)

Following procedure A, white solid (2.59 g, 47%); **^{1}H NMR** (400 MHz, Chloroform-*d*) *δ* 7.35 – 7.26 (m, 1H), 6.99 – 6.88 (m, 2H), 6.86 (s, 1H), 6.08 (s, 1H); **^{13}C NMR** (101 MHz, Chloroform-*d*) *δ* 170.95, 160.48 (dd, *J* = 249.7, 6.8 Hz), 134.84, 130.21 (t, *J* = 10.2 Hz), 129.14, 113.67 (t, *J* = 19.7 Hz), 112.38 – 110.11 (m, 2C); **^{19}F NMR** (376 MHz, Chloroform-*d*): *δ* -75.27; **HRMS (ESI-MS)** Calcd. For $C_9H_6F_2O_2$ $[M+Na]^+$ 207.0228, found: 207.0220.

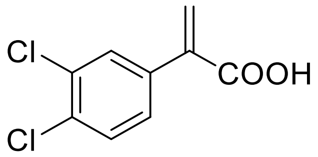

2-(3,4-Dichlorophenyl)acrylic acid (1p)[4]

Following procedure A, white solid (2.47 g, 38%); **^{1}H NMR** (400 MHz, Chloroform-*d*) *δ* 7.56 (d, *J* = 2.1 Hz, 1H), 7.44 (d, *J* = 8.4 Hz, 1H), 7.29 (dd, *J* = 8.3, 2.2 Hz, 1H), 6.58 (d, *J* = 0.8 Hz, 1H), 6.06 (d, *J* = 0.8 Hz, 1H).

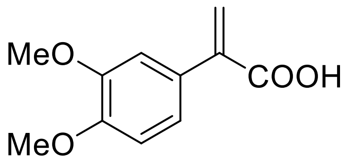

2-(3,4-Dimethoxyphenyl)acrylic acid (1q)

Following procedure A, white solid (2.06 g, 33%); **^{1}H NMR** (400 MHz, Chloroform-*d*) δ 7.06 – 6.99 (m, 2H), 6.87 (d, *J* = 8.3 Hz, 1H), 6.47 (s, 1H), 5.98 (s, 1H), 3.90 (s, 6H); **^{13}C NMR** (101 MHz, Chloroform-*d*) δ 172.41, 149.41, 148.56, 140.24, 128.94, 128.41, 121.23, 111.89, 110.87, 56.04, 56.01; **HRMS (ESI-MS)** Calcd. For $C_{11}H_{12}O_4$ $[M+Na]^+$ 231.0628, found: 231.0624.

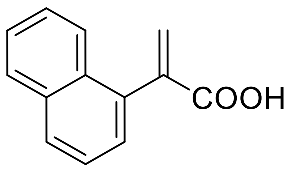

2-(Naphthalen-1-yl)acrylic acid (1r)[4]

Following procedure A, white solid (2.61 g, 44%); **^{1}H NMR** (400 MHz, Chloroform-*d*) δ 7.87 – 7.85 (m, 2H), 7.77 – 7.76 (m, 1H), 7.49 – 7.45 (m, 3H), 7.38 (d, *J* = 6.8 Hz, 1H), 6.82 (d, *J* = 1.2 Hz, 1H), 6.02 (d, *J* = 1.2 Hz, 1H).

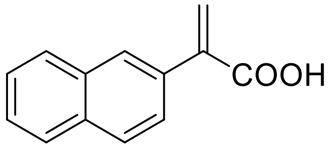

2-(Naphthalen-2-yl)acrylic acid (1s)[4]

Following procedure A, white solid (3.26 g, 55%); **^{1}H NMR** (400 MHz, Chloroform-*d*) δ 7.95 (s, 1H), 7.88 – 7.82 (m, 3H), 7.56 (dd, *J* = 8.4, 1.2 Hz, 1H), 7.52 – 7.47 (m, 2H), 6.63 (s, 1H), 6.15 (s, 1H).

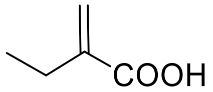

2-Methylenebutanoic acid (1u)[4]

Following procedure B, colorless oil (0.87 g, 87%); **^{1}H NMR** (400 MHz, Chloroform-*d*) δ 11.61 (br, 1H), 6.29 (d, *J* = 0.8 Hz, 1H), 5.65 (d, *J* = 1.2 Hz, 1H), 2.33 (q, *J* = 7.2 Hz, 2H), 1.10 (t, *J* = 7.2 Hz, 3H).

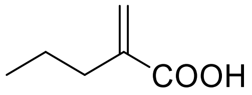

2-Methylenepentanoic acid (1v)[4]

Following procedure B, colorless oil (0.91 g, 80%); **^{1}H NMR** (400 MHz, Chloroform-*d*) *δ* 11.95 (br, 1H), 6.31 (d, *J* = 0.4 Hz, 1H), 5.65 (d, *J* = 1.2 Hz, 1H), 2.27 (t, *J* = 8.0 Hz, 2H), 1.56 – 1.47 (m, 2H), 0.92 (t, *J* = 7.2 Hz, 3H).

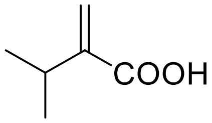

2-Methyl-2-methylenebutanoic acid (1w)[4]

Following procedure B, colorless oil (0.97 g, 85%); **^{1}H NMR** (400 MHz, Chloroform-*d*) *δ* 12.14 (br, 1H), 6.32 (s, 1H), 5.65 (s, 1H), 2.83 – 2.73 (m, 1H), 1.09 (d, *J* = 8.0 Hz, 6H).

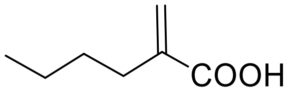

2-Methylenehexanoic acid (1x)[4]

Following procedure B, colorless oil (1.22 g, 95%); **^{1}H NMR** (400 MHz, Chloroform-*d*) *δ* 6.27 (s, 1H), 5.61 (s, 1H), 2.32 (t, *J* = 7.6 Hz, 2H), 1.50-1.41 (m, 2H), 1.38-1.31 (m, 2H), 0.93 (t, *J* = 7.2 Hz, 3H).

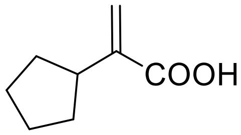

2-Cyclopentylacrylic acid (1y)[4]

Following procedure B, colorless oil (1.29 g, 92%); **^{1}H NMR** (400 MHz, Chloroform-*d*) *δ* 11.96 (br, 1H), 6.26 (s, 1H), 5.64 (s, 1H), 2.88 – 2.78 (m, 1H), 1.97 – 1.88 (m, 2H), 1.74 – 1.57 (m, 4H), 1.45 – 1.36 (m, 2H).

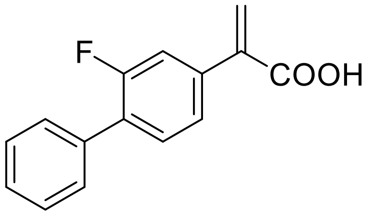

2-(2-Fluoro-[1,1'-biphenyl]-4-yl)acrylic acid (1aa)[8]

Following procedure D, white solid (2.18 g, 45%); **^{1}H NMR** (400 MHz, Chloroform-*d*) δ 7.60 – 7.53 (m, 2H), 7.48 – 7.44 (m, 3H), 7.39 – 7.36 (m, 1H), 7.34 – 7.28 (m, 2H), 6.52 (s, 1H), 6.12 (s, 1H).

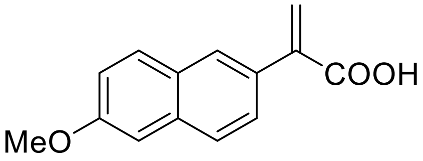

2-(6-Methoxynaphthalen-2-yl)acrylic acid (1ab)[8]

Following procedure D, white solid (1.50 g, 33%); **^{1}H NMR** (400 MHz, Chloroform-*d*) *δ* 7.86 (s, 1H), 7.73 (t, *J* = 8.6 Hz, 2H), 7.52 (d, *J* = 8.4 Hz, 1H), 7.14 (d, *J* = 12.3 Hz, 2H), 6.56 (s, 1H), 6.10 (s, 1H), 3.92 (s, 3H).

Optimization of Reaction Conditions

Supplementary Table 1 Reaction condition screening[a]

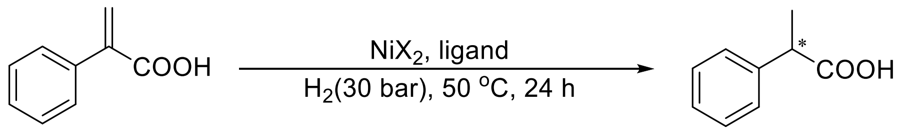

1a **2a**

Entry	Salt/Ligand	Solvent	Conv. (%)[b]	ee (%)[c]
1	(*R*,*R*)-QuinoxP*/$Ni(OAc)_2 \cdot 4H_2O$	TFE	70	76
2	(*R*,*R*)-BenzP*/$Ni(OAc)_2 \cdot 4H_2O$	TFE	>99	96
3	(*R*)-BINAP/$Ni(OAc)_2 \cdot 4H_2O$	TFE	17	-
4	(*S*)-SegPhos/$Ni(OAc)_2 \cdot 4H_2O$	TFE	<5	-
5	(*S*)-DTBM-SegPhos/$Ni(OAc)_2 \cdot 4H_2O$	TFE	<5	-
6	(*R*,*Sp*)-JosiPhos/$Ni(OAc)_2 \cdot 4H_2O$	TFE	<5	-
7	(*S*,*S*)-Ph-BPE/$Ni(OAc)_2 \cdot 4H_2O$	TFE	<5	-
8	(*R*,*R*)-BenzP*/$Ni(OAc)_2 \cdot 4H_2O$	MeOH	7	-
9	(*R*,*R*)-BenzP*/$Ni(OAc)_2 \cdot 4H_2O$	EtOH	8	-
10	(*R*,*R*)-BenzP*/$Ni(OAc)_2 \cdot 4H_2O$	*i*-PrOH	<5	-
11	(*R*,*R*)-BenzP*/$Ni(OAc)_2 \cdot 4H_2O$	THF	<5	-
12	(*R*,*R*)-BenzP*/$Ni(OAc)_2 \cdot 4H_2O$	toluene	<5	-
13	(*R*,*R*)-BenzP*/$Ni(OAc)_2 \cdot 4H_2O$	AcOH	23	95
14	(*R*,*R*)-BenzP*/$Ni(OAc)_2 \cdot 4H_2O$	TFE:MeOH 2:1	19	94
15	(*R*,*R*)-BenzP*/$Ni(OAc)_2 \cdot 4H_2O$	TFE:EtOH 2:1	17	93
16	(*R*,*R*)-BenzP*/$Ni(OAc)_2 \cdot 4H_2O$	TFE:EA 2:1	28	96
17	(*R*,*R*)-BenzP*/$Ni(OAc)_2 \cdot 4H_2O$	TFE:AcOH 2:1	>99	95
18	(*R*,*R*)-BenzP*/$Ni(OAc)_2 \cdot 4H_2O$	TFE:AcOH 1:2	>99	95
19	(*R*,*R*)-BenzP*/$Ni(OAc)_2 \cdot 4H_2O$	TFE:AcOH 1:10	81	95
20	(*R*,*R*)-BenzP*/$Ni(OAc)_2 \cdot 4H_2O$	TFE:AcOH 1:20	57	95
21	(*R*,*R*)-BenzP*/$Ni(OAc)_2$	TFE	>99	96
22	(*R*,*R*)-BenzP*/$Ni(BF_4)_2 \cdot 6H_2O$	TFE	>99	96
23	(*R*,*R*)-BenzP*/$Ni(CF_3SO_3)_2$	TFE	>99	96
24	(*R*,*R*)-BenzP*/$Ni(ClO_4)_2 \cdot 6H_2O$	TFE	65	96
25	(*R*,*R*)-BenzP*/$NiCl_2$	TFE	-	-

26[d]	(*R,R*)-BenzP*/Ni(OAc)$_2$·4H$_2$O	TFE	93	98
27[d,e]	(*R,R*)-BenzP*/Ni(OAc)$_2$·4H$_2$O	TFE	97	97
28[f]	(*R,R*)-BenzP*/Ni(OAc)$_2$·4H$_2$O	TFE	>99	96

[a]Reaction condition: **1a** (0.2 mmol), Ni salt (1 mol% S/C = 100), ligand (1 mol%), solvent (1.0 mL), H_2 (30 bar), 50 °C, 24 h. [b]The conversions were calculated from ^{1}H NMR spectra. [c]The ee values were determined by HPLC using chiral columns. [d]30 °C. [e]50 bar H_2. [f]**1a** (0.5 mmol), (*R,R*)-BenzP*/Ni(OAc)$_2$·4H$_2$O (0.2 mol% S/C = 500), TFE (2.0 mL), H_2 (30 bar), 50 °C, 24 h.

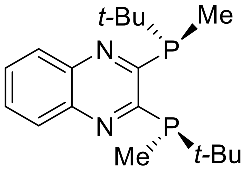

(*R*,*R*)-QuinoxP*

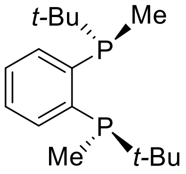

(*R*,*R*)-BenzP*

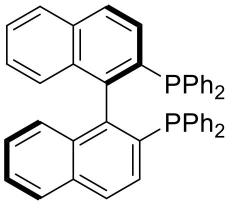

(*R*)-BINAP

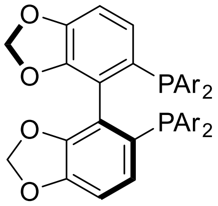

(*S*)-Segphos, Ar = Ph
(*S*)-DTBM-Segphos,
Ar = 3,5-di*t*-Bu-4-MeO-C_6H_2

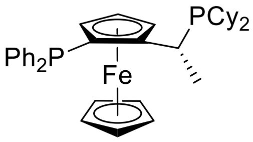

(*R*,*Sp*)-JosiPhos

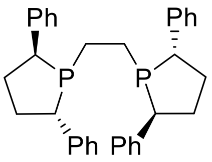

(*S*,*S*)-Ph-BPE

Asymmetric Hydrogenation of α-Substituted Acrylic Acids

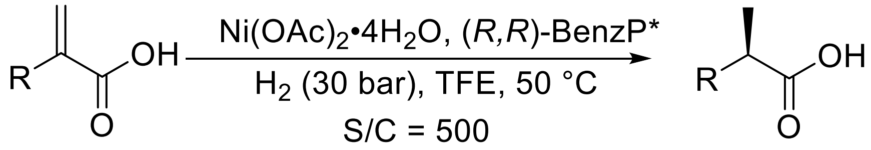

Procedure E: (*R, R*)-BenzP* (1.96 mg, 0.20 mol%×7) and $Ni(OAc)_2 \cdot 4H_2O$ (1.75 mg, 0.20 mol%×7) were placed in a dried Schlenk tube under nitrogen atmosphere, and degassed anhydrous trifluoroethanol (TFE, 7.0 mL) was added. The mixture was stirred at room temperature for 20 min. In a glovebox, the solution was evenly divided into seven dried Schlenk tubes containing seven different substrates (S/C = 500) in a stainless steel autoclave. Then 1.0 mL TFE was added to each tube. The reaction was stirred under H_2 (30 bar) at 50 ^{o}C in a stainless steel autoclave for 24 h. After carefully releasing the H_2 gas, the solution was concentrated under reduced pressure and purified by column chromatography (DCM:MeOH = 8:1) to give the desired product. The product was reacted with K_2CO_3/Me_2SO_4 or DMAP/DCC/aniline (**2u-2y**) to afford the corresponding methyl ester or amide, whose ee value was determined by HPLC with a chiral column.

Procedure F: $Ni(OAc)_2 \cdot 4H_2O$ (0.50 mg, 1.0 mol%), (*R,R*)-BenzP* (0.56 mg, 1.0 mol%) and substrate (S/C = 100) were placed in a dried Schlenk tube. The tube was then transferred to a nitrogen-filled glovebox and degassed anhydrous trifluoroethanol (TFE, 2.0 mL) was added. The remaining procedures followed the above **Procedure E**.

Procedure G: Following the above **Procedure F**, S/C = 250.

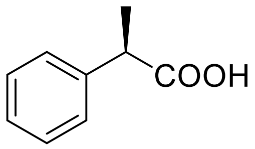

(*R*)-2-Phenylpropanoic acid (2a)[9]

Following procedure E, colorless oil (73.6 mg, 98% yield, 96% ee); $[\alpha]_D^{20}$ = –63.1 (*c* = 0.59, CH_2Cl_2); **^{1}H NMR** (400 MHz, Chloroform-*d*): *δ* 7.33 – 7.06 (m, 5H), 3.64 (q, *J* = 7.2 Hz, 1H), 1.42 (d, *J* = 7.2 Hz, 3H); **^{13}C NMR** (101 MHz, Chloroform-*d*): *δ* 181.23, 139.85, 128.81, 127.74, 127.53, 45.53, 18.20; HPLC

conditions (***S*-2a**): DAICEL Chiralpak OJ-H column, Hexane/*i*PrOH = 99/1, 210 nm, 1.0 mL/min, 25 ^{o}C, t_{major} = 13.178 min, t_{major} = 16.492 min.

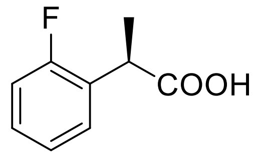

(*R*)-2-(2-Fluorophenyl)propanoic acid (2b)[9]

Following procedure E, colorless oil (79.0 mg, 94% yield, 97% ee); $[\alpha]_D^{20}$ = –34.6 (*c* = 0.55, CH_2Cl_2); **^{1}H NMR** (400 MHz, Chloroform-*d*): *δ* 10.68 (br, 1H), 7.24 – 7.20 (m, 1H), 7.19 – 7.10 (m, 1H), 7.04 – 7.00 (m, 1H), 6.98 – 6.93 (m, 1H), 3.97 (q, *J* = 7.2 Hz, 1H), 1.43 (d, *J* = 7.3 Hz, 3H); **^{13}C NMR** (101 MHz, Chloroform-*d*) *δ* 180.48, 160.53 (d, *J* = 246.5 Hz), 129.06 (d, *J* = 8.3 Hz), 128.90 (d, *J* = 3.9 Hz), 127.19 (d, *J* = 14.9 Hz), 124.45 (d, *J* = 3.6 Hz), 115.63 (d, *J* = 22.3 Hz), 38.56, 17.24; **^{19}F NMR** (376 MHz, Chloroform-*d*): *δ* -117.69; HPLC conditions: DAICEL Chiralpak OJ-H column, Hexane/*i*PrOH = 99/1, 210 nm, 1.0 mL/min, 25 ^{o}C, t_{minor} = 9.738 min, t_{major} = 10.930 min.

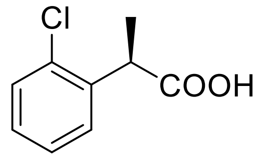

(*R*)-2-(2-Chlorophenyl)propanoic acid (2c)[9]

Following procedure E, colorless oil (87.4 mg, 95% yield, 99.4% ee); $[\alpha]_D^{20}$ = –61.1 (*c* = 0.66, CH_2Cl_2); **^{1}H NMR** (400 MHz, Chloroform-*d*) *δ* 10.96 (br, 1H), 7.39 – 7.33 (m, 2H), 7.29 – 7.18 (m, 2H), 4.27 (q, *J* = 7.2 Hz, 1H), 1.52 (d, *J* = 7.2 Hz, 3H); **^{13}C NMR** (101 MHz, Chloroform-*d*) *δ* 180.64, 137.71, 133.93, 129.78, 128.65, 128.58, 127.30, 42.15, 17.37; HPLC conditions: DAICEL Chiralpak OJ-H column, Hexane/*i*PrOH = 99/1, 210 nm, 1.0 mL/min, 25 ^{o}C, t_{major} = 10.174 min, t_{minor} = 11.196 min.

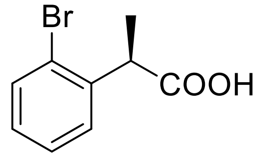

(*R*)-2-(2-Bromophenyl)propanoic acid (2d)[10]

Following procedure G, colorless oil (112.2 mg, 98% yield, 98% ee); $[\alpha]_D^{20}$ = – 64.9 (*c* = 0.59, CH_2Cl_2); **^{1}H NMR** (400 MHz, Chloroform-*d*) *δ* 9.91 (br, 1H), *δ* 7.49 (dd, *J* = 8.0, 1.3 Hz, 1H), 7.31 – 7.19 (m, 2H), 7.08 – 7.01 (m, 1H), 4.21 (q, *J* = 7.3 Hz, 1H), 1.44 (d, *J* = 7.2 Hz, 3H); **^{13}C NMR** (101 MHz, Chloroform-*d*) *δ* 180.22, 139.54, 133.15, 128.93, 128.62, 127.97, 124.68, 44.60, 17.73; HPLC conditions: DAICEL Chiralpak OJ-H column, Hexane/*i*PrOH = 99/1, 210 nm, 1.0 mL/min, 25 ^oC, t_{major} = 11.199 min, t_{minor} = 13.513 min.

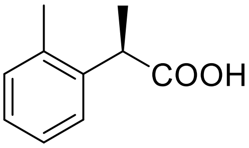

(*R*)-2-(*o*-Tolyl)propanoic acid (2e)[9]

Following procedure E, colorless oil (79.6 mg, 97% yield, 99.1% ee); $[\alpha]_D^{20}$ = – 76.2 (*c* = 0.65, CH_2Cl_2); **^{1}H NMR** (400 MHz, Chloroform-*d*) *δ* 7.33 – 7.25 (m, 1H), 7.22 – 7.10 (m, 3H), 3.97 (q, *J* = 7.1 Hz, 1H), 2.36 (s, 3H), 1.48 (d, *J* = 7.1 Hz, 3H); **^{13}C NMR** (101 MHz, Chloroform-*d*) *δ* 181.44, 138.45, 136.04, 130.66, 127.32, 126.69, 126.58, 41.30, 19.75, 17.64; HPLC conditions: DAICEL Chiralpak OJ-H column, Hexane/*i*PrOH = 99/1, 210 nm, 1.0 mL/min, 25 ^oC, t_{major} = 8.829 min, t_{minor} = 9.778 min.

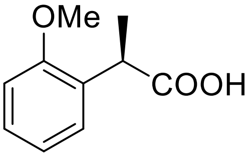

(*R*)-2-(2-Methoxyphenyl)propanoic acid (2f)[9]

Following procedure E, white solid (89.2 mg, 99% yield, 98% ee); $[\alpha]_D^{20}$ = – 75.7 (*c* = 0.82, CH_2Cl_2); **^{1}H NMR** (400 MHz, Chloroform-*d*) *δ* 7.16 – 7.12 (m, 2H), 6.84 (t, *J* = 7.6 Hz, 1H), 6.77 (d, *J* = 8.5 Hz, 1H), 3.99 (q, *J* = 7.2 Hz, 1H), 3.70 (s, 3H), 1.38 (d, *J* = 7.2 Hz, 3H); **^{13}C NMR** (101 MHz, Chloroform-*d*) *δ* 181.57, 156.81, 128.86, 128.44, 128.11, 120.86, 110.84, 55.54, 39.25, 16.97; HPLC

conditions: DAICEL Chiralpak OJ-H column, Hexane/*i*PrOH = 99/1, 210 nm, 1.0 mL/min, 25 ^{o}C, t_{minor} = 13.681 min, t_{major} = 16.288 min.

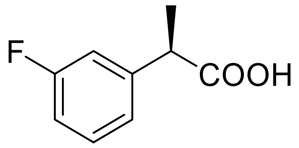

(*R*)-2-(3-Fluorophenyl)propanoic acid (2g)[9]

Following procedure E, colorless oil (83.3 mg, 99% yield, 96% ee); $[\alpha]_D^{20}$ = –61.9 (*c* = 0.66, CH_2Cl_2); **^{1}H NMR** (400 MHz, Chloroform-*d*) *δ* 7.24 – 7.15 (m, 1H), 7.04 – 6.93 (m, 2H), 6.88 (td, *J* = 8.4, 2.5 Hz, 1H), 3.66 (q, *J* = 7.3 Hz, 1H), 1.43 (d, *J* = 7.1 Hz, 3H); **^{13}C NMR** (101 MHz, Chloroform-*d*) *δ* 180.39, 163.04 (d, *J* = 246.2 Hz), 142.18 (d, *J* = 7.3 Hz), 130.25 (d, *J* = 8.2 Hz), 123.54 (d, *J* = 2.8 Hz), 114.82 (d, *J* = 21.8 Hz), 114.51 (d, *J* = 21.1 Hz), 45.26, 18.16; **^{19}F NMR** (376 MHz, Chloroform-*d*): *δ* -112.69; HPLC conditions: DAICEL Chiralpak OJ-H column, Hexane/*i*PrOH = 99/1, 210 nm, 1.0 mL/min, 25 ^{o}C, t_{minor} = 8.570 min, t_{major} = 9.185 min.

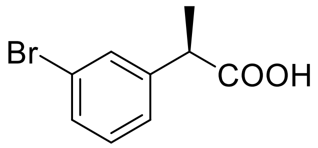

(*R*)-2-(3-Bromophenyl)propanoic acid (2h)[11]

Following procedure F, colorless oil (44.4 mg, 97% yield, 96% ee); $[\alpha]_D^{20}$ = –36.3 (*c* = 0.49, CH_2Cl_2); **^{1}H NMR** (400 MHz, Chloroform-*d*) *δ* 7.39 (s, 1H), 7.34 – 7.32 (d, *J* = 7.8 Hz, 1H), 7.21 – 7.08 (m, 2H), 3.63 (q, *J* = 7.2 Hz, 1H), 1.43 (d, *J* = 7.2 Hz, 3H); **^{13}C NMR** (101 MHz, Chloroform-*d*) *δ* 180.35, 141.95, 130.91, 130.73, 130.35, 126.51, 122.82, 45.19, 18.16; HPLC conditions: DAICEL Chiralpak OJ-H column, Hexane/*i*PrOH = 99/1, 210 nm, 1.0 mL/min, 25 ^{o}C, t_{minor} = 9.255 min, t_{major} = 9.711 min.

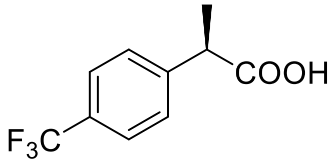

(*R*)-2-(4-(Trifluoromethyl)phenyl)propanoic acid (2i)[9]

Following procedure E, white solid (108.0 mg, 99% yield, 96% ee); $[\alpha]_D^{20}$ = –35.7 (*c* = 0.95, CH_2Cl_2); **^{1}H NMR** (400 MHz, Chloroform-*d*) *δ* 11.50 (br, 1H), 7.58 (d, *J* = 8.0 Hz, 2H), 7.43 (d, *J* = 8.1 Hz, 2H), 3.81 (q, *J* = 7.2 Hz, 1H), 1.53 (d, *J* = 7.2 Hz, 3H); **^{13}C NMR** (101 MHz, Chloroform-*d*) *δ* 180.62, 143.69, 129.90 (q, *J* = 32.4 Hz), 128.24, 125.78 (q, *J* = 3.8 Hz), 124.20 (q, *J* = 272.7 Hz), 45.43, 18.11; **^{19}F NMR** (376 MHz, Chloroform-*d*): *δ* -62.61; HPLC conditions: DAICEL Chiralpak OJ-H column, Hexane/*i*PrOH = 99/1, 210 nm, 1.0 mL/min, 25 ^{o}C, t_{major} = 6.025 min, t_{minor} = 7.259 min.

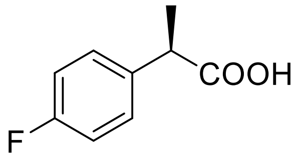

(*R*)-2-(4-Fluorophenyl)propanoic acid (2j)[9]

Following procedure E, colorless oil (81.6 mg, 97% yield, 95% ee); $[\alpha]_D^{20}$ = –50.7 (*c* = 0.54, CH_2Cl_2); **^{1}H NMR** (400 MHz, Chloroform-*d*): *δ* 11.41 (br, 1H), 7.33 – 7.24 (m, 2H), 7.04 – 6.94 (m, 2H), 3.71 (q, *J* = 7.2 Hz, 1H), 1.49 (d, *J* = 7.2 Hz, 3H); **^{13}C NMR** (101 MHz, Chloroform-*d*) *δ* 181.14, 162.24 (d, *J* = 245.8 Hz), 135.52 (d, *J* = 3.3 Hz), 129.32 (d, *J* = 8.0 Hz), 115.64 (d, *J* = 21.4 Hz), 44.78, 18.29; **^{19}F NMR** (376 MHz, Chloroform-*d*): *δ* -115.18; HPLC conditions: DAICEL Chiralpak OJ-H column, Hexane/*i*PrOH = 99/1, 210 nm, 1.0 mL/min, 25 ^{o}C, t_{major} = 10.727 min, t_{minor} = 11.900 min.

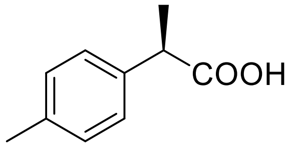

(*R*)-2-(*p*-Tolyl)propanoic acid (2k)[9]

Following procedure E, white solid (78.0 mg, 95% yield, 96% ee); $[\alpha]_D^{20}$ = –66.14 (*c* = 0.68, CH_2Cl_2); **^{1}H NMR** (400 MHz, Chloroform-*d*) *δ* 11.43 (br, 1H), 7.19 (d, *J* = 8.2 Hz, 2H), 7.11 (d, *J* = 7.9 Hz, 2H), 3.68 (q, *J* = 7.2 Hz, 1H), 2.31 (s, 3H), 1.47 (d, *J* = 7.2 Hz, 3H); **^{13}C NMR** (101 MHz, Chloroform-*d*) *δ* 181.41, 137.15, 136.93, 129.47, 127.58, 45.13, 21.14, 18.20; HPLC conditions:

DAICEL Chiralpak OJ-H column, Hexane/*i*PrOH = 99/1, 210 nm, 1.0 mL/min, 25 °C, t_{major} = 13.731 min, t_{minor} = 16.157 min.

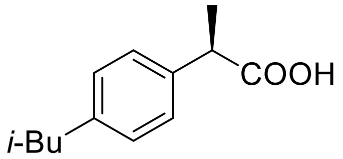

(*R*)-2-(4-Isobutylphenyl)propanoic acid (2l)[9]

Following procedure E, colorless oil (100.1 mg, 97% yield, 96% ee); $[\alpha]_D^{20}$ = – 48.9 (*c* = 1.00, CH_2Cl_2); **^{1}H NMR** (400 MHz, Chloroform-*d*) *δ* 11.53 (br, 1H), 7.21 (d, *J* = 8.0 Hz, 2H), 7.10 (d, *J* = 6.6 Hz, 2H), 3.69 (q, *J* = 7.1 Hz, 1H), 2.44 (d, *J* = 7.2 Hz, 2H), 1.90 – 1.80 (m, 1H), 1.48 (d, *J* = 7.2 Hz, 3H), 0.89 (d, *J* = 6.6 Hz, 6H); **^{13}C NMR** (101 MHz, Chloroform-*d*) *δ* 181.47, 140.95, 137.11, 129.51, 127.42, 45.18, 30.29, 22.52, 18.21; HPLC conditions (***S*-2l**): DAICEL Chiralpak OJ-H column, Hexane/*i*PrOH = 99/1, 210 nm, 1.0 mL/min, 25 °C, t_{major} = 8.333 min, t_{minor} = 9.236 min.

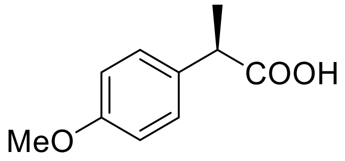

(*R*)-2-(4-Methoxyphenyl)propanoic acid (2m)[9]

Following procedure E, white solid (87.4 mg, 97% yield, 96% ee); $[\alpha]_D^{20}$ = – 41.5 (*c* = 0.82, CH_2Cl_2); **^{1}H NMR** (400 MHz, Chloroform-*d*) *δ* 11.22 (br, 1H), 7.14 (d, *J* = 8.7 Hz, 2H), 6.76 (d, *J* = 8.6 Hz, 2H), 3.68 (s, 3H), 3.59 (q, *J* = 7.2 Hz, 1H), 1.39 (d, *J* = 7.3 Hz, 3H); **^{13}C NMR** (101 MHz, Chloroform-*d*) *δ* 180.24, 157.81, 130.83, 127.59, 113.02, 54.20, 43.50, 17.09; HPLC conditions: DAICEL Chiralpak OJ-H column, Hexane/*i*PrOH = 99/1, 210 nm, 1.0 mL/min, 25 °C, t_{minor} = 26.306 min, t_{major} = 28.620 min.

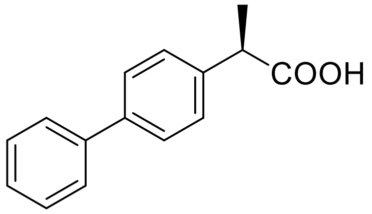

(*R*)-2-([1,1'-Biphenyl]-4-yl)propanoic acid (2n)[9]

Following procedure F, white solid (44.4 mg, 98% yield, 92% ee); $[\alpha]_D^{20}$ = – 13.6 (*c* = 0.16, CH_2Cl_2); **^{1}H NMR** (400 MHz, Chloroform-*d*) *δ* 7.61 – 7.58 (m, 4H), 7.51 – 7.34 (m, 5H), 3.83 (q, *J* = 7.3 Hz, 1H), 1.59 (d, *J* = 7.1 Hz, 3H); **^{13}C NMR** (101 MHz, Chloroform-*d*) *δ* 180.82, 140.83, 140.52, 138.93, 128.88, 128.19, 127.55, 127.43, 127.21, 45.25, 18.27; HPLC conditions: DAICEL Chiralpak OJ-H column, Hexane/*i*PrOH = 99/1, 210 nm, 1.0 mL/min, 25 ^oC, t_{major} = 26.412 min, t_{minor} = 34.517 min.

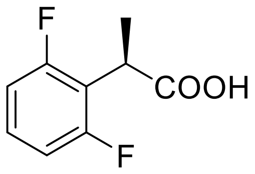

(*R*)-2-(2,6-Difluorophenyl)propanoic acid (2o)[12]

Following procedure E, white solid (91.2 mg, 98% yield, 99.2% ee); $[\alpha]_D^{20}$ = – 38.4 (*c* = 0.75, CH_2Cl_2); **^{1}H NMR** (400 MHz, Chloroform-*d*) *δ* 11.39 (br, 1H), 7.25 – 7.15 (m, 1H), 6.92 – 6.82 (m, 2H), 4.14 (q, *J* = 7.3 Hz, 1H), 1.52 (d, *J* = 7.3 Hz, 3H); **^{13}C NMR** (101 MHz, Chloroform-*d*) *δ* 179.65, 161.04 (dd, *J* = 248.1, 8.1 Hz), 128.95 (t, *J* = 18.2 Hz), 116.86 (t, *J* = 18.7 Hz), 111.69 – 111.14 (m, 2C), 34.60, 16.09； **^{19}F NMR** (376 MHz, Chloroform-*d*): *δ* -114.43; HPLC conditions: DAICEL Chiralpak OJ-H column, Hexane/*i*PrOH = 99/1, 210 nm, 1.0 mL/min, 25 ^oC, t_{major} = 8.233 min, t_{minor} = 8.809 min.

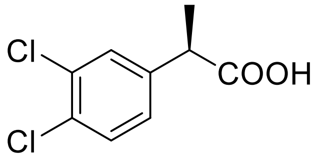

(*R*)-2-(3,4-Dichlorophenyl)propanoic acid (2p)[13]

Following procedure G, white solid (108.4 mg, 99% yield, 95% ee); $[\alpha]_D^{20}$ = –53.1 (*c* = 0.78, CH_2Cl_2); **^{1}H NMR** (400 MHz, Chloroform-*d*) *δ* 10.34 (br, 1H), 7.36 – 7.28 (m, 2H), 7.08 (dd, *J* = 8.3, 2.1 Hz, 1H), 3.62 (q, *J* = 7.2 Hz, 1H), 1.43 (d, *J* = 7.2 Hz, 3H); **^{13}C NMR** (101 MHz, Chloroform-*d*) *δ* 180.13, 139.78, 132.83, 131.77, 130.72, 129.86, 127.26, 44.71, 18.07; HPLC conditions: DAICEL Chiralpak OJ-H column, Hexane/*i*PrOH = 99/1, 210 nm, 1.0 mL/min, 25 °C, t_{major} = 9.207 min, t_{minor} = 9.902 min.

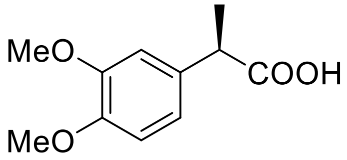

(*R*)-2-(3,4-Dimethoxyphenyl)propanoic acid (2q)[14]

Following procedure E, colorless oil (104.0 mg, 99% yield, 95% ee); $[\alpha]_D^{20}$ = –21.7 (*c* = 1.00, CH_2Cl_2); **^{1}H NMR** (400 MHz, Chloroform-*d*) *δ* 10.96 (br, 1H), 6.87 – 6.76 (m, 3H), 3.84 (s, 3H), 3.82 (s, 3H), 3.65 (q, *J* = 7.2 Hz, 1H), 1.47 (d, *J* = 7.1 Hz, 3H); **^{13}C NMR** (101 MHz, Chloroform-*d*) *δ* 180.98, 148.89, 148.26, 132.21, 119.66, 111.13, 110.70, 55.82, 44.89, 18.12; HPLC conditions: DAICEL Chiralpak OJ-H column, Hexane/*i*PrOH = 80/20, 210 nm, 1.0 mL/min, 25 °C, t_{major} = 12.119 min, t_{minor} = 14.275 min.

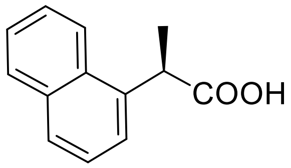

(*R*)-2-(Naphthalen-1-yl)propanoic acid (2r)[9]

Following procedure G, colorless oil (99.0 mg, 99% yield, 99% ee); $[\alpha]_D^{20}$ = –41.5 (*c* = 0.69, CH_2Cl_2); **^{1}H NMR** (400 MHz, Chloroform-*d*) *δ* 9.25 (br, 1H), 7.98 (d, *J* = 8.3 Hz, 1H), 7.76 (d, *J* = 8.0 Hz, 1H), 7.67 (d, *J* = 7.9 Hz, 1H), 7.46 – 7.31 (m, 4H), 4.42 (q, *J* = 7.1 Hz, 1H), 1.56 (d, *J* = 7.1 Hz, 3H); **^{13}C NMR** (101 MHz, Chloroform-*d*) *δ* 181.21, 136.12, 134.06, 131.47, 129.11, 128.12, 126.56, 125.81, 125.66, 124.72, 123.20, 41.24, 17.91; HPLC conditions: DAICEL

Chiralpak OJ-H column, Hexane/*i*PrOH = 99/1, 210 nm, 1.0 mL/min, 25 ^{o}C, t_{minor} = 17.880 min, t_{major} = 20.106 min.

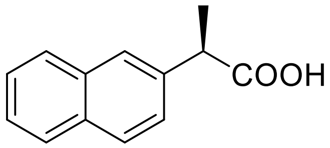

(*R*)-2-(Naphthalen-2-yl)propanoic acid (2s)[3]

Following procedure E, white solid (99.0 mg, 99% yield, 95% ee); $[\alpha]_D^{20}$ = – 40.1 (*c* = 0.70, CH_2Cl_2); **^{1}H NMR** (400 MHz, Chloroform-*d*) δ 10.55 (br, 1H), 7.91 – 7.62 (m, 4H), 7.44 – 7.39 (m, 3H), 3.89 (q, *J* = 7.2 Hz, 1H), 1.58 (d, *J* = 7.3 Hz, 3H); **^{13}C NMR** (101 MHz, Chloroform-*d*) δ 181.04, 137.28, 133.54, 132.81, 128.54, 127.95, 127.75, 126.49, 126.34, 126.05, 125.82, 45.64, 18.23; HPLC conditions: DAICEL Chiralpak OJ-H column, Hexane/*i*PrOH = 99/1, 210 nm, 1.0 mL/min, 25 ^{o}C, t_{minor} = 23.405 min, t_{major} = 25.048 min.

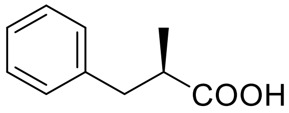

(*R*)-2-Methyl-3-phenylpropanoic acid (2t)[9]

Following procedure E, colorless oil (81.3 mg, 99% yield, 90% ee); $[\alpha]_D^{20}$ = – 24.1 (*c* = 0.75, CH_2Cl_2); **^{1}H NMR** (400 MHz, Chloroform-*d*) δ 9.62 (br, 1H), 7.23 – 7.21 (m, 2H), 7.17 – 7.07 (m, 3H), 3.00 (dd, *J* = 13.5, 6.3 Hz, 1H), 2.77 – 2.65 (m, 1H), 2.59 (dd, *J* = 13.4, 7.9 Hz, 1H), 1.10 (d, *J* = 6.8 Hz, 3H); **^{13}C NMR** (101 MHz, Chloroform-*d*) δ 182.63, 139.18, 129.14, 128.55, 126.55, 41.52, 39.45, 16.63; HPLC conditions: DAICEL Chiralpak OJ-H column, Hexane/*i*PrOH = 99/1, 210 nm, 1.0 mL/min, 25 ^{o}C, t_{major} = 9.093 min, t_{minor} = 10.159 min.

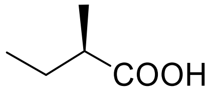

(*R*)-2-Methylbutanoic acid (2u)[9]

Following procedure E, colorless oil (47.4 mg, 93% yield, 94% ee); $[\alpha]_D^{20}$ = –16.3 (*c* = 0.38, CH_2Cl_2); **^{1}H NMR** (400 MHz, Chloroform-*d*) δ 2.47 – 2.46 (m, 1H), 1.76 – 1.64 (m, 1H), 1.57 – 1.43 (m, 1H), 1.17 (d, *J* = 7.0 Hz, 3H), 0.94 (t, *J* = 7.5 Hz, 3H); **^{13}C NMR** (101 MHz, Chloroform-*d*) δ 183.27, 41.01, 26.67, 16.50, 11.68; HPLC conditions: DAICEL Chiralpak AD-H column, Hexane/*i*PrOH = 98/2, 256 nm, 1.0 mL/min, 25 °C, t_{minor} = 32.759 min, t_{major} = 35.005 min.

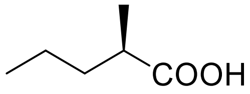

(*R*)-2-Methylpentanoic acid (2v)[15]

Following procedure G, colorless oil (55.1 mg, 95% yield, 94% ee); $[\alpha]_D^{20}$ = –41.5 (*c* = 0.31, CH_2Cl_2); **^{1}H NMR** (400 MHz, Chloroform-*d*) δ 2.53 – 2.38 (m, 1H), 1.73 – 1.60 (m, 1H), 1.48 – 1.29 (m, 3H), 1.17 (d, *J* = 7.0 Hz, 3H), 0.91 (t, *J* = 7.2 Hz, 3H); **^{13}C NMR** (101 MHz, Chloroform-*d*) δ 183.76, 39.32, 35.77, 20.44, 16.93, 14.05; HPLC conditions: DAICEL Chiralpak AD-H column, Hexane/*i*PrOH = 95/5, 256 nm, 1.0 mL/min, 25 °C, t_{minor} = 11.763 min, t_{major} = 12.984 min.

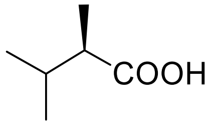

(*R*)-2,3-Dimethylbutanoic acid (2w)[16]

Following procedure E, colorless oil (54.5 mg, 94% yield, 91% ee); $[\alpha]_D^{20}$ = –25.8 (*c* = 0.58, CH_2Cl_2); **^{1}H NMR** (400 MHz, Chloroform-*d*) δ 2.30 – 2.22 (m, 1H), 2.03 – 1.87 (m, 1H), 1.13 (d, *J* = 7.0 Hz, 3H), 0.97 (d, *J* = 6.8 Hz, 3H), 0.93 (d, *J* = 6.8 Hz, 3H); ^{13}**C NMR** (101 MHz, Chloroform-*d*) δ 182.99, 46.14, 30.90, 20.80, 19.15, 13.52; HPLC conditions: DAICEL Chiralpak AD-H column, Hexane/*i*PrOH = 98/2, 256 nm, 1.0 mL/min, 25 °C, t_{minor} = 28.509 min, t_{major} = 36.631 min.

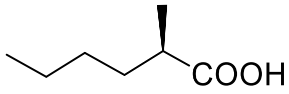

(*R*)-2-Methylhexanoic acid (2x)[15]

Following procedure G, colorless oil (62.4 mg, 96% yield, 95% ee); $[\alpha]_D^{20}$ = –16.8 (c = 0.49, CH_2Cl_2); **^{1}H NMR** (400 MHz, Chloroform-*d*) δ 2.49 – 2.39 (m, 1H), 1.74 – 1.62 (m, 1H), 1.53 – 1.37 (m, 1H), 1.36 – 1.27 (m, 4H), 1.17 (d, J = 7.0 Hz, 3H), 0.96 – 0.85 (m, 3H); **^{13}C NMR** (101 MHz, Chloroform-*d*) δ 183.84, 39.56, 33.37, 29.44, 22.71, 16.95, 14.04; HPLC conditions: DAICEL Chiralpak AD-H column, Hexane/*i*PrOH = 98/2, 256 nm, 1.0 mL/min, 25 ^oC, t_{minor} = 29.363 min, t_{major} = 33.076 min.

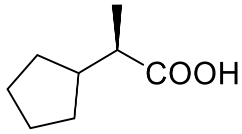

(*R*)-2-Cyclopentylpropanoic acid (2y)[17]

Following procedure E, colorless oil (67.5 mg, 95% yield, 91% ee); $[\alpha]_D^{20}$ = –16.7 (c = 1.12, CH_2Cl_2); **^{1}H NMR** (400 MHz, Chloroform-*d*) δ 2.30 – 2.23 (m, 1H), 2.07 – 1.90 (m, 1H), 1.87 – 1.73 (m, 2H), 1.66 – 1.46 (m, 4H), 1.35 – 0.97 (m, 5H); **^{13}C NMR** (101 MHz, Chloroform-*d*) δ 183.41, 45.02, 43.29, 31.06, 30.29, 25.30, 25.20, 16.12; HPLC conditions: DAICEL Chiralpak AD-H column, Hexane/*i*PrOH = 98/2, 256 nm, 1.0 mL/min, 25 ^oC, t_{minor} = 38.279 min, t_{major} = 49.647 min.

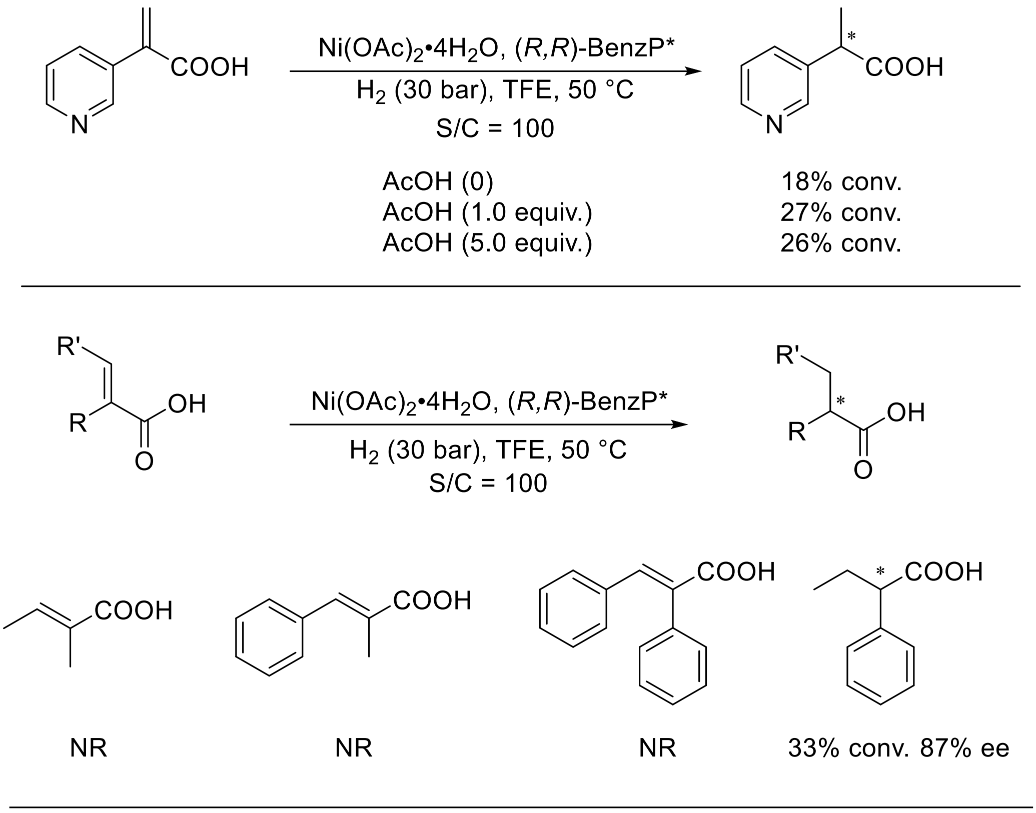

Supplementary Fig. 1 The heteroaromatic and trisubstituted substrates

Supplementary Note 2

S/C Hydrogenation and Transformations

S/C Hydrogenation

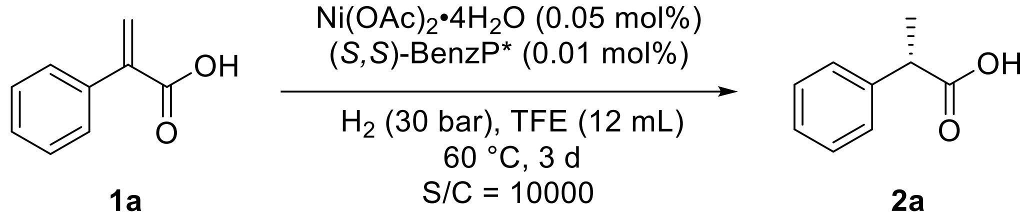

1a **2a**

6.00 g 0.59 g, 98% yield, 96% ee

Procedure H : Ni(OAc)$_2$·4H$_2$O (4.98 mg, 0.050 mol%), (*S,S*)-BenzP* (1.13 mg, 0.010 mol%), and substrate **1a** (6.00 g) were placed in a dried 50 mL thick wall flask. They were then transferred to a nitrogen-filled glovebox and degassed anhydrous trifluoroethanol (TFE, 12mL) was added. The reaction was stirred under H_2 (30 bar) at 60 ^{o}C in a stainless steel autoclave for 3 days. The resulting solution was concentrated under reduced pressure and the residue was purified by chromatography (DCM:MeOH = 8:1) on silica gel.

Transformations

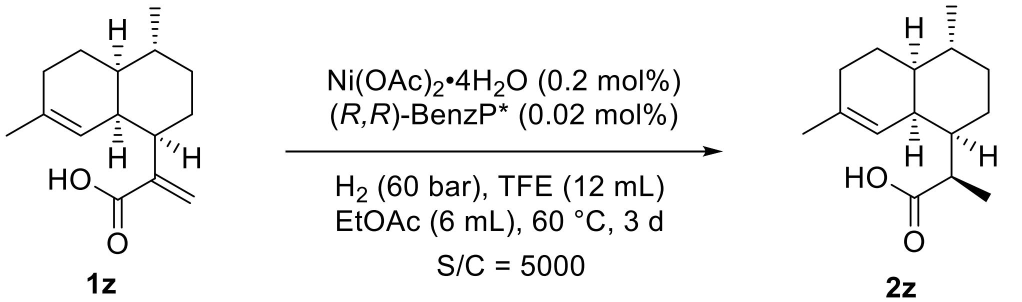

1z **2z**

4.70 g 4.65 g, 98% yield, 99.8 : 0.2 dr

Procedure I: Ni(OAc)$_2$·4H$_2$O (10.0 mg, 0.20 mol%), (*R,R*)-BenzP* (1.13 mg, 0.020 mol%) and substrate **1z** (4.70 g) were placed in a dried 50 mL thick wall flask. They were transferred to a nitrogen-filled glovebox, then degassed anhydrous trifluoroethanol (TFE, 12 mL) and ethyl acetate (EA, 6.0 mL) were added. The reaction was stirred under H_2 (60 bar) at 60 ^{o}C in a stainless steel

autoclave for 3 days. The hydrogen gas was released slowly and carefully. The resulting solution was concentrated under reduced pressure and the residue was purified by chromatography (DCM:MeOH = 8:1) on silica gel.

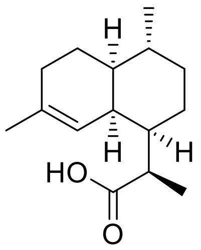

(*R*)-Dihydroartemisinic acid (DHAA) (2z)[9]

White solid (4.65 g, 98% yield, 99.8:0.2 dr); $[\alpha]_D^{20}$ = – 13.3 (*c* = 0.42, CH_2Cl_2); **^{1}H NMR** (400 MHz, Chloroform-*d*) δ 5.12 (s, 1H), 2.51 – 2.50 (m, 2H), 1.98 – 1.87 (m, 2H), 1.83 – 1.77 (m, 1H), 1.71 – 1.50 (m, 6H), 1.49 – 1.39 (m, 2H), 1.28 – 1.23 (m, 1H), 1.19 (d, *J* = 6.9 Hz, 3H), 1.13 – 1.04 (m, 1H), 1.01 – 0.92 (m, 1H), 0.87 (d, *J* = 6.5 Hz, 3H); **^{13}C NMR** (101 MHz, Chloroform-*d*) δ 183.94, 136.16, 119.46, 43.73, 42.38, 41.87, 36.49, 35.38, 27.80, 27.56, 26.76, 25.92, 23.97, 19.84, 15.22; HPLC conditions: Agilent Eclipse XDB-18 column, aqueous H_3PO_4 (0.1%)/MeCN = 50/50, 210 nm, 1.0 mL/min, 25 ^oC, t_{major} = 28.674 min, t_{minor} = 29.907 min.

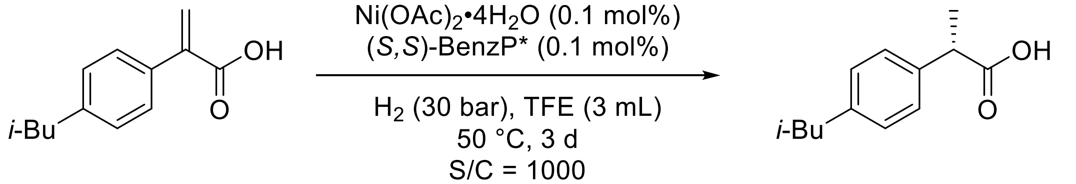

1l **2l**

0.61 g 0.60 g, 99% yield, 96% ee

Procedure J: $Ni(OAc)_2{\cdot}4H_2O$ (0.75 mg, 0.10 mol%), (*S,S*)-BenzP* (0.85 mg, 0.10 mol%) and substrate **1l** (0.61 g) were placed in a dried hydrogenation tube. They were transferred to a nitrogen-filled glovebox, then degassed anhydrous trifluoroethanol (TFE, 3.0 mL) was added. The reaction was stirred under H_2 (30 bar) at 50 ^oC in a stainless steel autoclave for 3 days. The hydrogen gas was released slowly and carefully. The resulting solution was concentrated under reduced pressure and the residue was purified by chromatography (DCM:MeOH = 8:1) on silica gel.

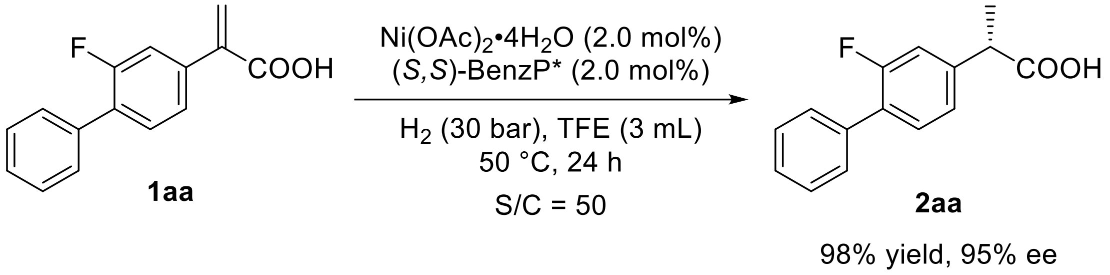

Procedure H: $Ni(OAc)_2{\cdot}4H_2O$ (0.50 mg, 2.0 mol%), (*S,S*)-BenzP* (0.56 mg, 2.0 mol%) and substrate **1aa** (24 mg) were placed in a dried hydrogenation tube. They were transferred to a nitrogen-filled glovebox, then degassed anhydrous trifluoroethanol (TFE, 3.0 mL) was added. The reaction was stirred under H_2 (30 bar) at 50 ^{o}C in a stainless steel autoclave for 24 h. The hydrogen gas was released slowly and carefully. The resulting solution was concentrated under reduced pressure and the residue was purified by chromatography (DCM:MeOH = 8:1) on silica gel.

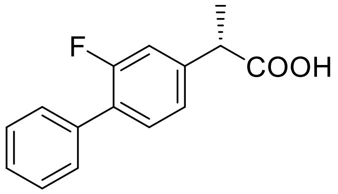

(*S*)-2-(2-Fluoro-[1,1'-biphenyl]-4-yl)propanoic acid (2aa)[8]

White solid (23.9 mg, 98% yield, 95% ee); $[\alpha]_D^{20}$ = + 27.5 (*c* = 0.26, CH_2Cl_2); **^{1}H NMR** (400 MHz, Chloroform-*d*) δ 7.72 – 7.50 (m, 2H), 7.49 – 7.31 (m, 4H), 7.21 – 7.12 (m, 2H), 3.80 – 3.79 (m 1H), 1.57 (d, *J* = 7.0 Hz, 3H); **^{13}C NMR** (101 MHz, Chloroform-*d*) *δ* 180.13, 160.13 (d, *J* = 248.5 Hz), 141.56, 135.87, 131.31 (d, *J* = 3.9 Hz), 129.40 (d, *J* = 2.8 Hz), 128.90, 128.55 (d, *J* = 13.2 Hz), 128.15, 124.16, 115.84 (d, *J* = 23.7 Hz), 45.33, 18.51; **^{19}F NMR** (376 MHz, Chloroform-*d*): *δ* -117.45; HPLC conditions: DAICEL Chiralpak OJ-H column, Hexane/*i*PrOH = 99/1, 210 nm, 1.0 mL/min, 25 ^{o}C, t_{minor} = 17.878 min, t_{major} = 21.941 min.

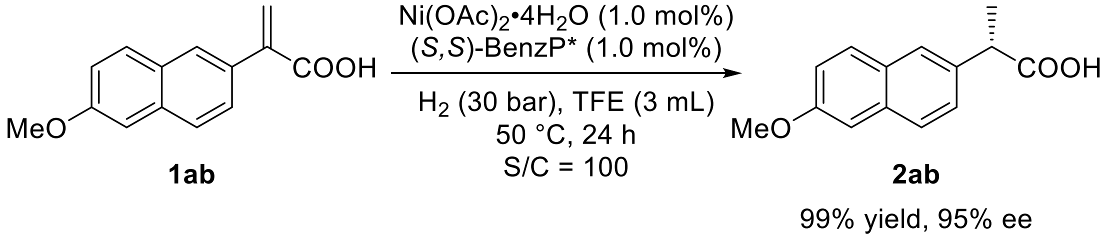

Procedure I: Ni(OAc)$_2$·4H$_2$O (0.50 mg, 1.0 mol%), (*S,S*)-BenzP* (0.56 mg, 1.0 mol%) and substrate **1ab** (46 mg) were placed in a dried hydrogenation tube. They were transferred to a nitrogen-filled glovebox, then degassed anhydrous trifluoroethanol (TFE, 3.0 mL) was added. The reaction was stirred under H$_2$ (30 bar) at 50 ^{o}C in a stainless steel autoclave for 24 h. The hydrogen gas was released slowly and carefully. The resulting solution was concentrated under reduced pressure and the residue was purified by chromatography (DCM:MeOH = 8:1) on silica gel.

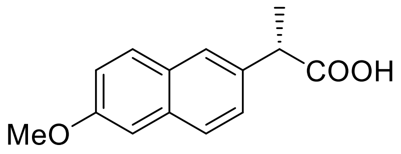

(*S*)-2-(6-Methoxynaphthalen-2-yl)propanoic acid (2ab)[9]

White solid (45.6 mg, 99% yield, 95% ee); $[\alpha]_D^{20}$ = + 53.9 (*c* = 0.26, CH$_2$Cl$_2$); **^{1}H NMR** (400 MHz, Chloroform-*d*) *δ* 7.73 – 7.66 (m, 3H), 7.43 – 7.37 (m, 1H), 7.21 – 7.06 (m, 2H), 4.02 – 3.47 (m, 4H), 1.57 (d, *J* = 7.1 Hz, 3H); **^{13}C NMR** (101 MHz, Chloroform-*d*) *δ* 180.96, 157.80, 135.07, 133.92, 129.43, 129.02, 127.34, 126.34, 126.27, 119.13, 105.71, 55.40, 45.46, 18.27; HPLC conditions: DAICEL Chiralpak AD-H column, Hexane/*i*PrOH = 99/1, 210 nm, 1.0 mL/min, 25 ^{o}C, t_{major} = 11.616 min, t_{minor} = 12.751 min.

Supplementary Note 3

Mechanism Study

Deuterium-labeling experiments

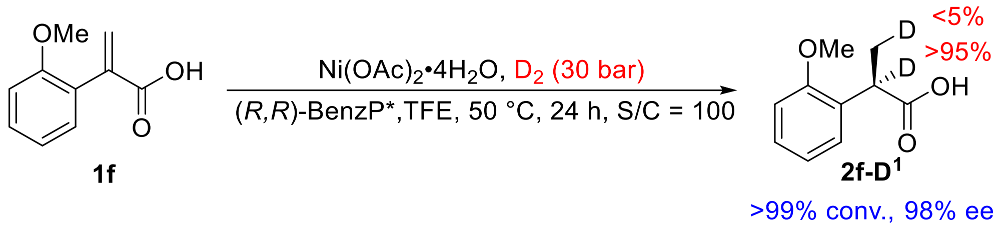

Supplementary Fig. 2 The Deuterium-labeling experiments using D_2

Procedure J : $Ni(OAc)_2 \cdot 4H_2O$ (0.50 mg, 1.0 mol%), (*R*,*R*)-BenzP* (0.56 mg, 1.0 mol%) and substrate **1f** (35.6 mg, S/C = 100) were placed in a dried Schlenk tube. The tube was then transferred to a nitrogen-filled glovebox and degassed anhydrous trifluoroethanol (TFE, 1.0 mL) was added. The reaction was stirred under D_2 (30 bar) at 50 °C in a stainless steel autoclave for 24 h. The crude product was obtained by column chromatography on silica gel (eluant: EA). Then the reaction mixture was subjected to proton NMR analysis in $CDCl_3$ after the solvent was evaporated under reduced pressure.

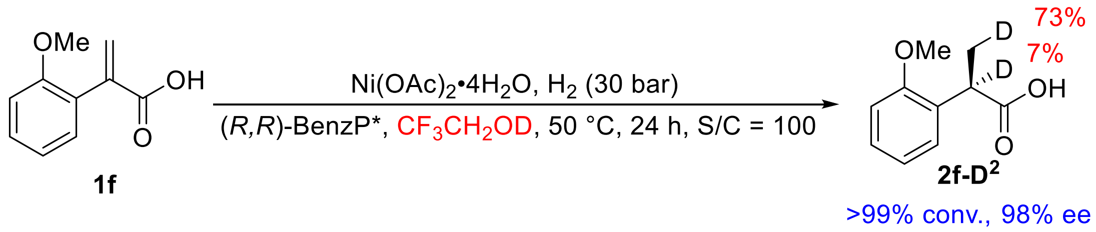

Supplementary Fig. 3 The Deuterium-labeling experiments using CF_3CH_2OD

Procedure K : $Ni(OAc)_2 \cdot 4H_2O$ (0.50 mg, 1.0 mol%), (*R*,*R*)-BenzP* (0.56 mg, 1.0 mol%) and substrate **1f** (35.6 mg, S/C = 100) were placed in a dried Schlenk tube. The tube was then transferred to a nitrogen-filled glovebox and CF_3CH_2OD (1.0 mL) was added. The reaction was stirred under H_2 (30 bar) at 50 °C in a stainless steel autoclave for 24 h. The crude product was obtained by column chromatography on silica gel (eluant: EA). Then the reaction mixture

was subjected to proton NMR analysis in $CDCl_3$ after the solvent was evaporated under reduced pressure.

Control experiments

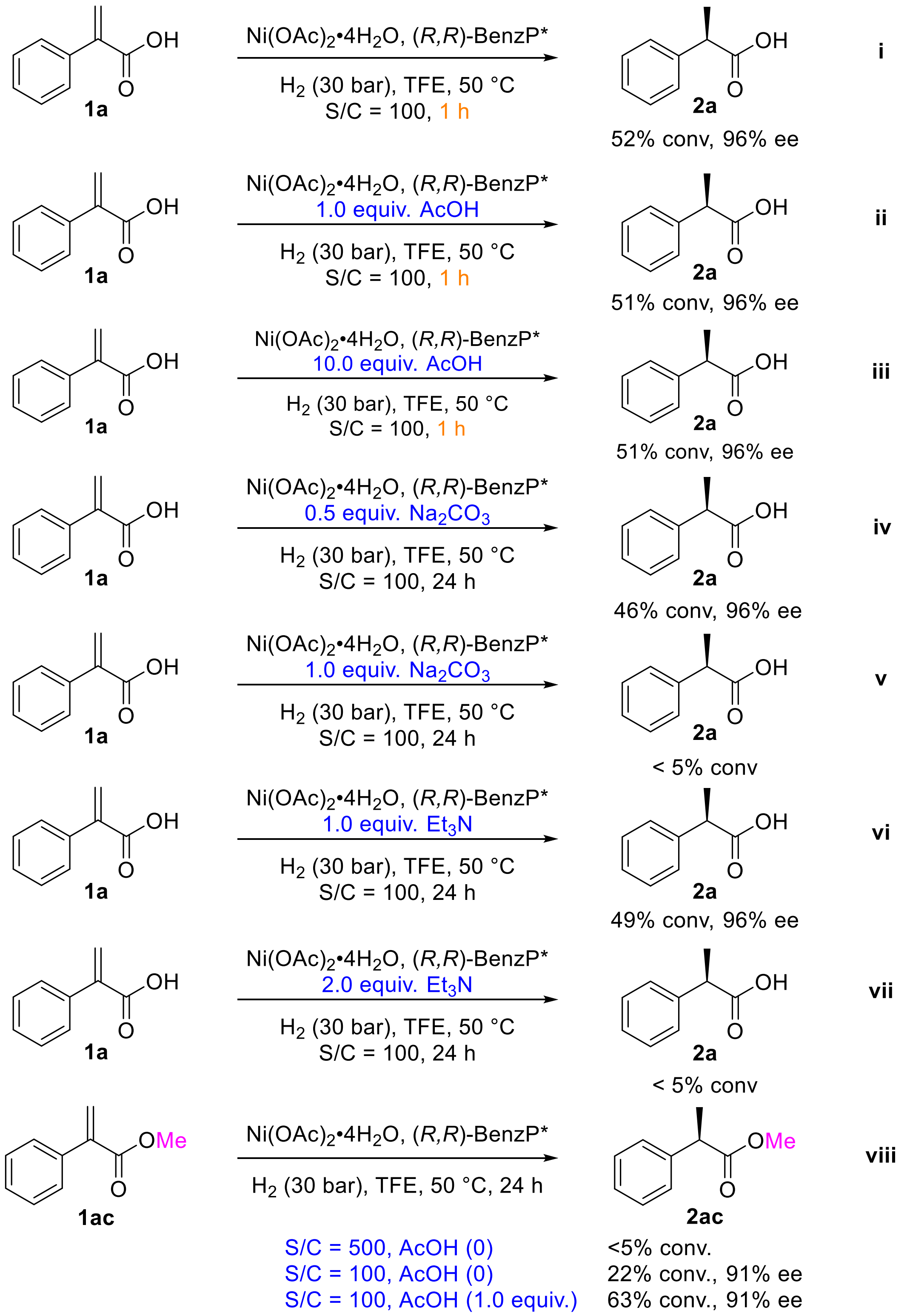

Supplementary Fig. 4 The control experiments of the Ni-catalyzed asymmetric hydrogenation of **1a** and **1ac**

i: $Ni(OAc)_2 \cdot 4H_2O$ (0.50 mg, 1.0 mol%), (*R,R*)-BenzP* (0.56 mg, 1.0 mol%) and substrate **1a** (S/C = 100) were placed in a dried Schlenk tube. The tube was then transferred to a nitrogen-filled glovebox and degassed anhydrous trifluoroethanol (TFE, 1.0 mL) was added. The reaction was stirred under H_2 (30 bar) at 50 ^{o}C in a stainless steel autoclave for 1 h. The crude product was obtained by column chromatography on silica gel (eluant: EA). Then the reaction mixture was subjected to proton NMR analysis in $CDCl_3$ after the solvent was evaporated under reduced pressure.

ii: $Ni(OAc)_2 \cdot 4H_2O$ (0.50 mg, 1.0 mol%), (*R,R*)-BenzP* (0.56 mg, 1.0 mol%) and substrate **1a** (S/C = 100) were placed in a dried Schlenk tube. The tube was then transferred to a nitrogen-filled glovebox and degassed anhydrous trifluoroethanol (TFE, 1.0 mL) was added, then degassed anhydrous AcOH (11 μL, 1.0 equiv.) added to the tube. The reaction was stirred under H_2 (30 bar) at 50 ^{o}C in a stainless steel autoclave for 1 h. The crude product was obtained by column chromatography on silica gel (eluant: EA). Then the reaction mixture was subjected to proton NMR analysis in $CDCl_3$ after the solvent was evaporated under reduced pressure.

iii: $Ni(OAc)_2 \cdot 4H_2O$ (0.50 mg, 1.0 mol%), (*R,R*)-BenzP* (0.56 mg, 1.0 mol%) and substrate **1a** (S/C = 100) were placed in a dried Schlenk tube. The tube was then transferred to a nitrogen-filled glovebox and degassed anhydrous trifluoroethanol (TFE, 1.0 mL) was added, then degassed anhydrous AcOH (114 μL, 10 equiv.) added to the tube. The reaction was stirred under H_2 (30 bar) at 50 ^{o}C in a stainless steel autoclave for 1 h. The crude product was obtained by column chromatography on silica gel (eluant: EA). Then the reaction mixture was subjected to proton NMR analysis in $CDCl_3$ after the solvent was evaporated under reduced pressure.

iv: $Ni(OAc)_2 \cdot 4H_2O$ (0.50 mg, 1.0 mol%), (*R,R*)-BenzP* (0.56 mg, 1.0 mol%), Na_2CO_3 (33 mg, 0.50 eq) and substrate **1a** (S/C = 100) were placed in a dried Schlenk tube. The tube was then transferred to a nitrogen-filled glovebox and

degassed anhydrous trifluoroethanol (TFE, 1.0 mL) was added. The reaction was stirred under H_2 (30 bar) at 50 ^oC in a stainless steel autoclave for 24 h. After reaction was completed, the mixture was acidified to pH = 1 with concentrated HCl. The resulting solution was extracted with EtOAc (2.0 mL). The combined organic phase was dried over anhydrous Na_2SO_4, and solvent was removed under reduced pressure. Then the reaction mixture was subjected to proton NMR analysis in $CDCl_3$.

v: $Ni(OAc)_2{\cdot}4H_2O$ (0.50 mg, 1.0 mol%), (*R,R*)-BenzP* (0.56 mg, 1.0 mol%), Na_2CO_3 (65 mg, 1.0 equiv.) and substrate **1a** (S/C = 100) were placed in a dried Schlenk tube. The tube was then transferred to a nitrogen-filled glovebox and degassed anhydrous trifluoroethanol (TFE, 1.0 mL) was added. The reaction was stirred under H_2 (30 bar) at 50 ^oC in a stainless steel autoclave for 24 h. After reaction was completed, the mixture was acidified to pH = 1 with concentrated HCl. The resulting solution was extracted with EtOAc (2.0 mL). The combined organic phase was dried over anhydrous Na_2SO_4, and solvent was removed under reduced pressure. Then the reaction mixture was subjected to proton NMR analysis in $CDCl_3$.

vi: $Ni(OAc)_2{\cdot}4H_2O$ (0.50 mg, 1.0 mol%), (*R,R*)-BenzP* (0.56 mg, 1.0 mol%) and substrate **1a** (S/C = 100) were placed in a dried Schlenk tube. The tube was then transferred to a nitrogen-filled glovebox and degassed anhydrous trifluoroethanol (TFE, 1.0 mL) was added. Then degassed anhydrous Et_3N (27 μL, 1.0 equiv.) was added to the tube. The reaction was stirred under H_2 (30 bar) at 50 ^oC in a stainless steel autoclave for 24 h. After the reaction was completed, the mixture was acidified to pH = 1 with concentrated HCl. The resulting solution was extracted with EtOAc (2.0 mL). The combined organic phase was dried over anhydrous Na_2SO_4, and solvent was removed under reduced pressure. Then the reaction mixture was subjected to proton NMR analysis in $CDCl_3$.

vii: $Ni(OAc)_2{\cdot}4H_2O$ (0.50 mg, 1.0 mol%), (*R,R*)-BenzP* (0.56 mg, 1.0 mol%) and substrate **1a** (S/C = 100) were placed in a dried Schlenk tube. The tube was then transferred to a nitrogen-filled glovebox and degassed anhydrous trifluoroethanol (TFE, 1.0 mL) was added. Then degassed anhydrous Et_3N (56

μL, 2.0 equiv.) was added to the tube. The reaction was stirred under H_2 (30 bar) at 50 ^oC in a stainless steel autoclave for 24 h. After reaction was completed, the mixture was acidified to pH = 1 with concentrated HCl. The resulting solution was extracted with EtOAc (2.0 mL). The combined organic phase was dried over anhydrous Na_2SO_4, and solvent was removed under reduced pressure. Then the reaction mixture was subjected to proton NMR analysis in $CDCl_3$.

viii: $Ni(OAc)_2{\cdot}4H_2O$ (0.50 mg, 0.20 mol%), (*R,R*)-BenzP* (0.56 mg, 0.20 mol%) and substrate **1ac** (S/C = 500) were placed in a dried Schlenk tube. The tube was then transferred to a nitrogen-filled glovebox and degassed anhydrous trifluoroethanol (TFE, 1.0 mL) was added. The reaction was stirred under H_2 (30 bar) at 50 ^oC in a stainless steel autoclave for 24 h. The crude product was obtained by column chromatography on silica gel (eluant: EA). Then the reaction mixture was subjected to proton NMR analysis in $CDCl_3$, after the solvent was evaporated under reduced pressure.

$Ni(OAc)_2{\cdot}4H_2O$ (0.50 mg, 1.0 mol%), (*R,R*)-BenzP* (0.56 mg, 1.0 mol%) and substrate **1a** (S/C = 100) were placed in a dried Schlenk tube. The tube was then transferred to a nitrogen-filled glovebox and degassed anhydrous trifluoroethanol (TFE, 1.0 mL) was added. The reaction was stirred under H_2 (30 bar) at 50 ^oC in a stainless steel autoclave for 24 h. The crude product was obtained by column chromatography on silica gel (eluant: EA). Then the reaction mixture was subjected to proton NMR analysis in $CDCl_3$, after the solvent was evaporated under reduced pressure.

$Ni(OAc)_2{\cdot}4H_2O$ (0.50 mg, 1.0 mol%), (*R,R*)-BenzP* (0.56 mg, 1.0 mol%) and substrate **1ac** (S/C = 100) were placed in a dried Schlenk tube. The tube was then transferred to a nitrogen-filled glovebox and degassed anhydrous trifluoroethanol (TFE, 1.0 mL) was added. Then degassed anhydrous AcOH (11 μL, 1.0 equiv.) was also added. The reaction was stirred under H_2 (30 bar) at 50 ^oC in a stainless steel autoclave for 24 h. The crude product was obtained by column chromatography on silica gel (eluant: EA). Then the reaction mixture was subjected to proton NMR analysis in $CDCl_3$, after the solvent was evaporated under reduced pressure.

Determination of reaction order

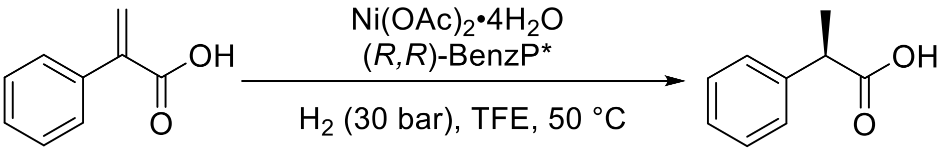

1a **2a**

Procedure for 0.250 mol% catalyst: $Ni(OAc)_2 \cdot 4H_2O$ (2.50 mg, 0.250 mol%), (*R,R*)-BenzP* (2.80 mg, 0.250 mol%) and substrate **1a** (S/C = 400) were placed in a stainless steel autoclave. The autoclave was then transferred to a nitrogen-filled glovebox and degassed anhydrous trifluoroethanol (TFE, 14.0 mL) was added. When the reaction was stirred under H_2 (30 bar) at 50 ^{o}C for the indicated time, 0.20 mL solution was taken out of the autoclave. After cooling to room temperature, the solvent was removed under reduced pressure. Then the reaction mixture was subjected to proton NMR analysis in $CDCl_3$.

Procedure for 0.125 mol% catalyst: $Ni(OAc)_2 \cdot 4H_2O$ (1.25 mg, 0.125 mol%), (*R,R*)-BenzP* (1.40 mg, 0.125 mol%) and substrate **1a** (S/C = 800) were placed in a stainless steel autoclave. The autoclave was then transferred to a nitrogen-filled glovebox and degassed anhydrous trifluoroethanol (TFE, 14.0 mL) was added. When the reaction was stirred under H_2 (30 bar) at 50 ^{o}C for the indicated time, 0.20 mL solution was taken out of autoclave. After cooling to room temperature, the solvent was removed under reduced pressure. Then the reaction mixture was subjected to proton NMR analysis in $CDCl_3$.

Supplementary Table 2 The experimental data of 0.250 mol% catalyst

T (min)	T (h)	Conversion (%)	[**2a**] (M)
30	0.50	8	0.024
45	0.75	11	0.031
60	1.0	12	0.036
80	1.3	16	0.046
100	1.7	20	0.058
120	2.0	21	0.062
150	2.5	28	0.080

180	3.0	29	0.083
300	5.0	45	0.13
420	7.0	57	0.16
660	11	72	0.21
1440	24	100	0.29

Supplementary Table 3 The experimental data of 0.125 mol% catalyst

T (min)	T (h)	Conversion (%)	[**2a**] (M)
150	2.5	7	0.021
180	3.0	9	0.026
300	5.0	19	0.056
360	6.0	23	0.067
480	8.0	29	0.084
600	10	35	0.10
720	12	44	0.13
1440	24	66	0.19
2880	48	98	0.28

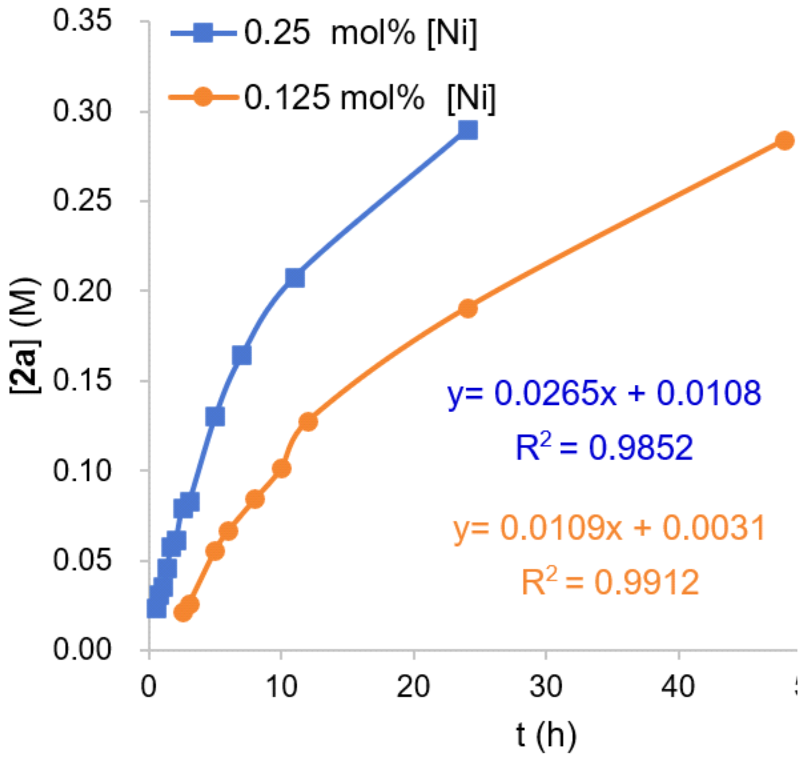

Supplementary Fig. 5 The reaction order of catalyst

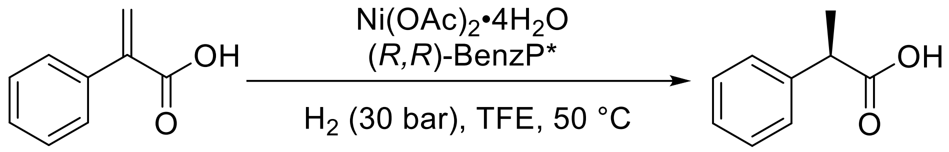

1a **2a**

Procedure for 0.290 M of 1a: Ni(OAc)$_2$·4H$_2$O (2.50 mg, 0.250 mol%), (*R,R*)-BenzP* (2.80 mg, 0.250 mol%) and substrate **1a** (S/C = 400) were placed in a stainless steel autoclave. The autoclave was then transferred to a nitrogen-filled glovebox and degassed anhydrous trifluoroethanol (TFE, 14.0 mL) was added. When the reaction was stirred under H_2 (30 bar) at 50 ^{o}C for the indicated time, 0.20 mL solution was taken out of the autoclave. After cooling to room temperature, the solvent was removed under reduced pressure. Then the reaction mixture was subjected to proton NMR analysis in $CDCl_3$.

Procedure for 0.140 M of 1a: Ni(OAc)$_2$·4H$_2$O (2.50 mg, 0.500 mol%), (*R,R*)-BenzP* (2.80 mg, 0.500 mol%) and substrate **1a** (S/C = 200) were placed in a stainless steel autoclave. The autoclave was then transferred to a nitrogen-filled glovebox and degassed anhydrous trifluoroethanol (TFE, 14.0 mL) was added. When the reaction was stirred under H_2 (30 bar) at 50 ^oC for the indicated time, 0.20 mL solution was taken out of autoclave. After cooling to room temperature, the solvent was removed under reduced pressure. Then the reaction mixture was subjected to proton NMR analysis in $CDCl_3$.

Supplementary Table 4 The experimental data of 0.290 M of **1a**

T (min)	T (h)	Conversion (%)	[**2a**] (M)
30	0.50	8	0.024
45	0.75	11	0.031
60	1.0	12	0.036
80	1.3	16	0.046
100	1.7	20	0.058
120	2.0	21	0.062
150	2.5	28	0.080
180	3.0	29	0.083
300	5.0	45	0.13
420	7.0	57	0.16
660	11	72	0.21
1440	24	100	0.29

Supplementary Table 5 The experimental data of 0.140 M of **1a**

T (min)	T (h)	Conversion (%)	[**2a**] (M)
45	0.75	6	0.0080
60	1.0	7	0.010
100	1.7	17	0.024
120	2.0	21	0.029
150	2.5	25	0.035
180	3.0	31	0.043

240	4.0	39	0.055
390	6.5	58	0.082
540	9.0	80	0.11
1440	24	100	0.14

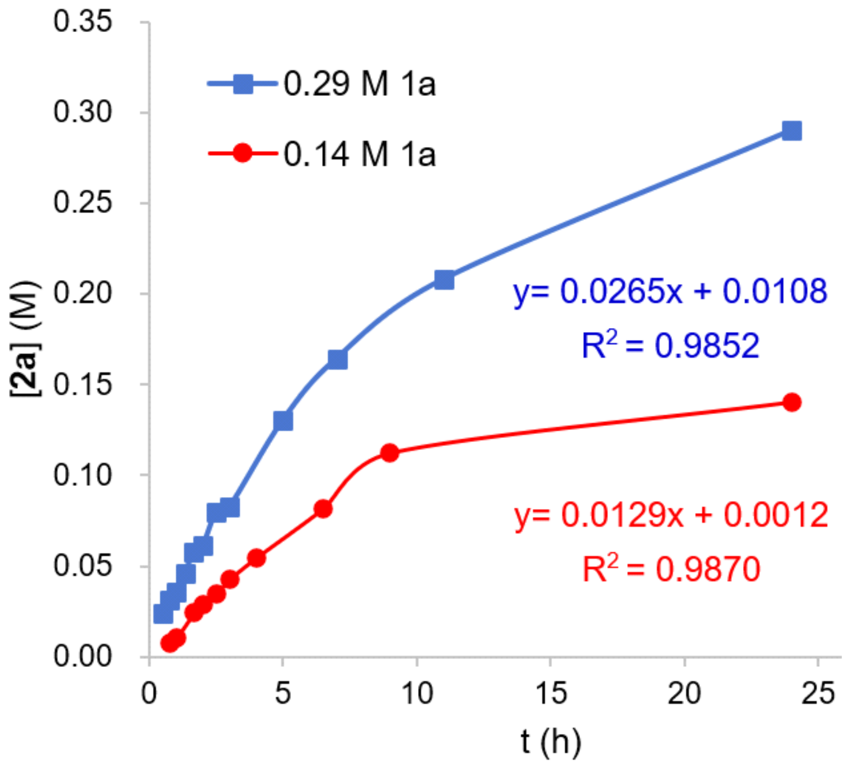

Supplementary Fig. 6 The reaction order of substrate

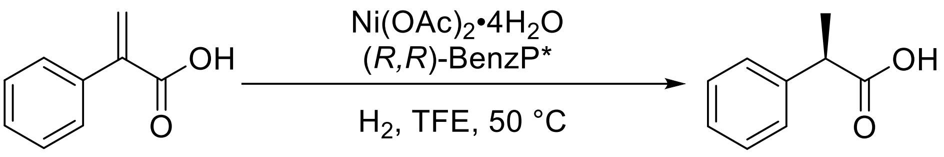

1a **2a**

Procedure for H_2 (30 bar): $Ni(OAc)_2 \cdot 4H_2O$ (2.50 mg, 0.250 mol%), (*R,R*)-BenzP* (2.80 mg, 0.250 mol%) and substrate **1a** (S/C = 400) were placed in a stainless steel autoclave. The autoclave was then transferred to a nitrogen-filled glovebox and degassed anhydrous trifluoroethanol (TFE, 14.0 mL) was added. When the reaction was stirred under H_2 (30 bar) at 50 ^oC for the indicated time, 0.20 mL solution was taken out of the autoclave. After cooling

to room temperature, the solvent was removed under reduced pressure. Then the reaction mixture was subjected to proton NMR analysis in $CDCl_3$.

Procedure for H_2 (15 bar): $Ni(OAc)_2 \cdot 4H_2O$ (2.50 mg, 0.250 mol%), (*R,R*)-BenzP* (2.80 mg, 0.250 mol%) and substrate **1a** (S/C = 400) were placed in a stainless steel autoclave. The autoclave was then transferred to a nitrogen-filled glovebox and degassed anhydrous trifluoroethanol (TFE, 14.0 mL) was added. When the reaction was stirred under H_2 (15 bar) at 50 ^oC for the indicated time, 0.20 mL solution was taken out of the autoclave. After cooling to room temperature, the solvent was removed under reduced pressure. Then the reaction mixture was subjected to proton NMR analysis in $CDCl_3$.

Supplementary Table 6 The experimental data at hydrogen pressure of 30 bar

T (min)	T (h)	Conversion (%)	[**2a**] (M)
30	0.50	8	0.024
45	0.75	11	0.031
60	1.0	12	0.036
80	1.3	16	0.046
100	1.7	20	0.058
120	2.0	21	0.062
150	2.5	28	0.080
180	3.0	29	0.083
300	5.0	45	0.13
420	7.0	57	0.16
660	11	72	0.21
1440	24	100	0.29

Supplementary Table 7 The experimental data at hydrogen pressure of 15 bar

T (min)	T (h)	Conversion (%)	[**2a**] (M)
50	0.83	5	0.014
90	1.5	12	0.033
110	1.8	14	0.040
130	2.2	16	0.046

160	2.7	18	0.052
310	5.2	38	0.11
430	7.2	46	0.13
670	11	62	0.18
1440	24	100	0.29

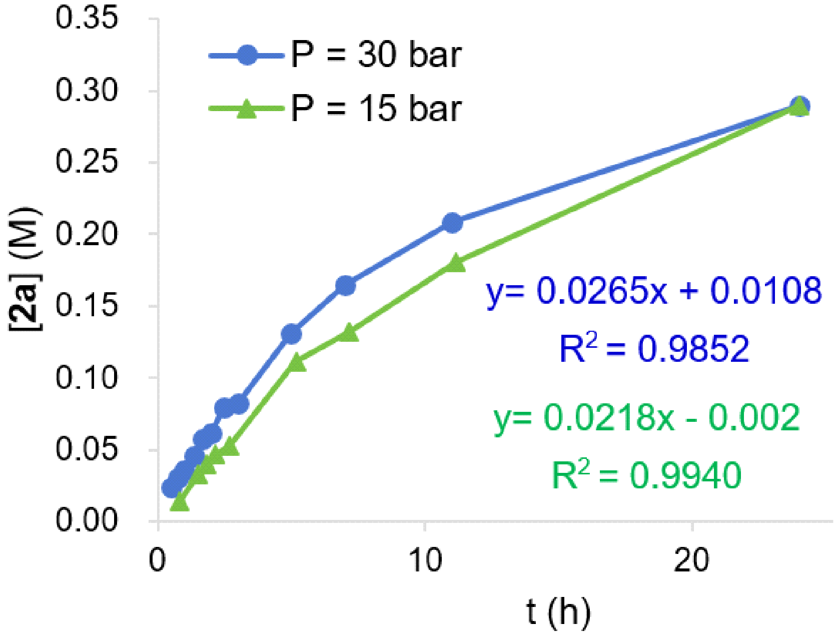

Supplementary Fig. 7 The reaction order of hydrogen pressure

Supplementary Note 4

X-Ray

The absolute configurations of the product **2f** was determined to be *R* by X-ray crystallographic analysis.

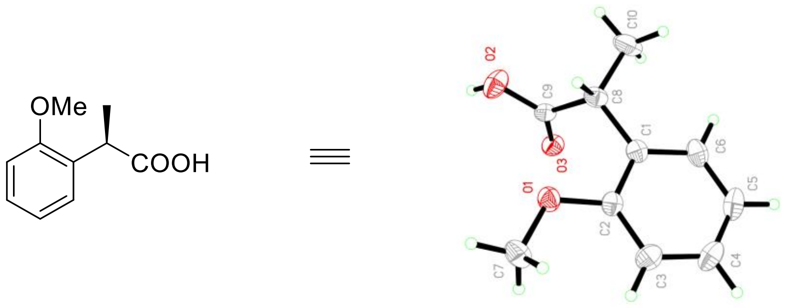

2f CCDC 2313709

Bond precision: C-C = 0.0024 A Wavelength=1.54178

Cell:	a=14.3867(3)	b=7.3079(2)	c=8.9729(2)
	alpha=90	beta=90	gamma=90
Temperature:	173 K		

	Calculated	Reported
Volume	943.38(4)	943.38(4)
Space group	P 21 21 2	P 21 21 2
Hall group	P 2 2ab	P 2 2ab
Moiety formula	C10 H12 O3	C10 H12 O3
Sum formula	C10 H12 O3	C10 H12 O3
Mr	180.20	180.20
Dx,g cm-3	1.269	1.269
Z	4	4
Mu (mm-1)	0.771	0.771
F000	384.0	384.0
F000'	385.27	
h,k,lmax		17,8,10
Nref		1731
Tmin,Tmax	0.857,0.884	0.697,0.753
Tmin'	0.857	

Correction method= # Reported T Limits: Tmin=0.697 Tmax=0.753
AbsCorr = MULTI-SCAN

Data completeness= Theta(max)= 68.184

R(reflections)= 0.0279(1670) wR2(reflections)= 0.0774(1731)

S = 1.046 Npar= 125

Supplementary Fig. 8 X-ray crystallographic analysis of **2f**

Supplementary Note 5

DFT Computations

Computational methods

Computations were performed using the Gaussian 09 (revision D.01) suite of quantum chemical program. All structures are optimized in an implicit solvent model using the PBE0[18] hybrid functional with usage of empirical Grimme's dispersion correction[19,20] (GD3-BJ). In optimization, all atoms are described with Ahlrichs's def2- series basis sets, def2-SVP[21] (a double-ζ basis set). All structures are in a local minimum potential energy surface with zero imaginary frequency or at the first order saddle point (transition state) on the potential surface with one imaginary frequency. Transition states (TSs) are calculated by the Berny algorithm.[22] Intrinsic reaction coordinate (IRC) calculations are additionally carried out to further characterize the true nature of some TSs.[23] Harmonic vibrational frequencies, thermal, and entropic corrections at 323.15K and $p^{\ominus}$ of all molecules. A global multiplicative harmonic frequency scaling factor for PBE0/def2-SVP basis set, 0.9915[24], were used as a correction for calculated harmonic frequencies and thermal data. The contribution of low frequency vibration is modified by quas*i*-harmonic approximation proposed by Grimme[25] considering the contribution of low frequency vibration (<100.0 cm^{-1}) to the partition function, using the free-rotor approximation. For those above this threshold, the RRHO approximation is retained. All frequency and thermal corrections are computed using Shermo.[26] The effect of a solvent continuum, in TFE, was evaluated using the Cramer−Truhlar continuum solvation model that describes the electrostatic interaction and nonpolar interaction between solvent and solute, named as SMD.[27] SMD model was used in geometry optimization and energy calculation.

Electron energies and Gibbs free energies.

Gibbs free energies were a sum of the electron energies in gas phase, the solvation free energies, and the thermal correction at 323.15K.

Supplementary Table 8 Electron energies and Gibbs free energies.

Structure number	Electron energies (a.u.)	Thermal Correction to free energies (a.u.)	Free energies (a.u.)	Free energies (kcal/mol)
**1a**	-497.320529	0.109115	-497.211414	-312004.87
Ni-H	-2816.071936	0.351285	-2815.720651	-1766891.38
IM-1	-3313.462178	0.491213	-3312.970965	-2078920.67
IM-2R	-3313.467009	0.491647	-3312.975362	-2078923.43
IM-2S	-3313.469273	0.493444	-3312.975829	-2078923.72
IM-3-1R	-3765.481043	0.548595	-3764.932448	-2362530.78
IM-3-1S	-3765.482528	0.547044	-3764.935484	-2362532.69
Sub	-497.320529	0.109115	-497.211414	-312004.87
TFE	-451.992315	0.024635	-451.967680	-283614.00
TS-1R	-3313.457630	0.488210	-3312.969420	-2078919.70
TS-1S	-3313.457634	0.489510	-3312.968124	-2078918.89
TS-2-1R	-3765.460378	0.547236	-3764.913142	-2362518.67
TS-2-1S	-3765.454357	0.544523	-3764.909834	-2362516.59

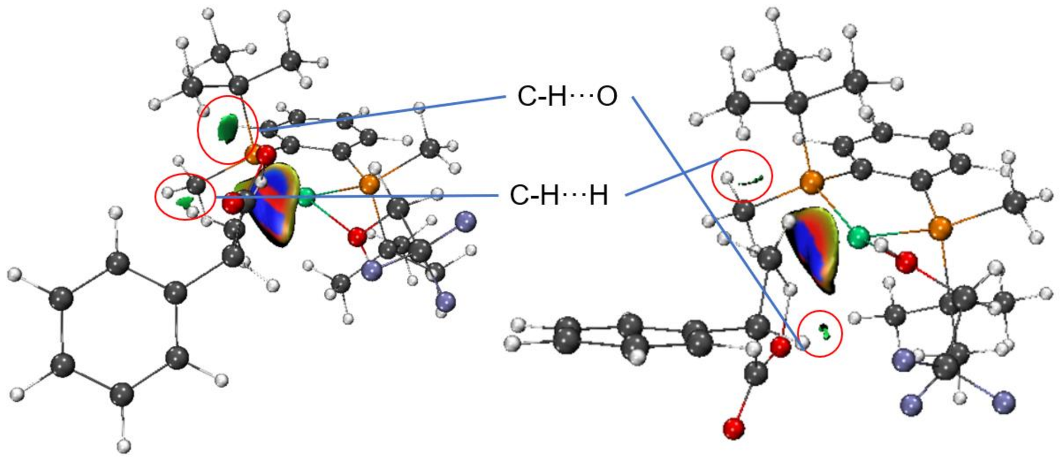

TS-2R **TS-2S**

Supplementary Fig. 9 Independent gradient model based on Hirshfeld partition analysis of **TS-2R** and **TS-2S**.

We used independent gradient model based on Hirshfeld partition (IGMH) analysis[28] to provide the visualization of the secondary interactions between substrates and Ni catalysts species in **TS-2R** and **TS-2S** using Multiwfn,[29] in which the isovalue of the surface is 0.014. As the Supplementary Fig 9 shows, C-H···H-C and C-H···O interactions are found in both transition states. However, in **TS-2S** these interactions are too weak to be observed compared with those in **TS-2R**. Since **TS-2R** leads to major product, this analysis suggests that these weak interactions may participate in stabilizing transition state and enhancing enantioselectivity.

Source data of Cartesian coordinates of the optimized structures are provided with this paper.

Supplementary Figure

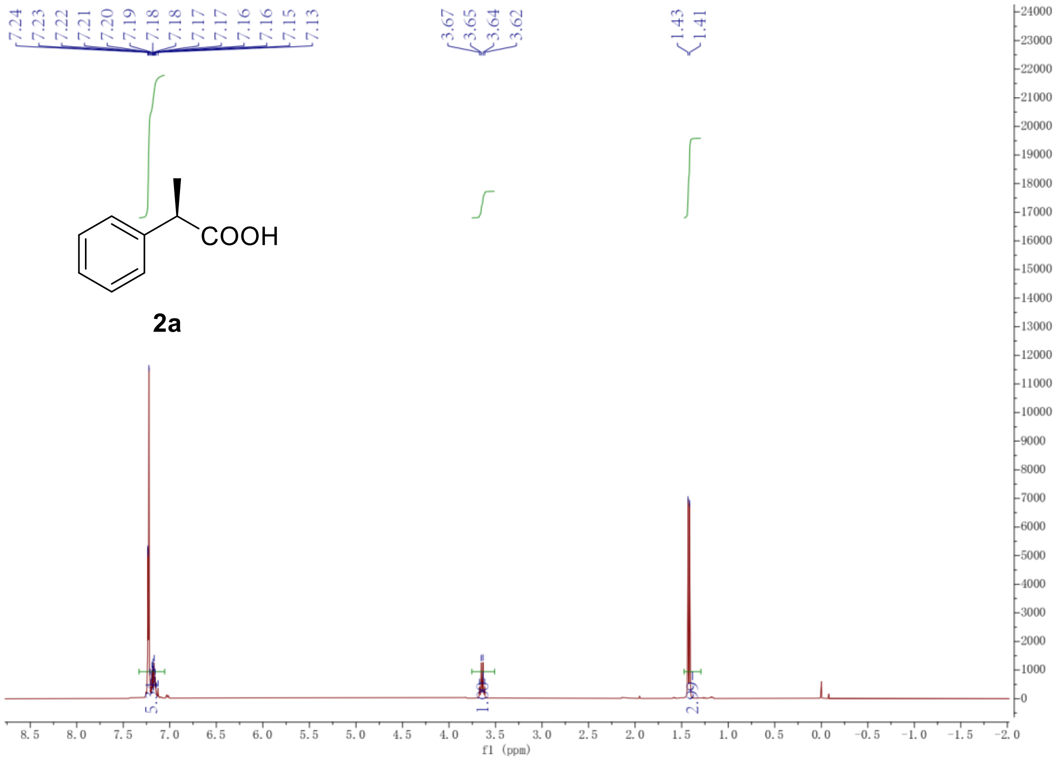

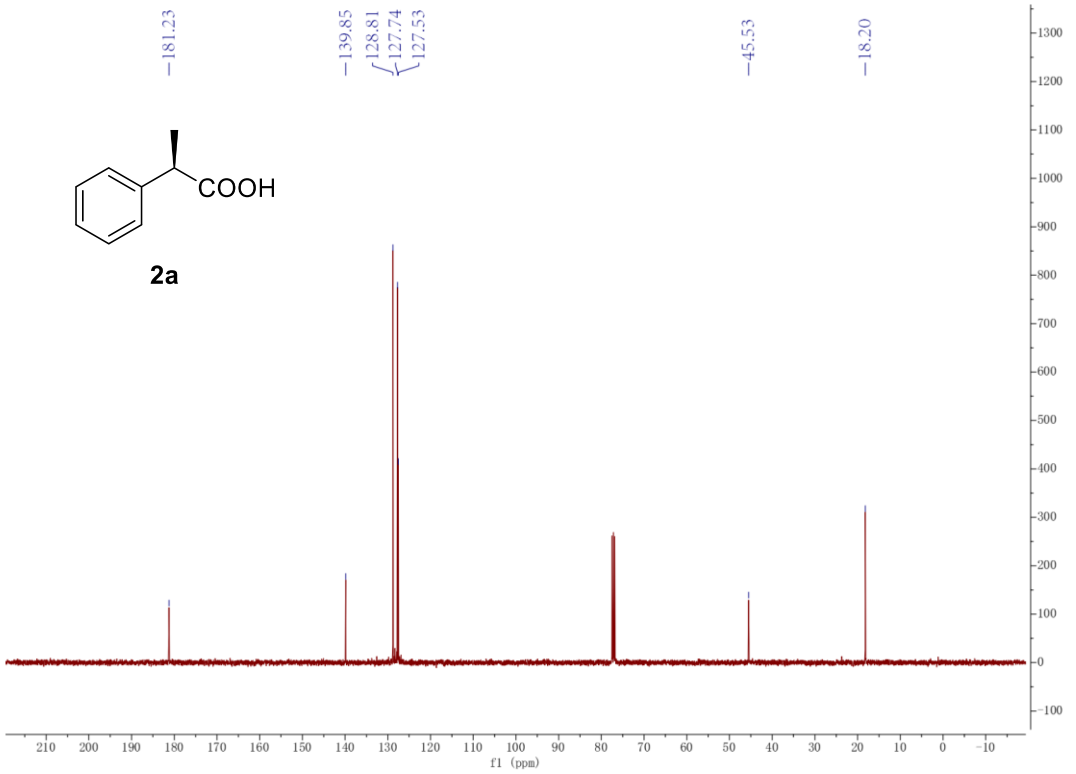

Supplementary Fig. 10 ^{1}H NMR (400 MHz) & ^{13}C NMR (101 MHz) spectra of compound **2a** in $CDCl_3$.

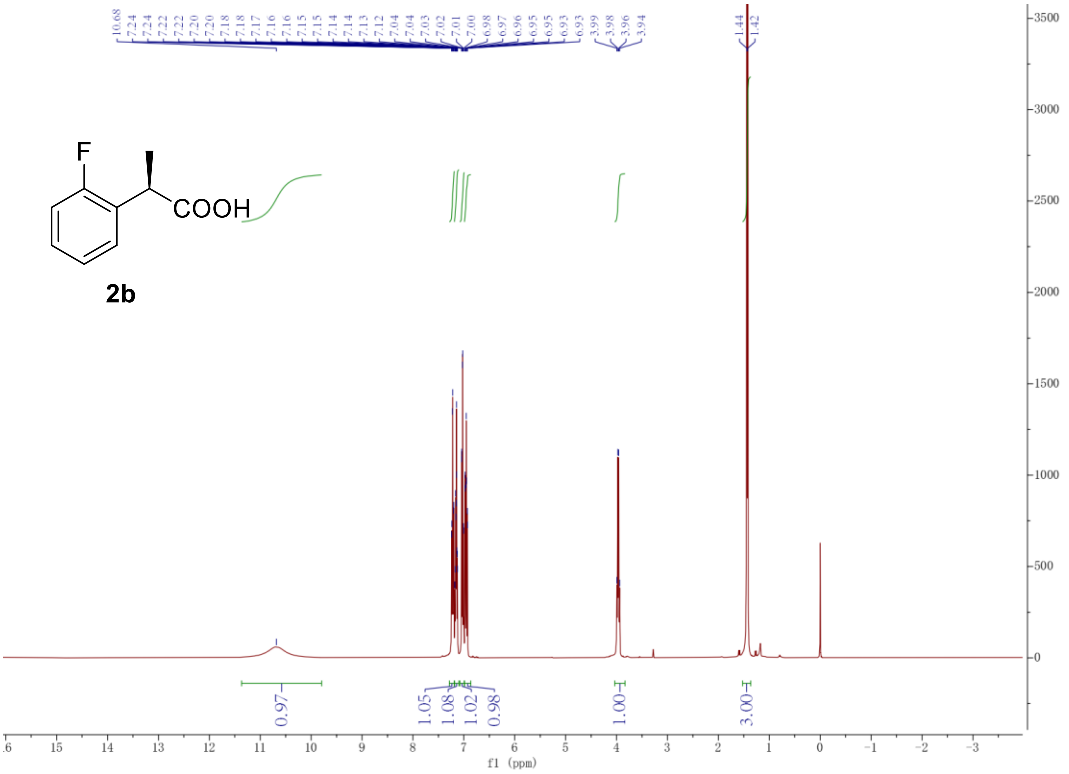
COOH
F
2b
f1 (ppm)

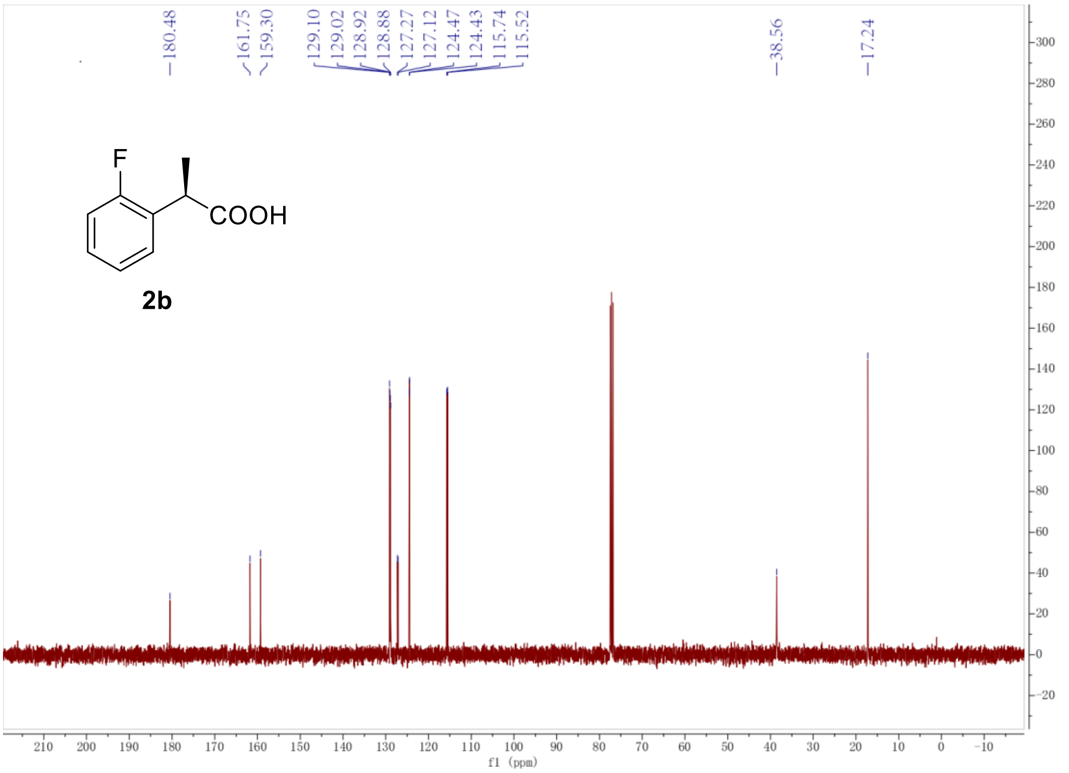
COOH
F
2b
f1 (ppm)

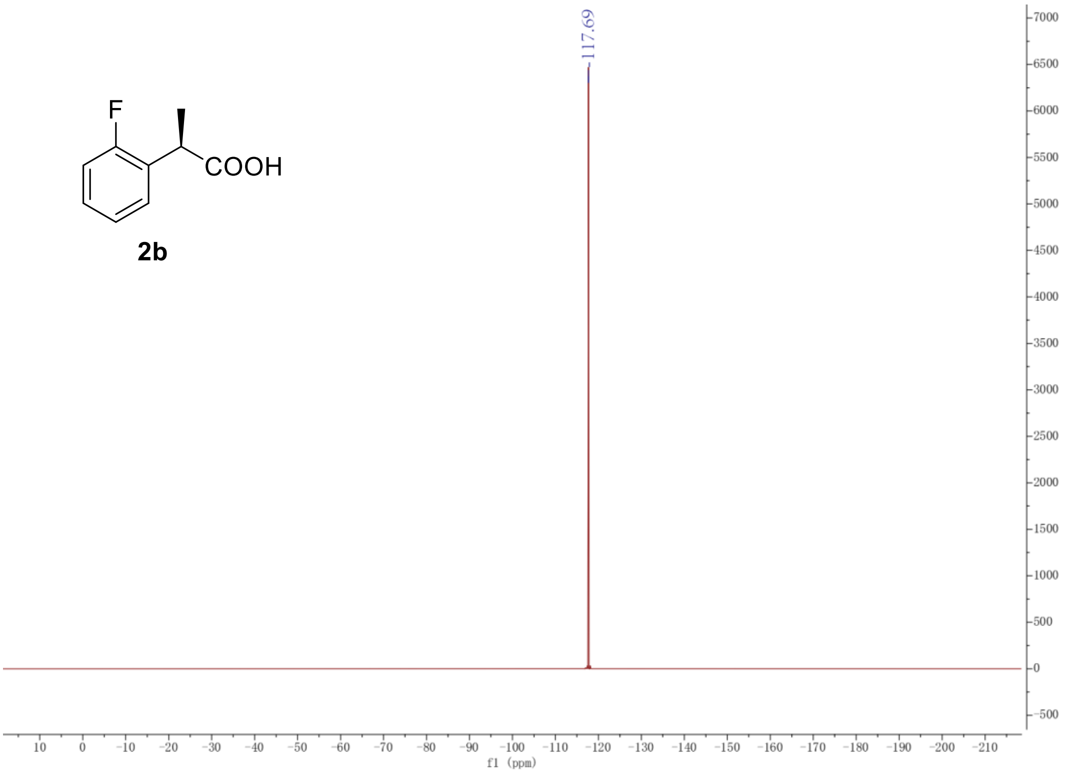

Supplementary Fig. 11 ^{1}H NMR (400 MHz), ^{13}C NMR (101 MHz) & ^{19}F NMR (376 MHz) spectra of compound **2b** in $CDCl_3$.

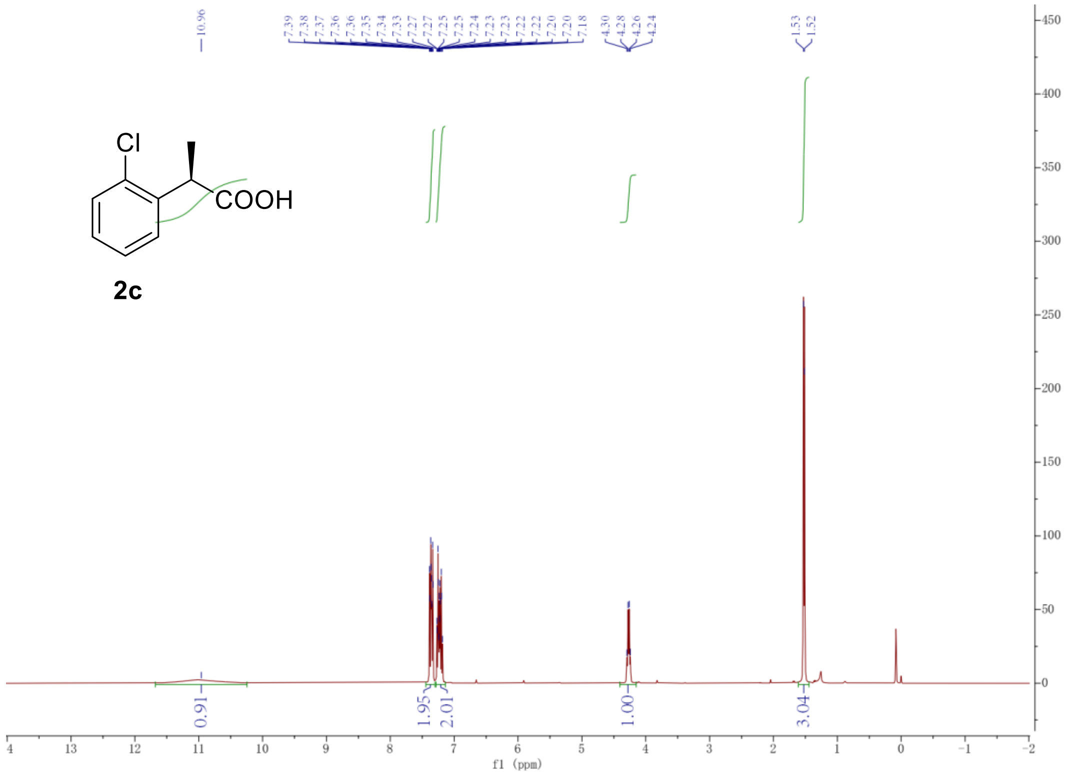

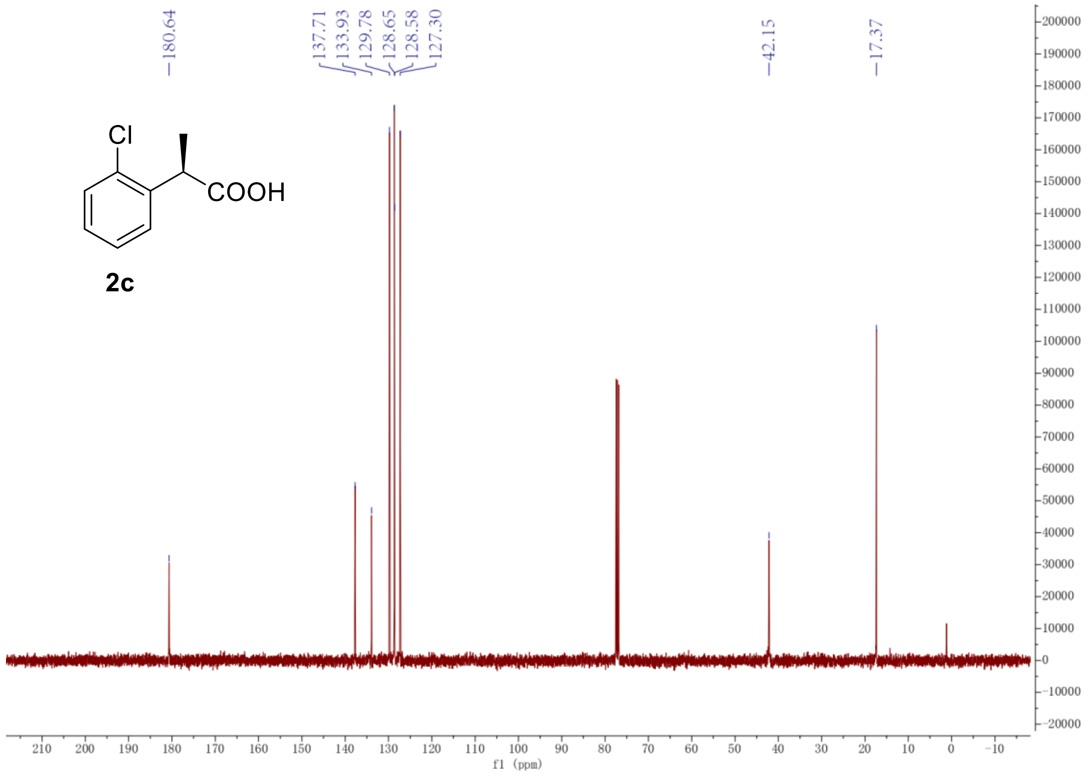

Supplementary Fig. 12 ^{1}H NMR (400 MHz) & ^{13}C NMR (101 MHz) spectra of compound **2c** in $CDCl_3$.

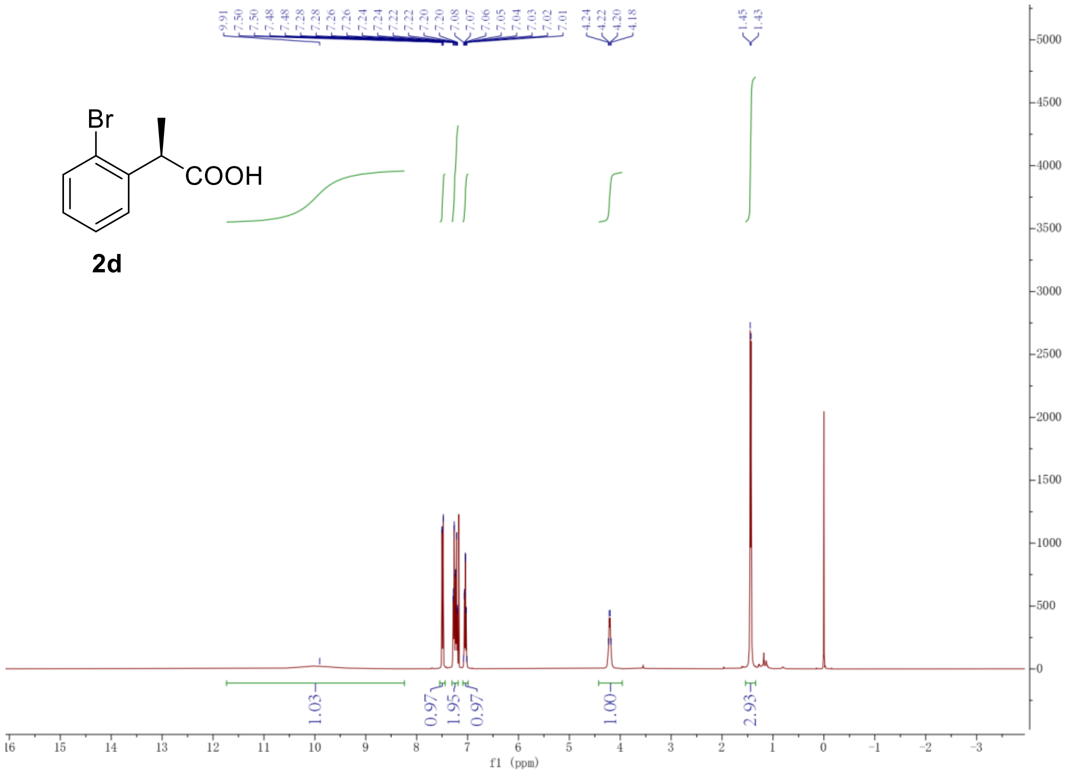

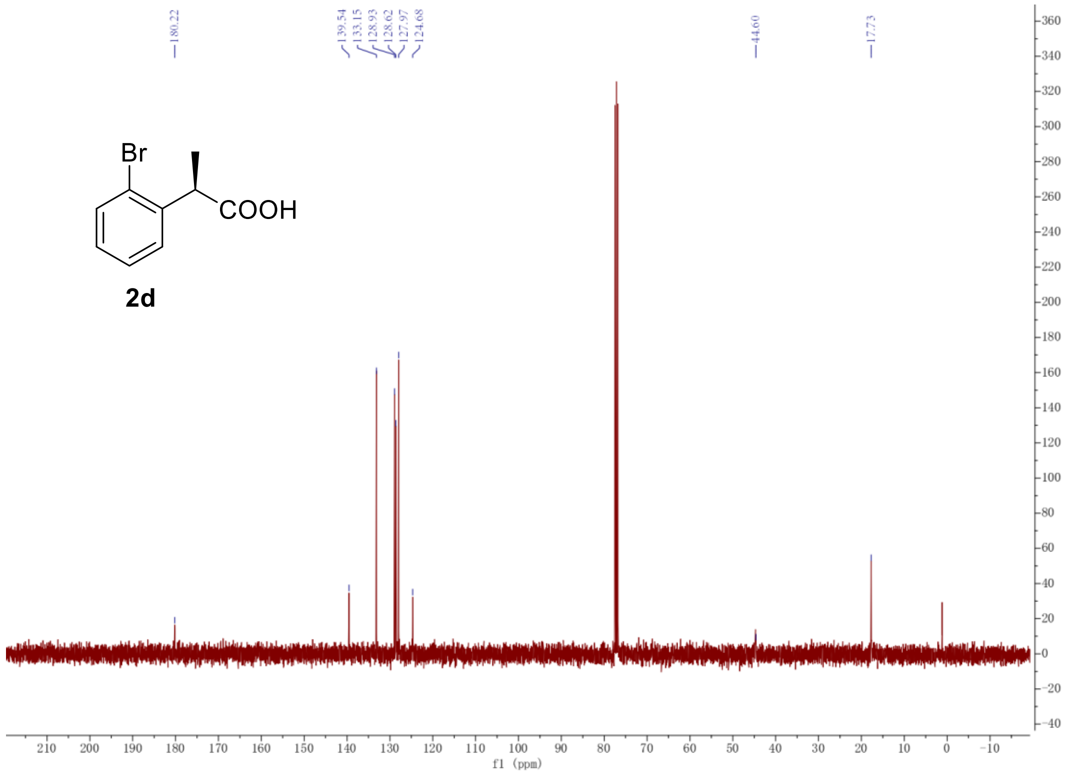

Supplementary Fig. 13 ^{1}H NMR (400 MHz) & ^{13}C NMR (101 MHz) spectra of compound **2d** in $CDCl_3$.

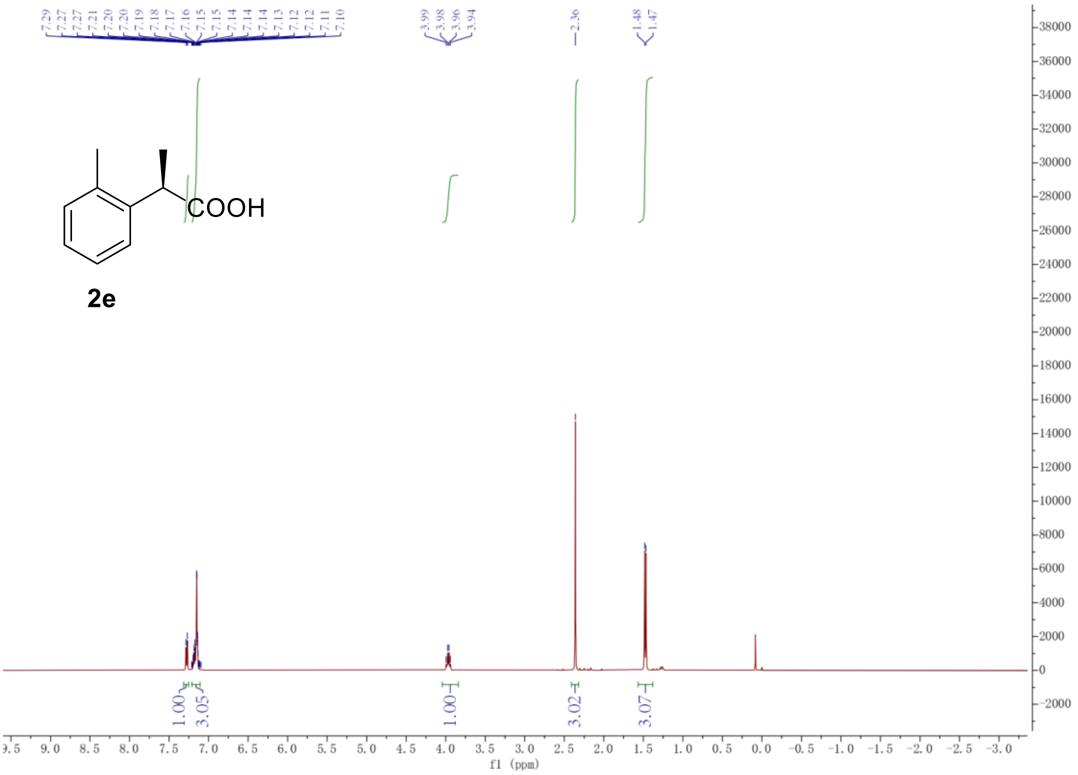

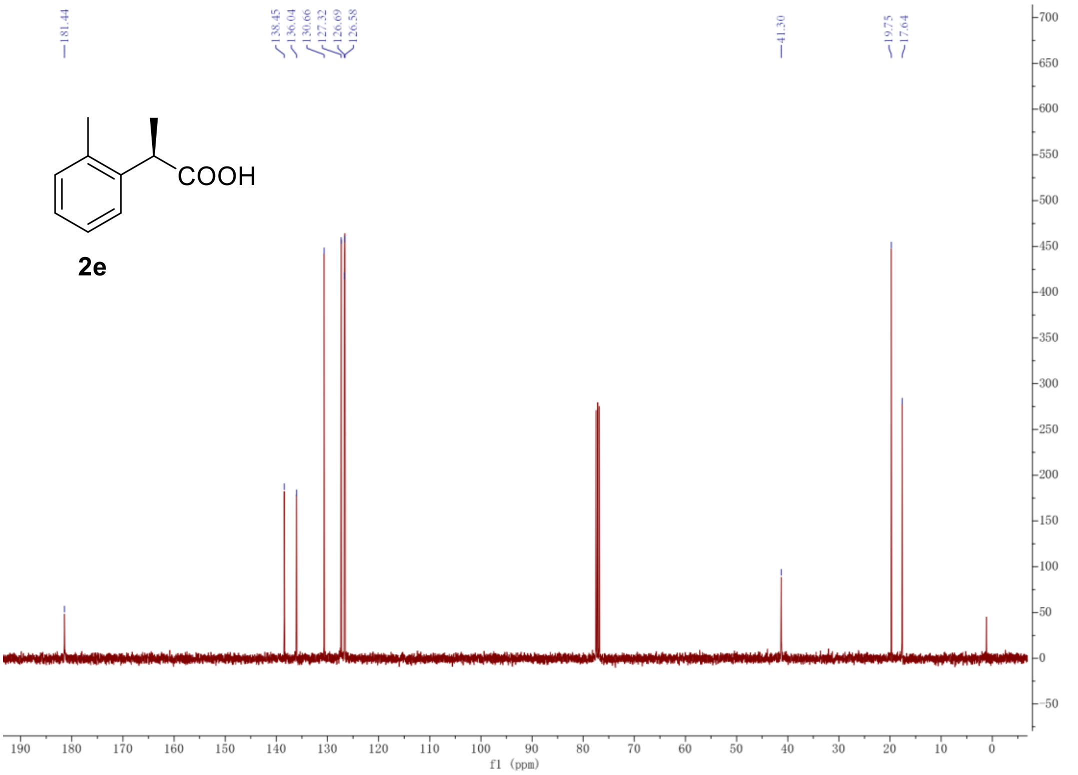

Supplementary Fig. 14 ^{1}H NMR (400 MHz) & ^{13}C NMR (101 MHz) spectra of compound **2e** in $CDCl_3$.

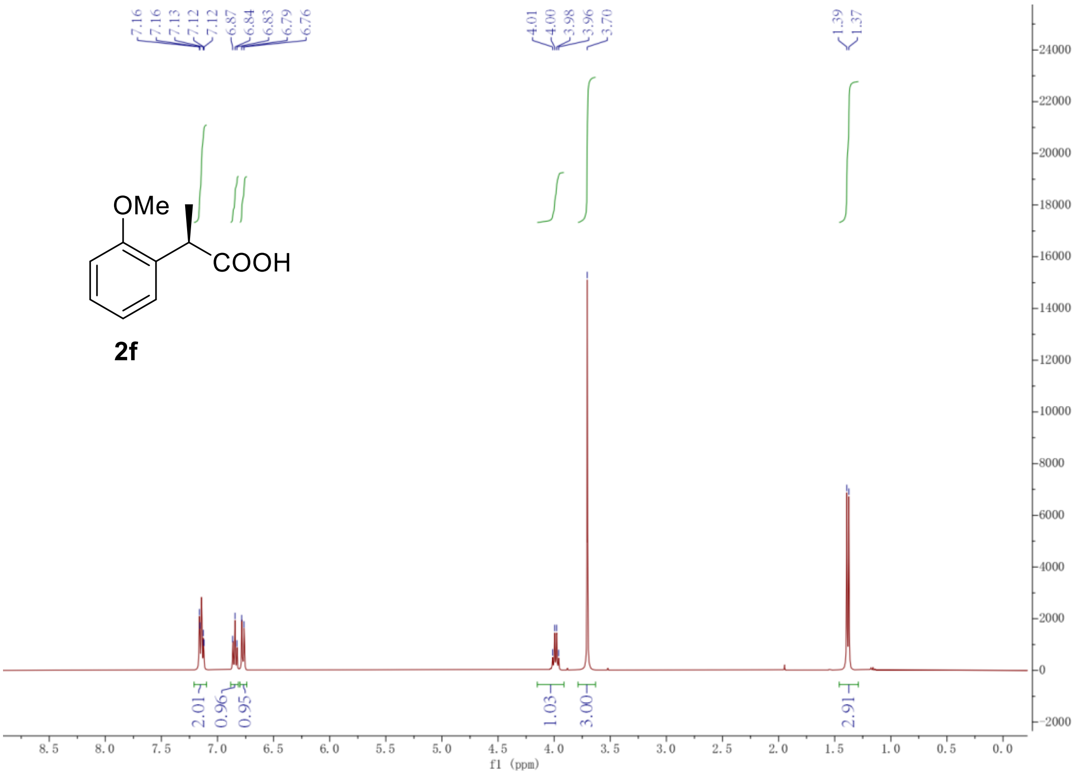

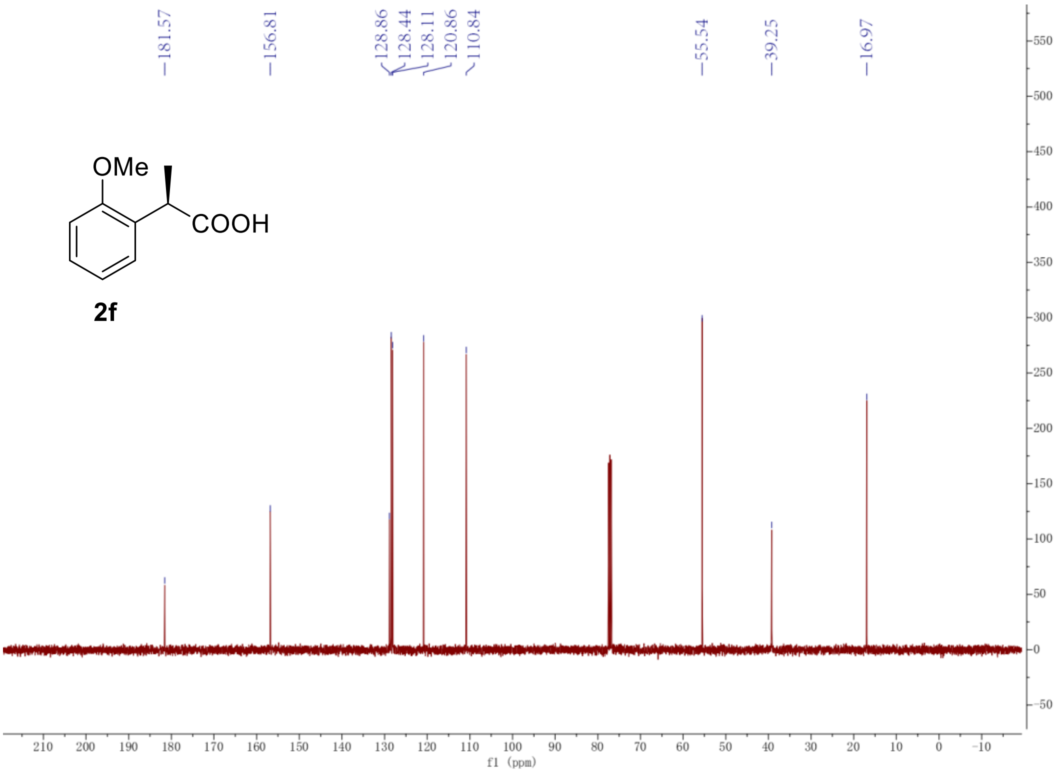

Supplementary Fig. 15 ^{1}H NMR (400 MHz) & ^{13}C NMR (101 MHz) spectra of compound **2f** in $CDCl_3$.

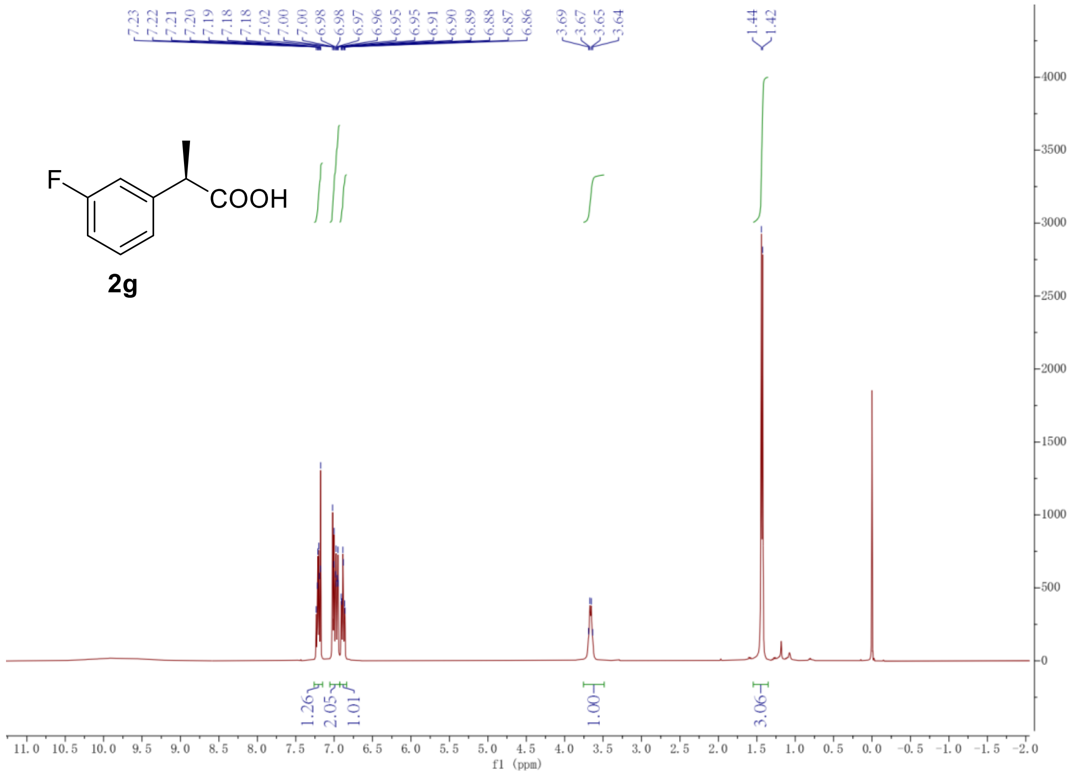
7.23
7.22
7.21
7.20
7.19
7.18
7.18
7.02
7.00
7.00
6.98
6.98
6.97
6.96
6.95
6.95
6.91
6.90
6.89
6.88
6.87
6.86
3.69
3.67
3.65
3.64
1.44
1.42
F
COOH
2g
1.26
2.05
1.01
1.00
3.06
f1 (ppm)

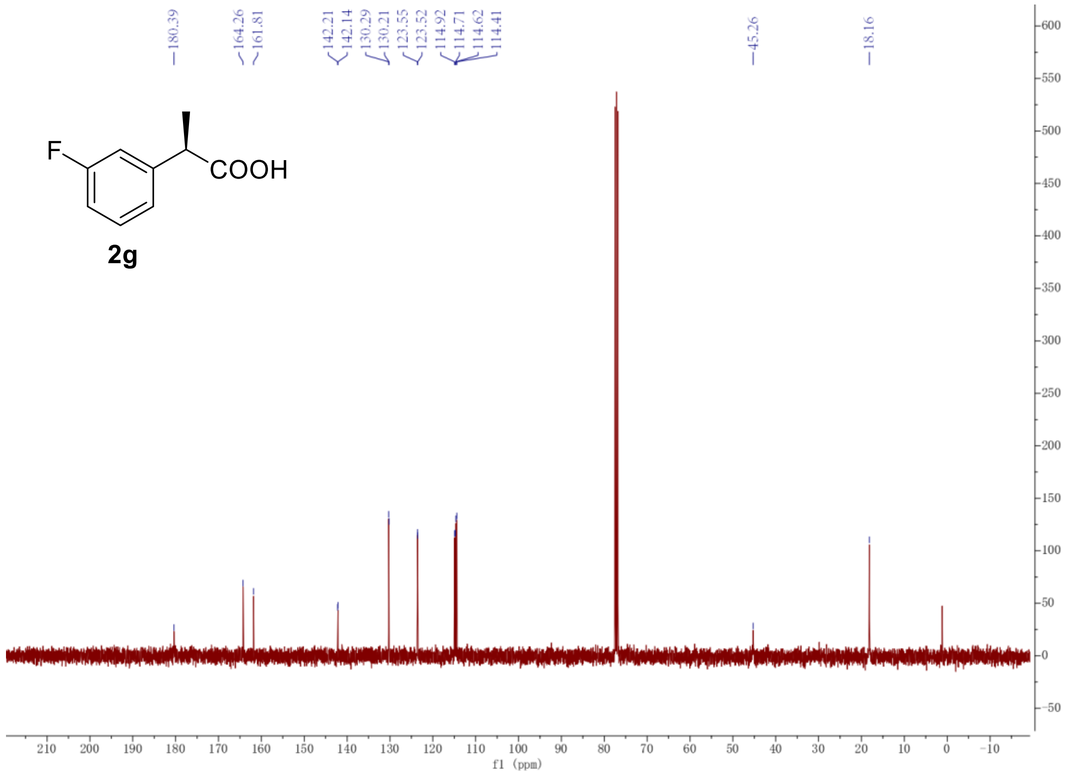
180.39
164.26
161.81
142.21
142.14
130.29
130.21
123.55
123.52
114.92
114.71
114.62
114.41
45.26
18.16
F
COOH
2g
f1 (ppm)

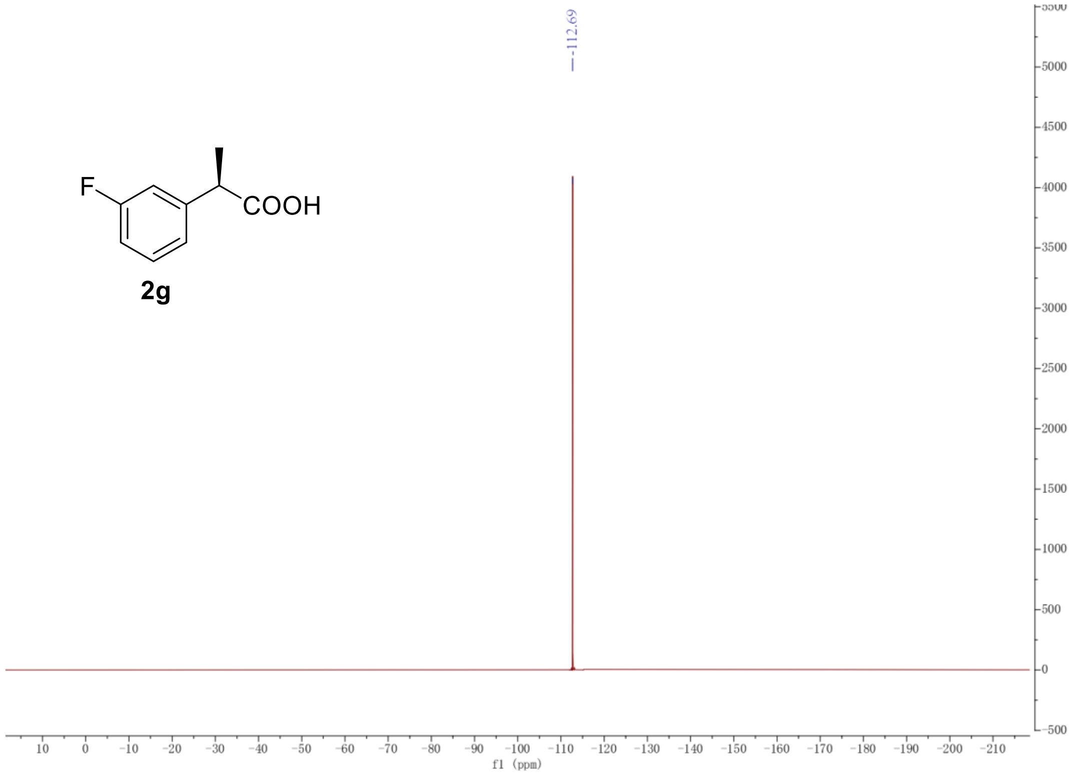

Supplementary Fig. 16 ^{1}H NMR (400 MHz), ^{13}C NMR (101 MHz) & ^{19}F NMR (376 MHz) spectra of compound **2g** in $CDCl_3$.

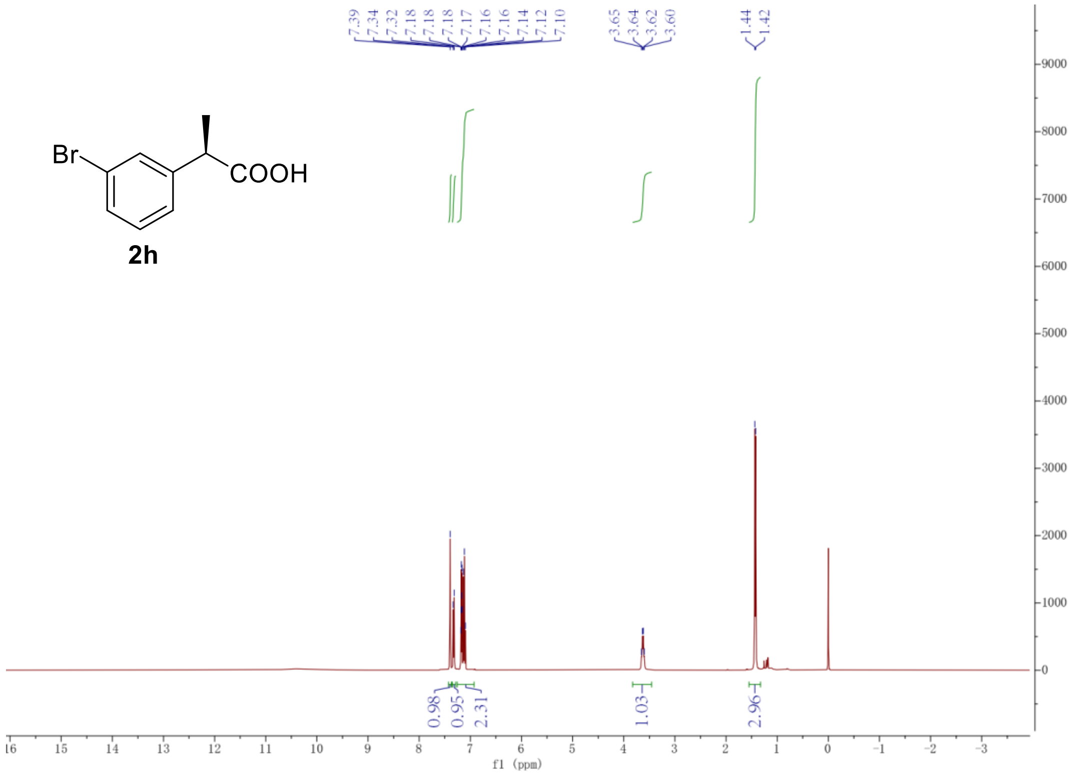

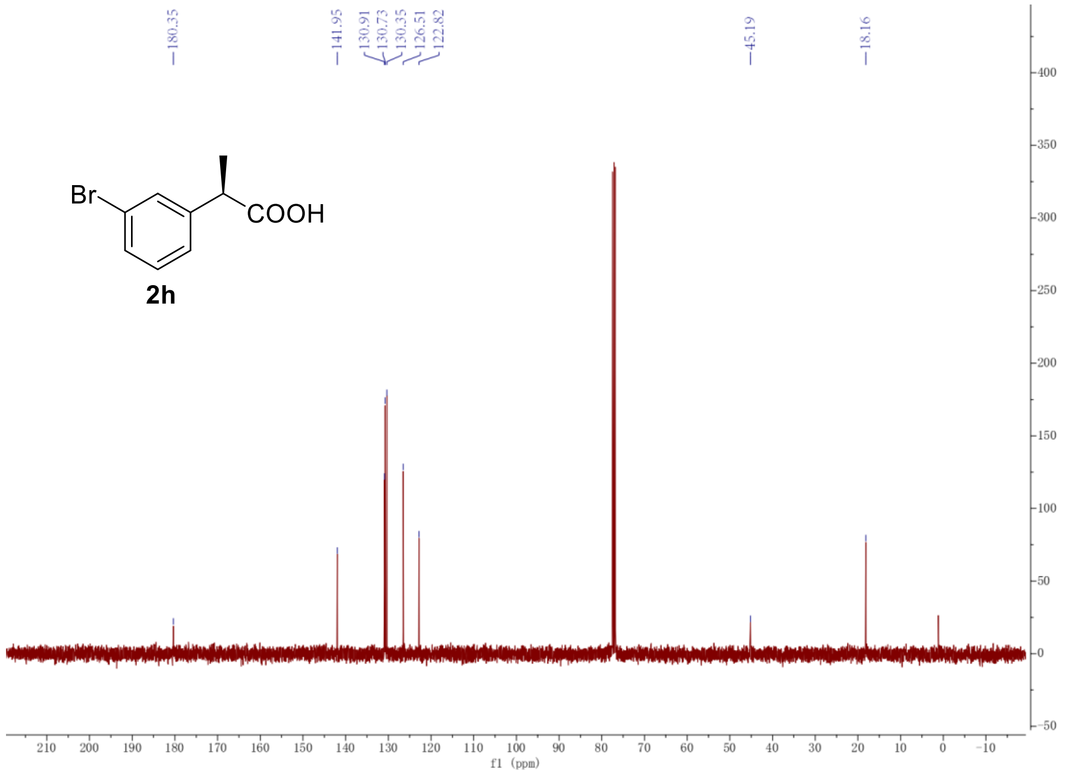

Supplementary Fig. 17 ^{1}H NMR (400 MHz) & ^{13}C NMR (101 MHz) spectra of compound **2h** in $CDCl_3$.

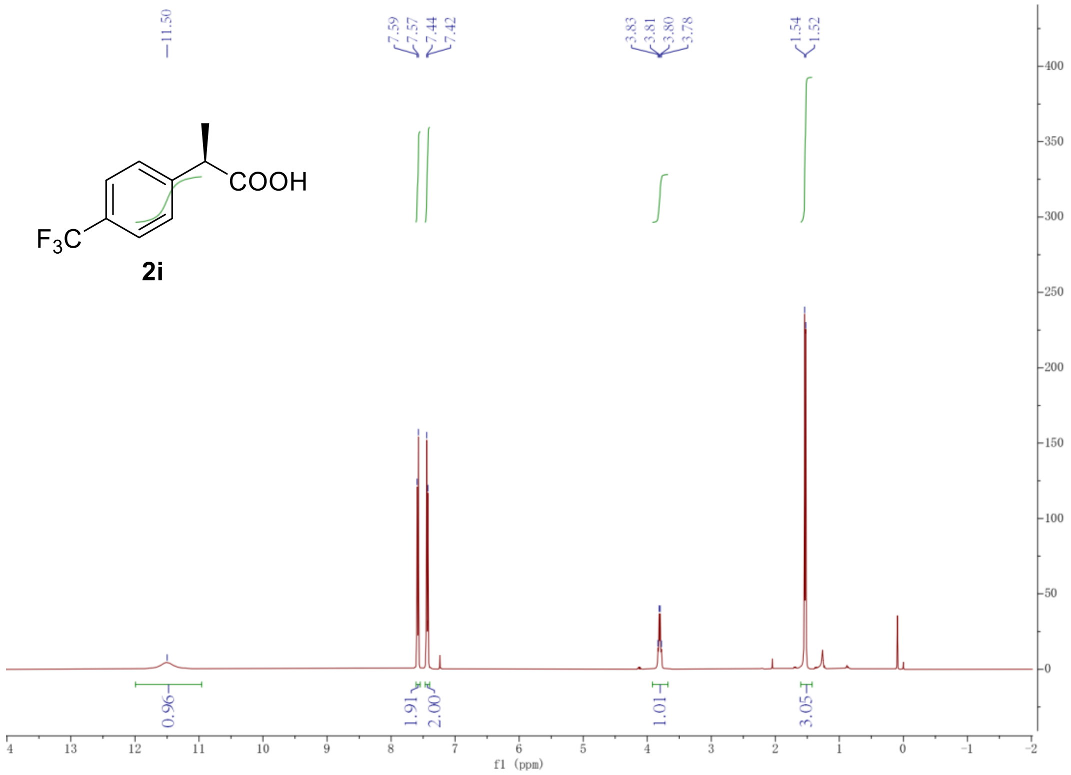

11.50
7.59
7.57
7.44
7.42
3.83
3.81
3.80
3.78
1.54
1.52
COOH
F3C
2i
0.96
1.91
2.00
1.01
3.05
f1 (ppm)

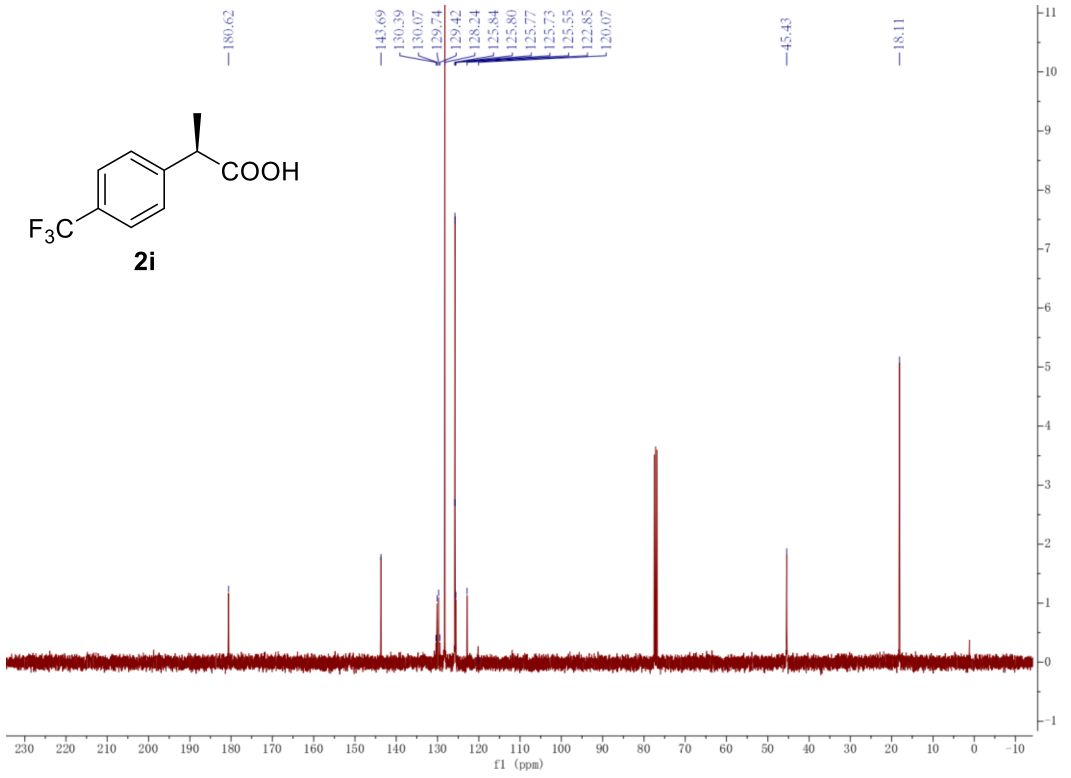

180.62
143.69
130.39
130.07
129.74
129.42
128.24
125.84
125.80
125.77
125.73
125.55
122.85
120.07
45.43
18.11
COOH
F3C
2i
f1 (ppm)

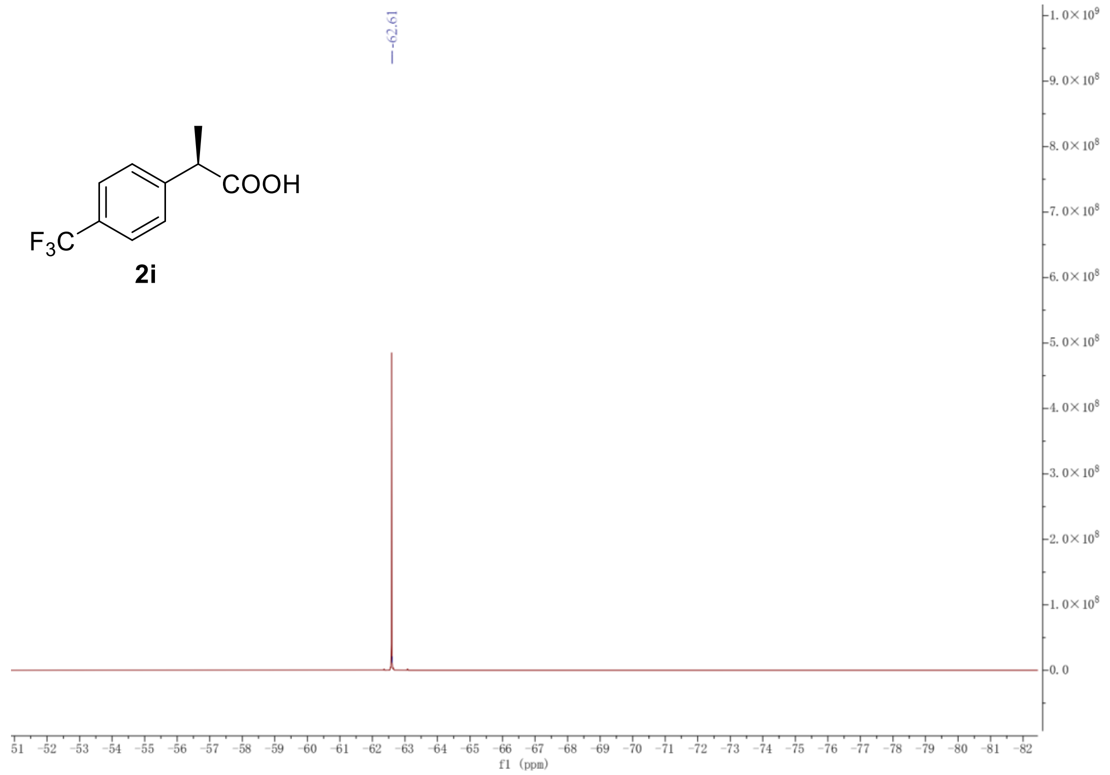

Supplementary Fig. 18 ^{1}H NMR (400 MHz), ^{13}C NMR (101 MHz) & ^{19}F NMR (376 MHz) spectra of compound **2i** in $CDCl_3$.

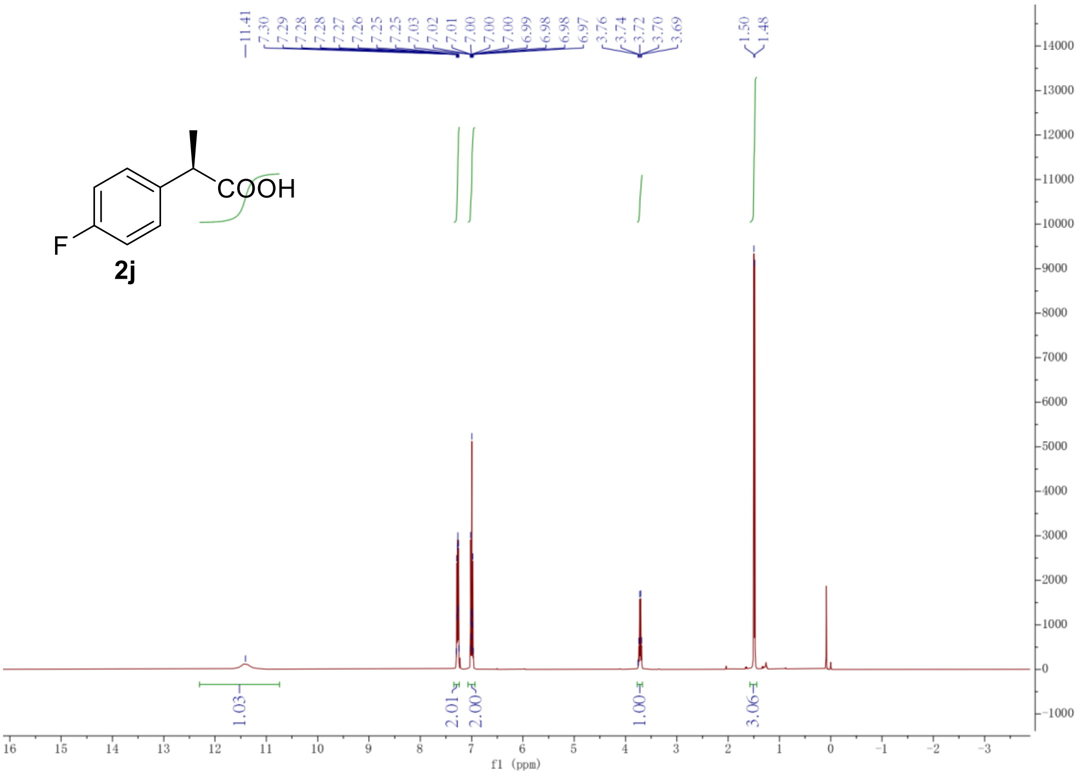
COOH
F
2j
11.41
7.30
7.29
7.28
7.28
7.27
7.26
7.25
7.25
7.03
7.02
7.01
7.00
7.00
7.00
6.99
6.98
6.98
6.97
3.76
3.74
3.72
3.70
3.69
1.50
1.48
1.03
2.01
2.00
1.00
3.06
f1 (ppm)

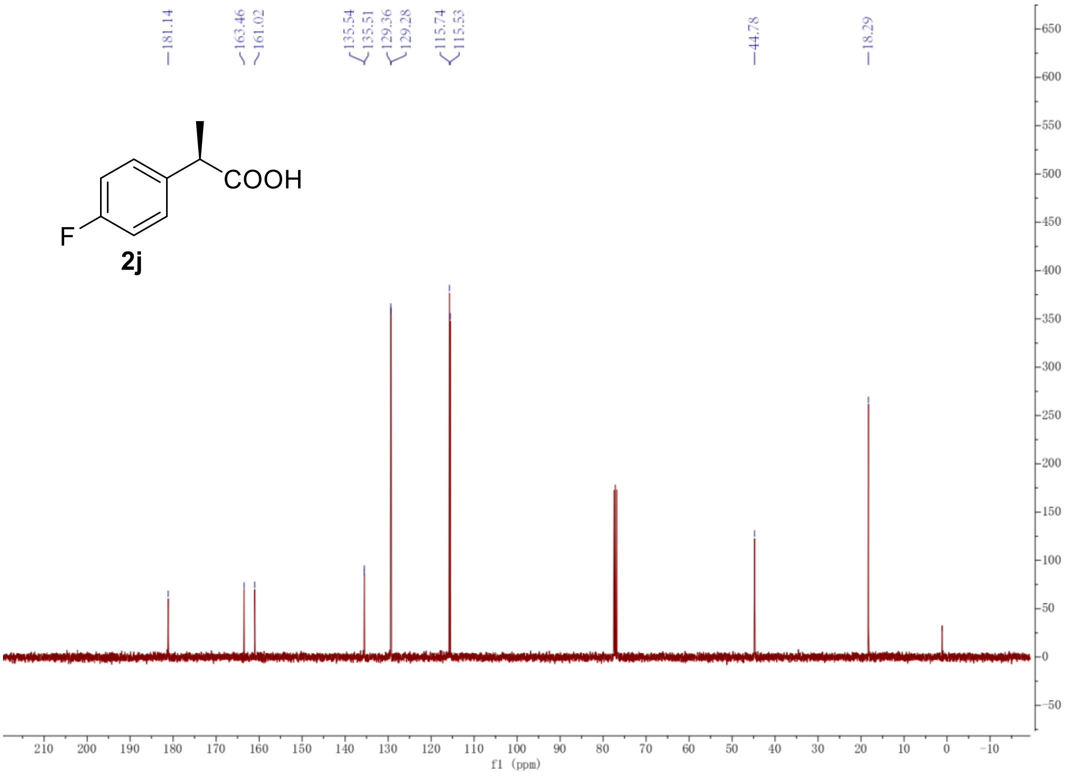
COOH
F
2j
181.14
163.46
161.02
135.54
135.51
129.36
129.28
115.74
115.53
44.78
18.29
f1 (ppm)

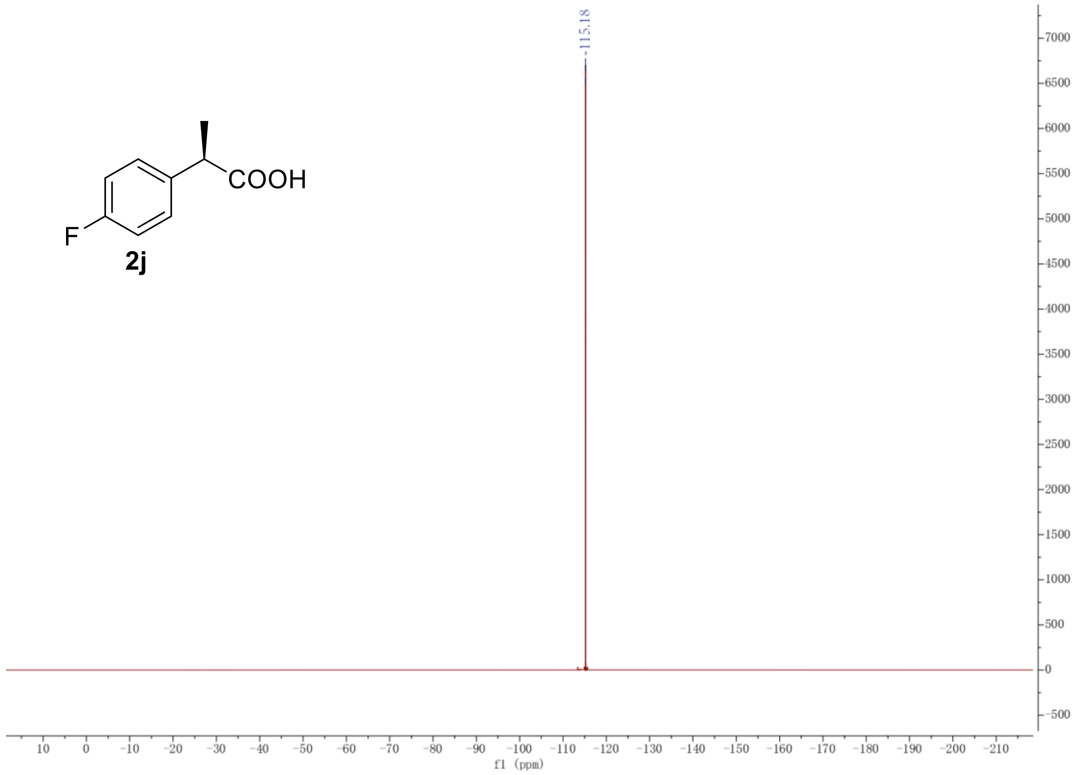

Supplementary Fig. 19 ^{1}H NMR (400 MHz), ^{13}C NMR (101 MHz) & ^{19}F NMR (376 MHz) spectra of compound **2j** in $CDCl_3$.

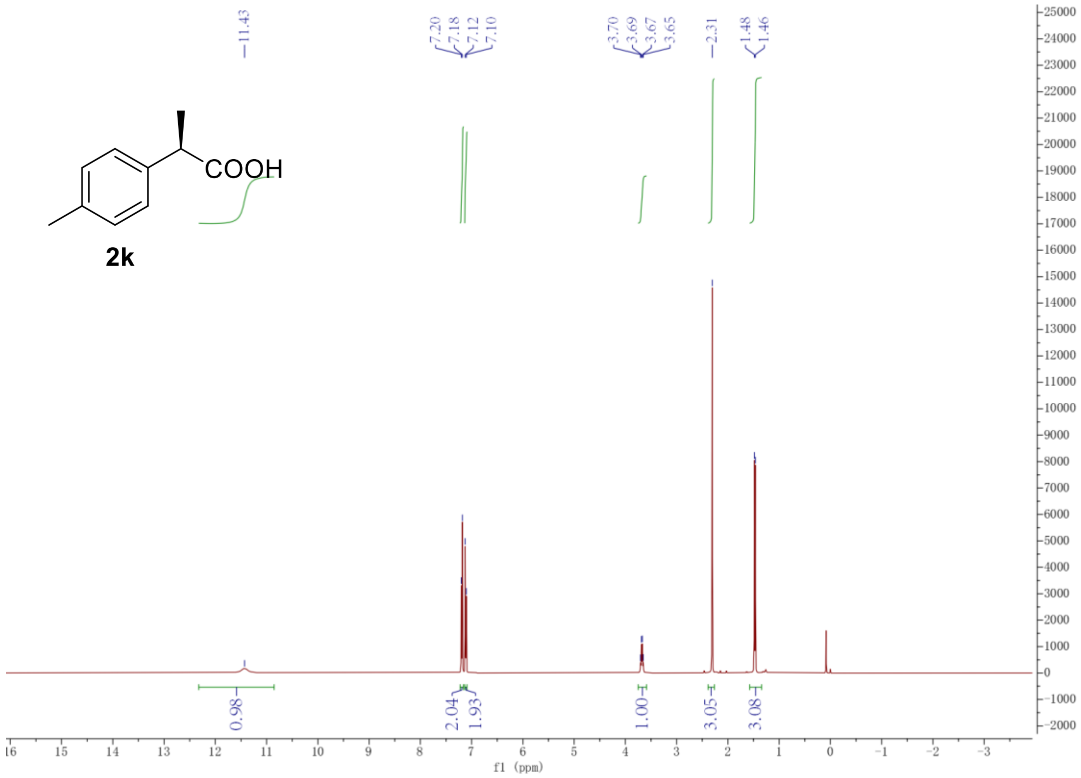

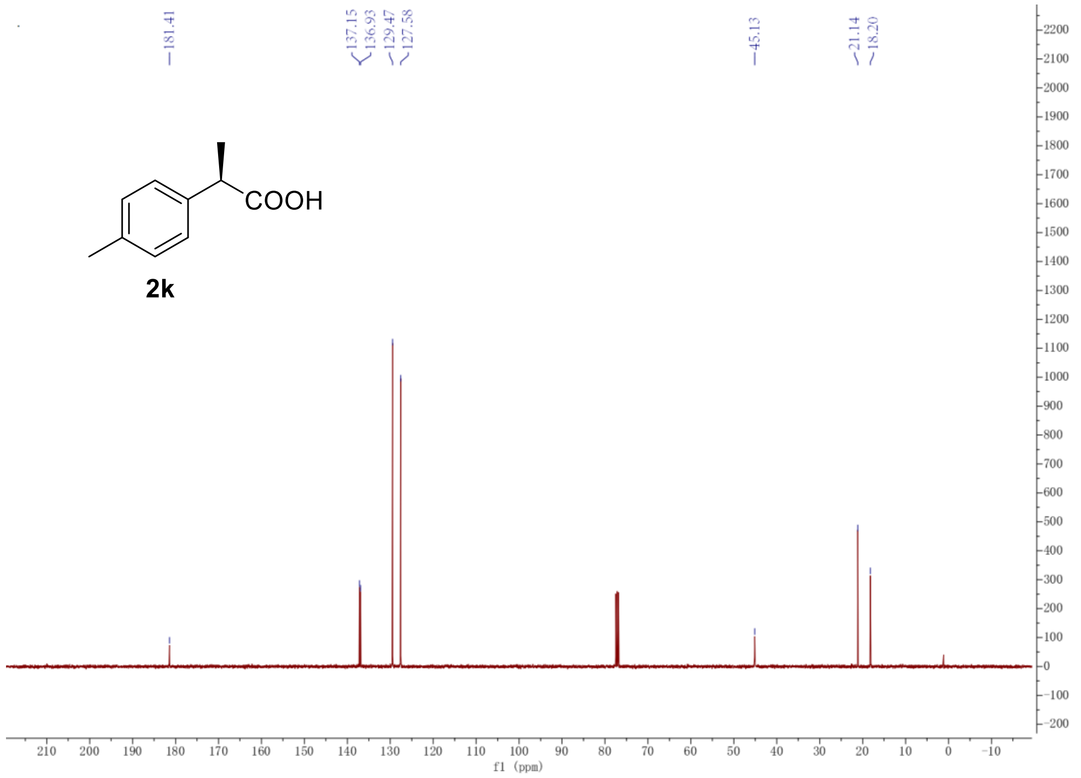

Supplementary Fig. 20 ^{1}H NMR (400 MHz) & ^{13}C NMR (101 MHz) spectra of compound **2k** in $CDCl_3$.

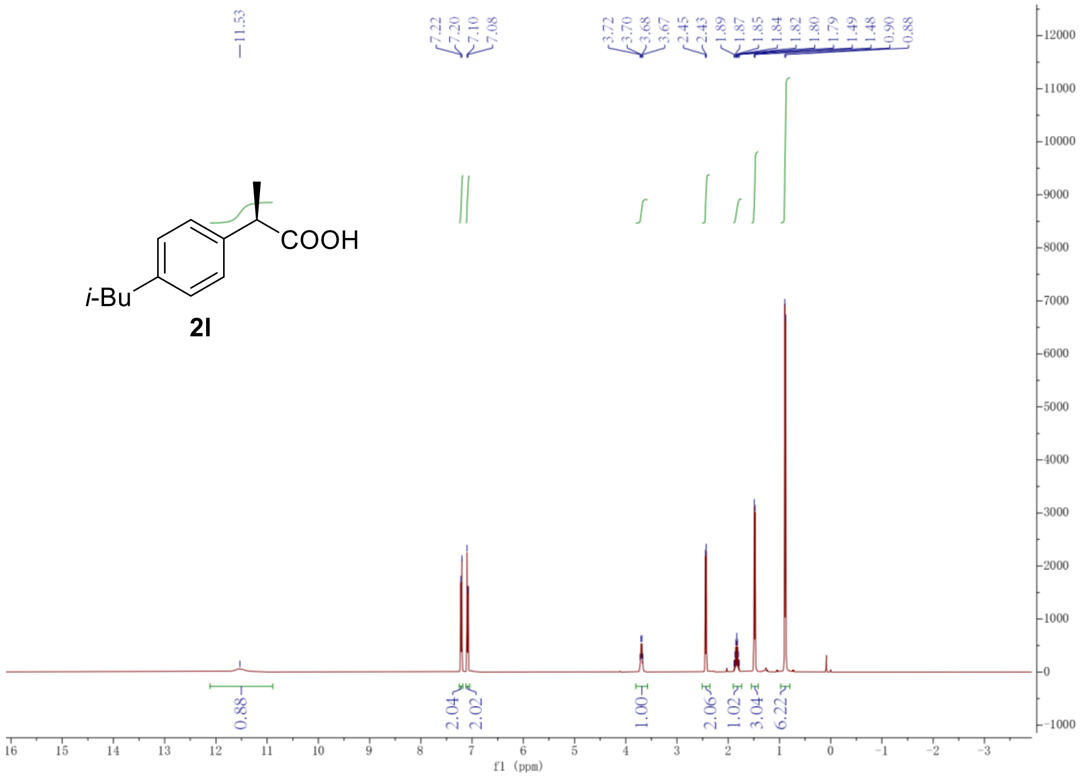

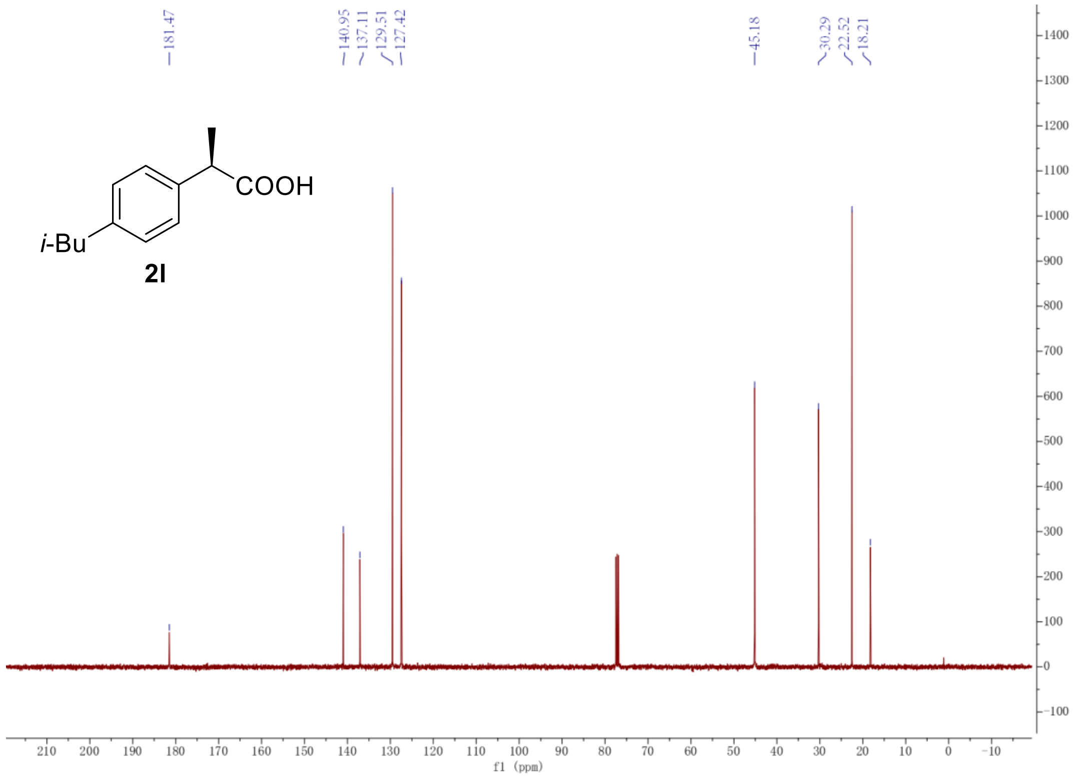

Supplementary Fig. 21 ^{1}H NMR (400 MHz) & ^{13}C NMR (101 MHz) spectra of compound **2l** in $CDCl_3$.

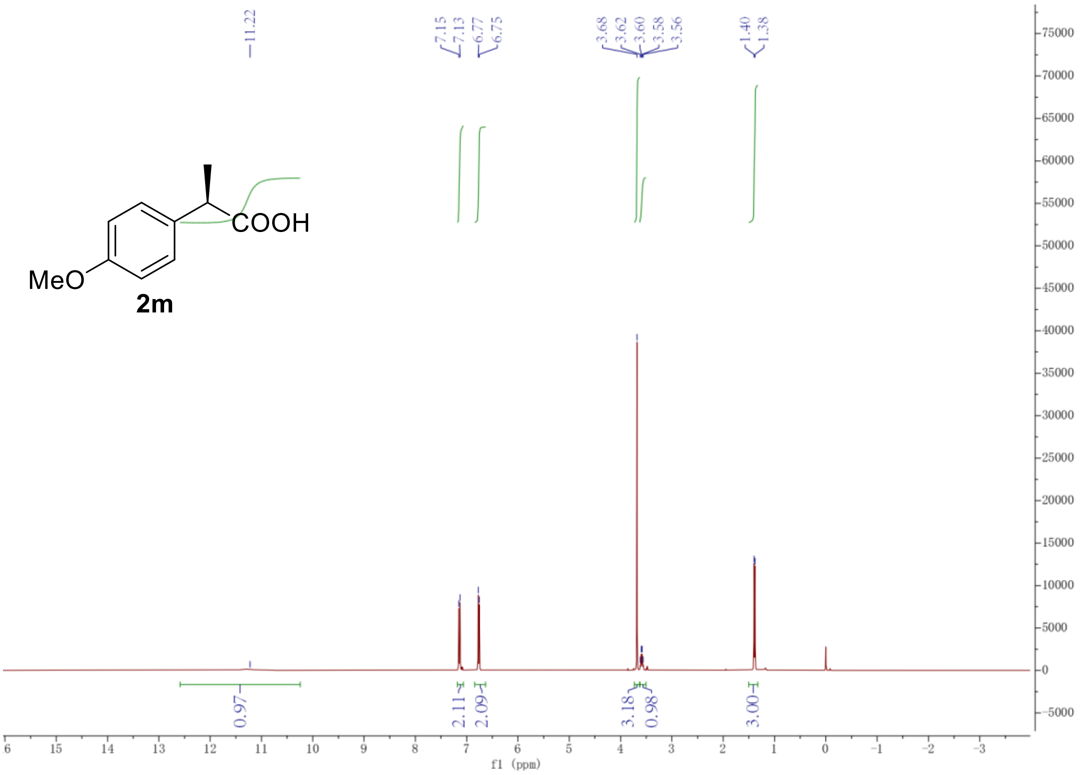

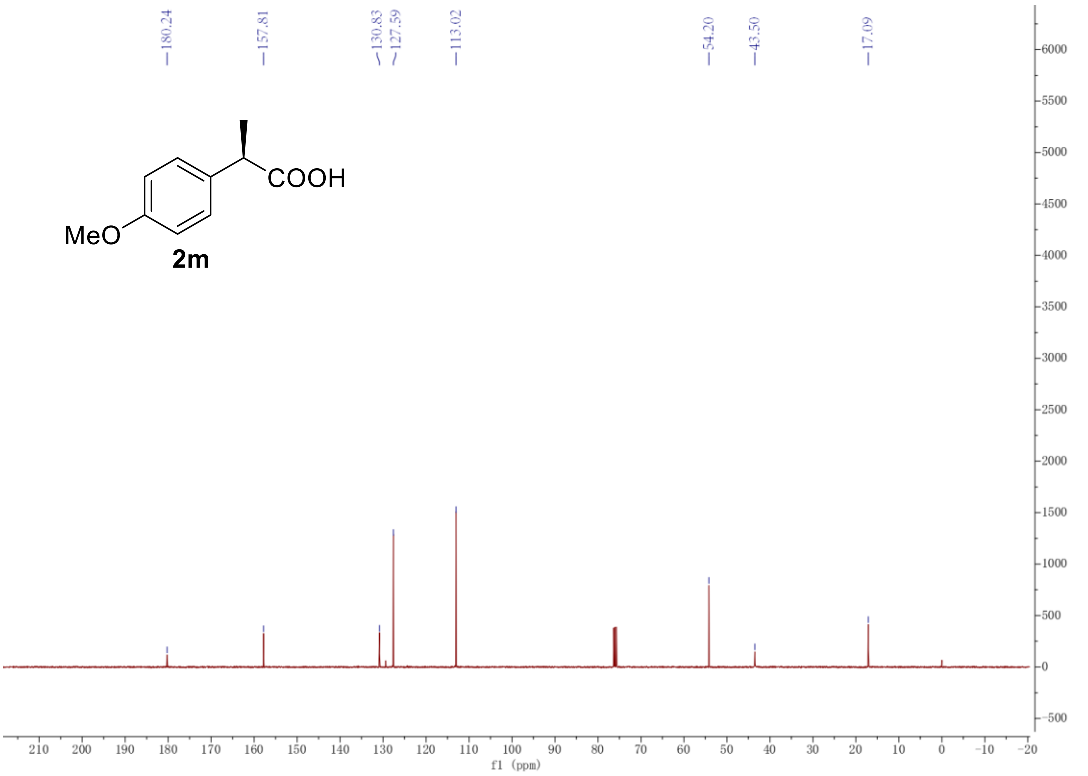

Supplementary Fig. 22 ^{1}H NMR (400 MHz) & ^{13}C NMR (101 MHz) spectra of compound **2m** in $CDCl_3$.

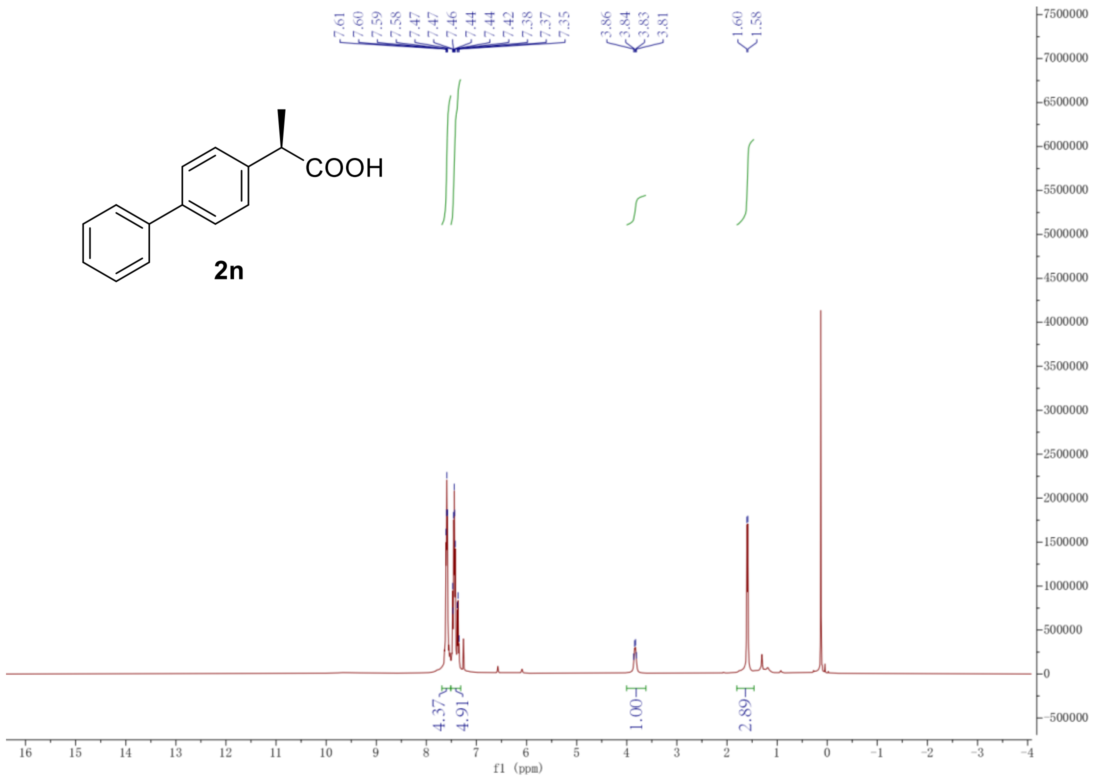

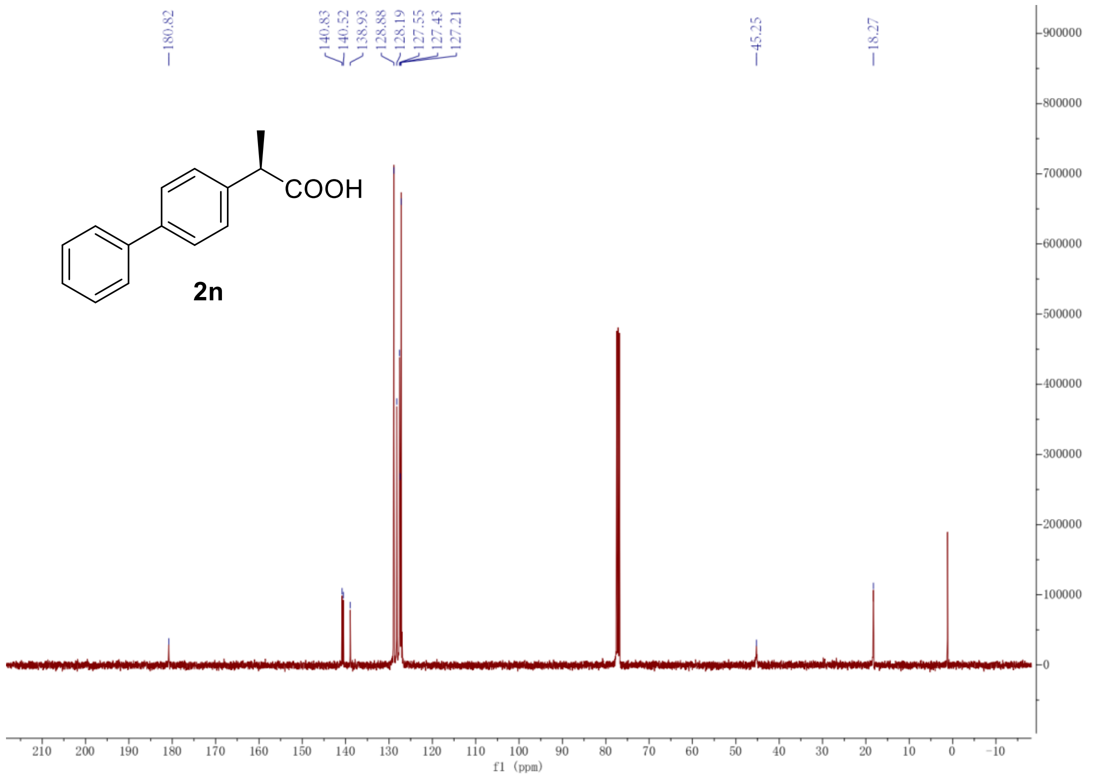

Supplementary Fig. 23 ^{1}H NMR (400 MHz) & ^{13}C NMR (101 MHz) spectra of compound **2n** in $CDCl_3$.

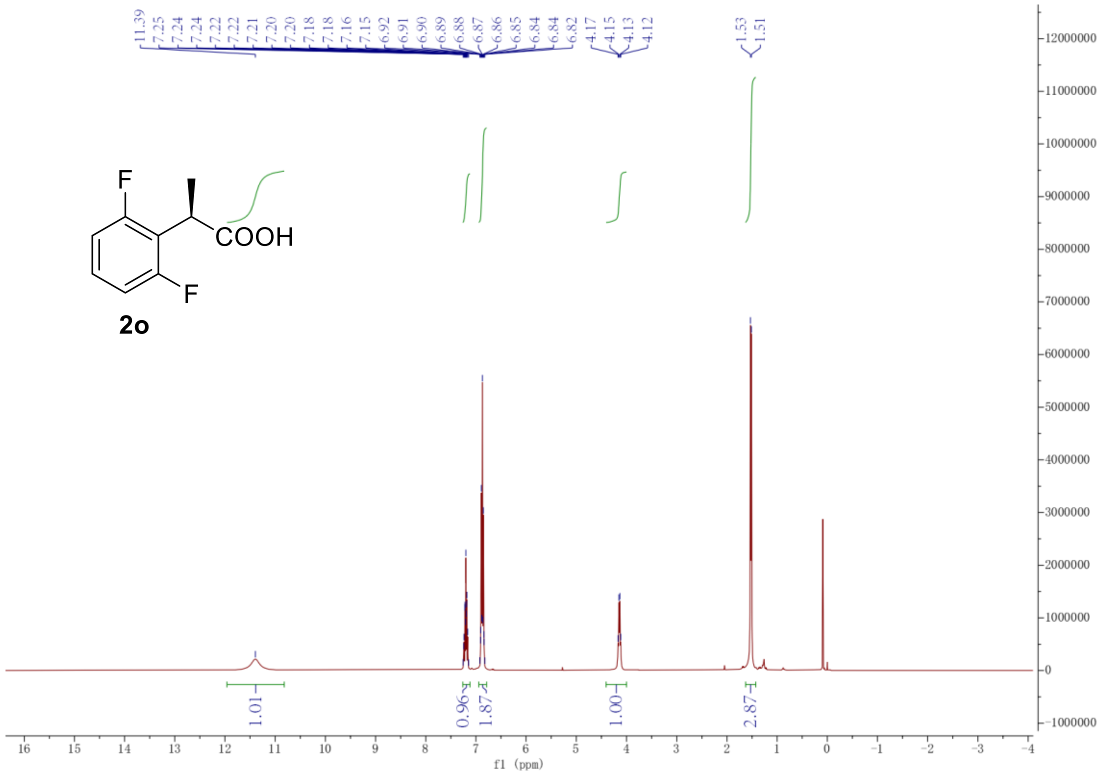
11.39
7.25
7.24
7.24
7.22
7.22
7.21
7.20
7.20
7.18
7.18
7.16
7.15
6.92
6.91
6.90
6.89
6.88
6.87
6.86
6.85
6.84
6.84
6.82
4.17
4.15
4.13
4.12
1.53
1.51
F
COOH
F
2o
1.01
0.96
1.87
1.00
2.87
f1 (ppm)

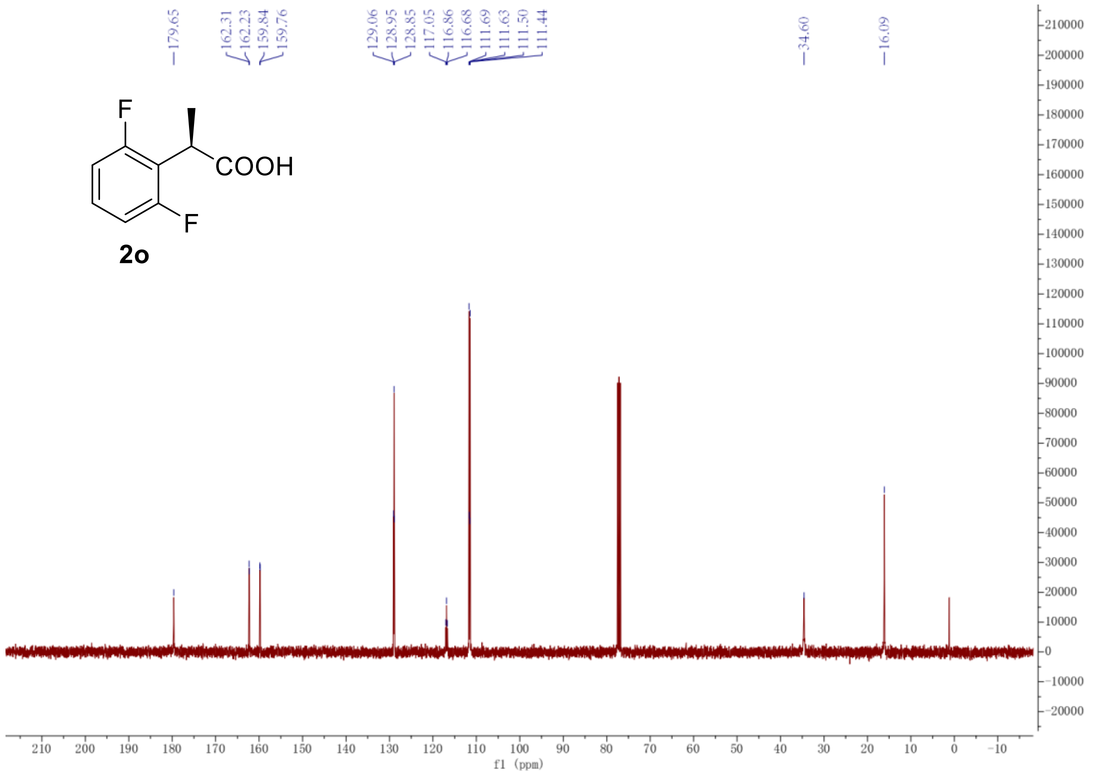
179.65
162.31
162.23
159.84
159.76
129.06
128.95
128.85
117.05
116.86
116.68
111.69
111.63
111.50
111.44
34.60
16.09
F
COOH
F
2o
f1 (ppm)

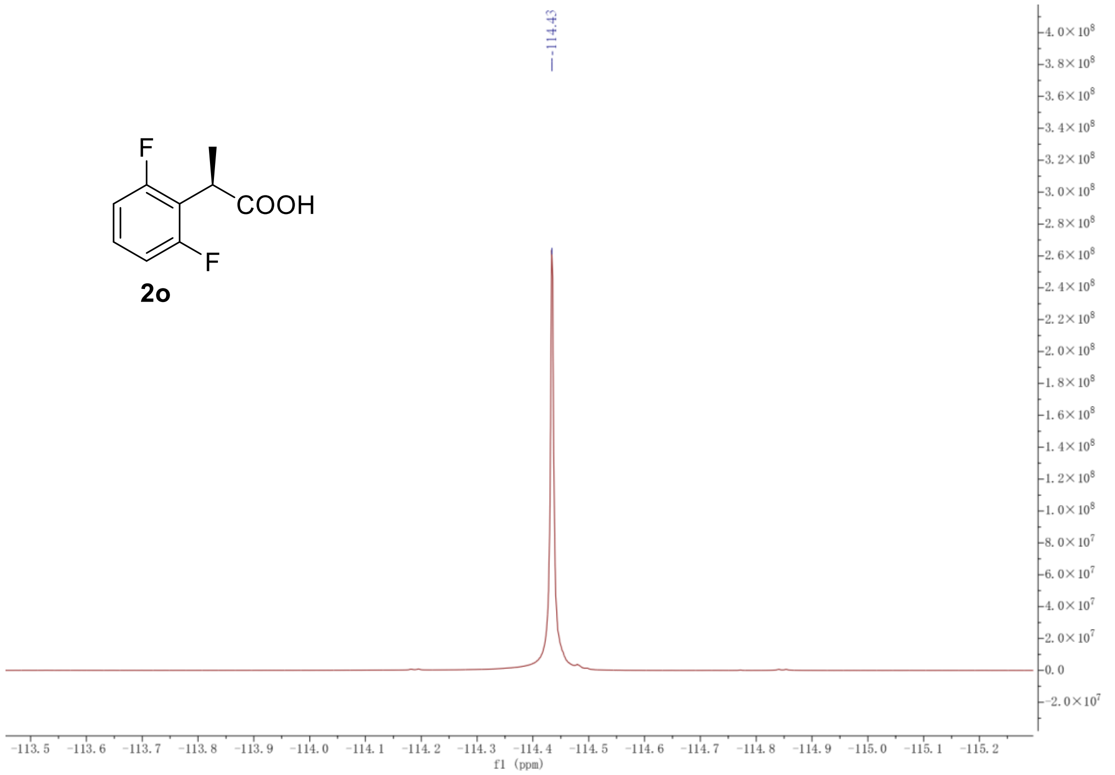

Supplementary Fig. 24 ^{1}H NMR (400 MHz), ^{13}C NMR (101 MHz) & ^{19}F NMR (376 MHz) spectra of compound **2o** in $CDCl_3$.

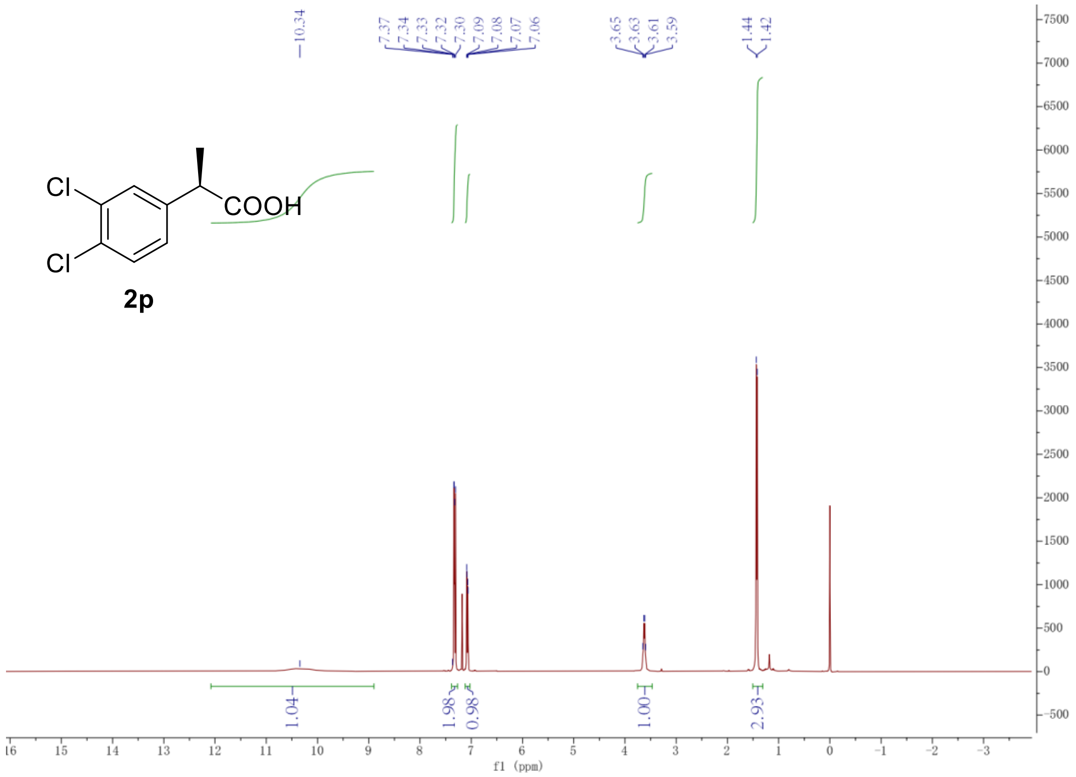

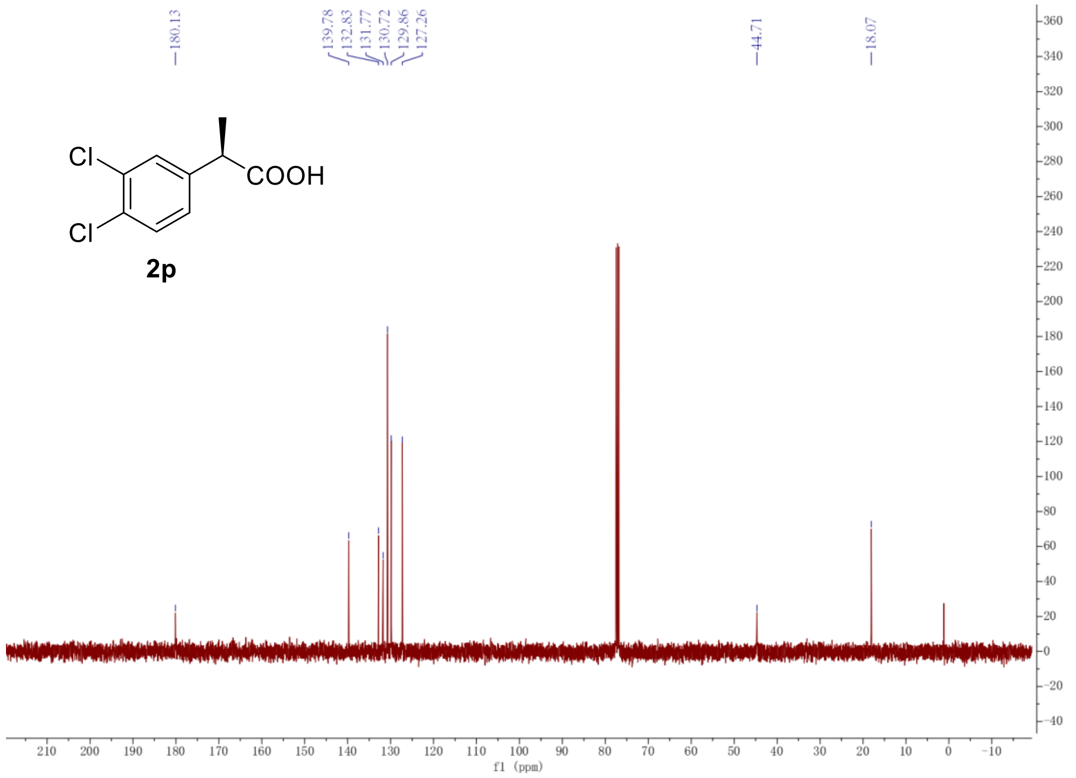

Supplementary Fig. 25 ^{1}H NMR (400 MHz) & ^{13}C NMR (101 MHz) spectra of compound **2p** in $CDCl_3$.

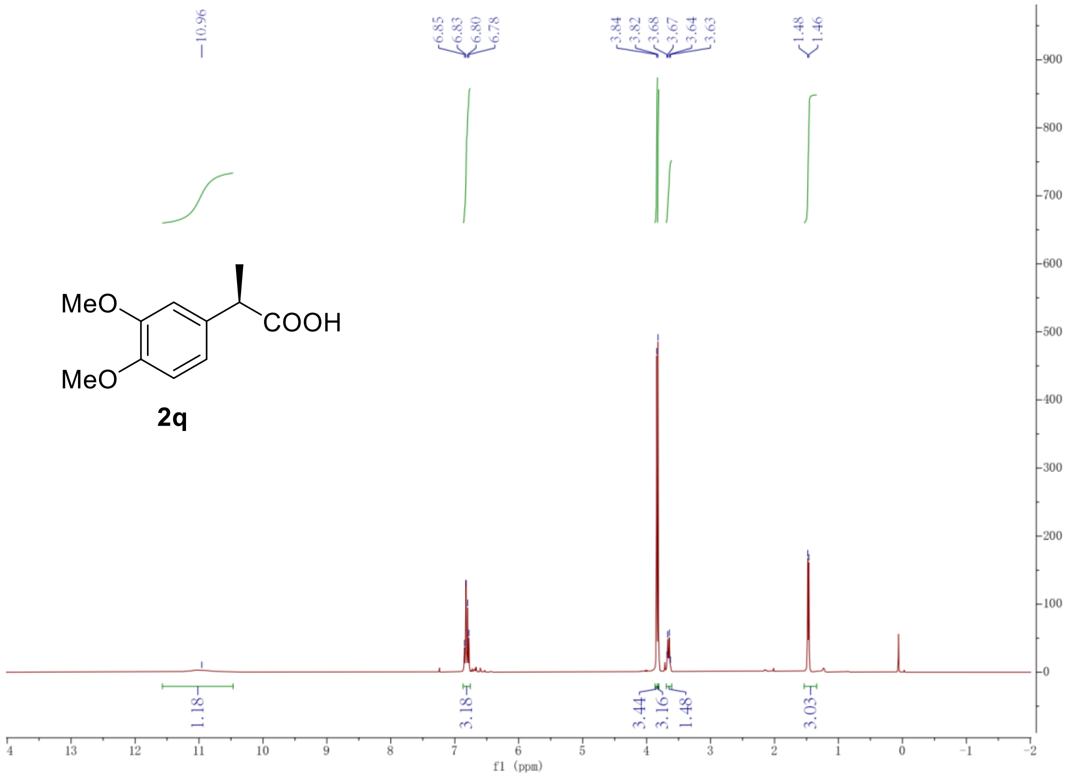

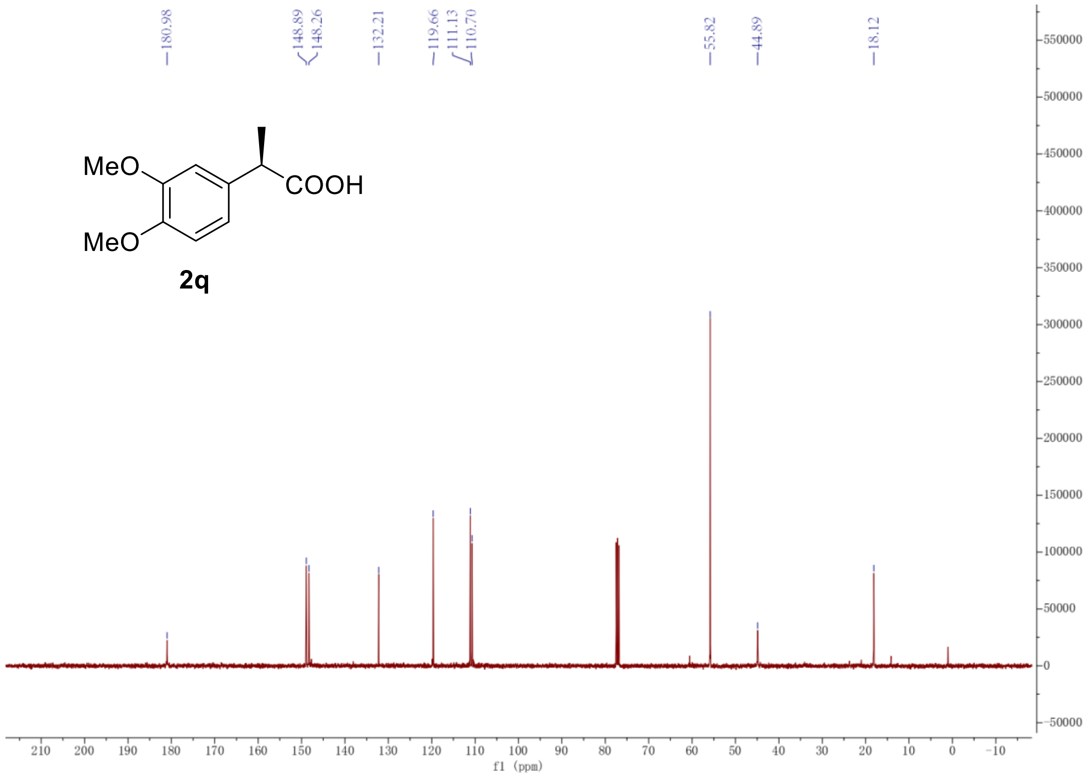

Supplementary Fig. 26 ^{1}H NMR (400 MHz) & ^{13}C NMR (101 MHz) spectra of compound **2q** in $CDCl_3$.

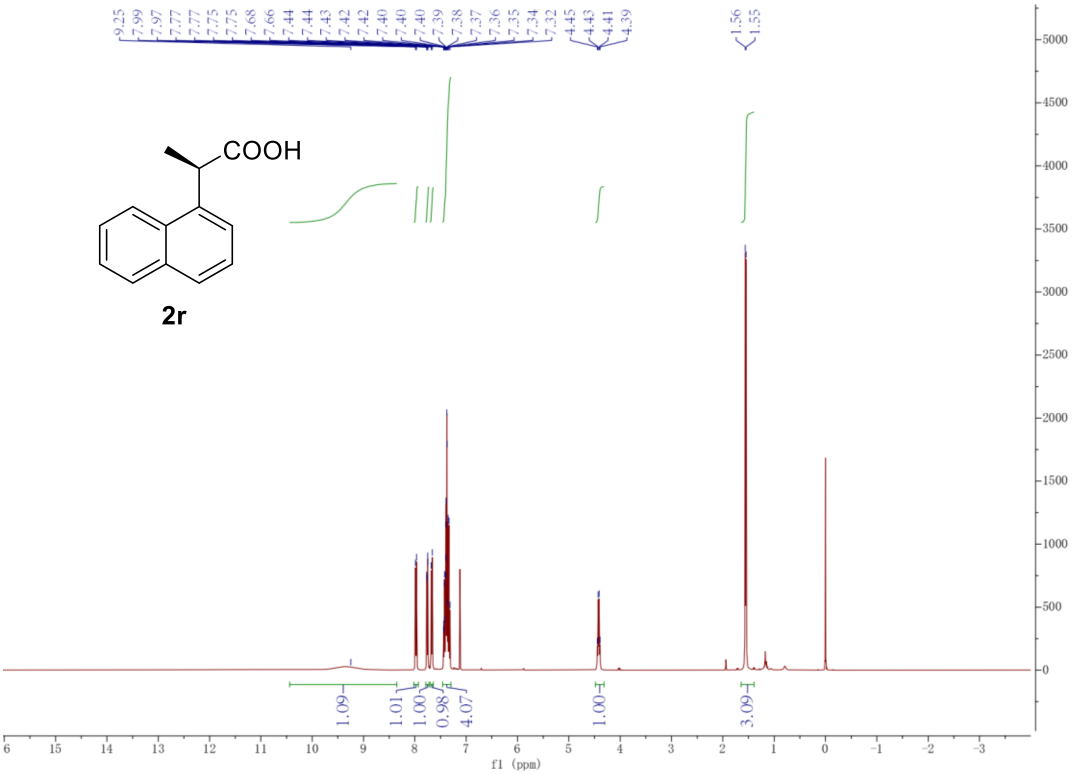

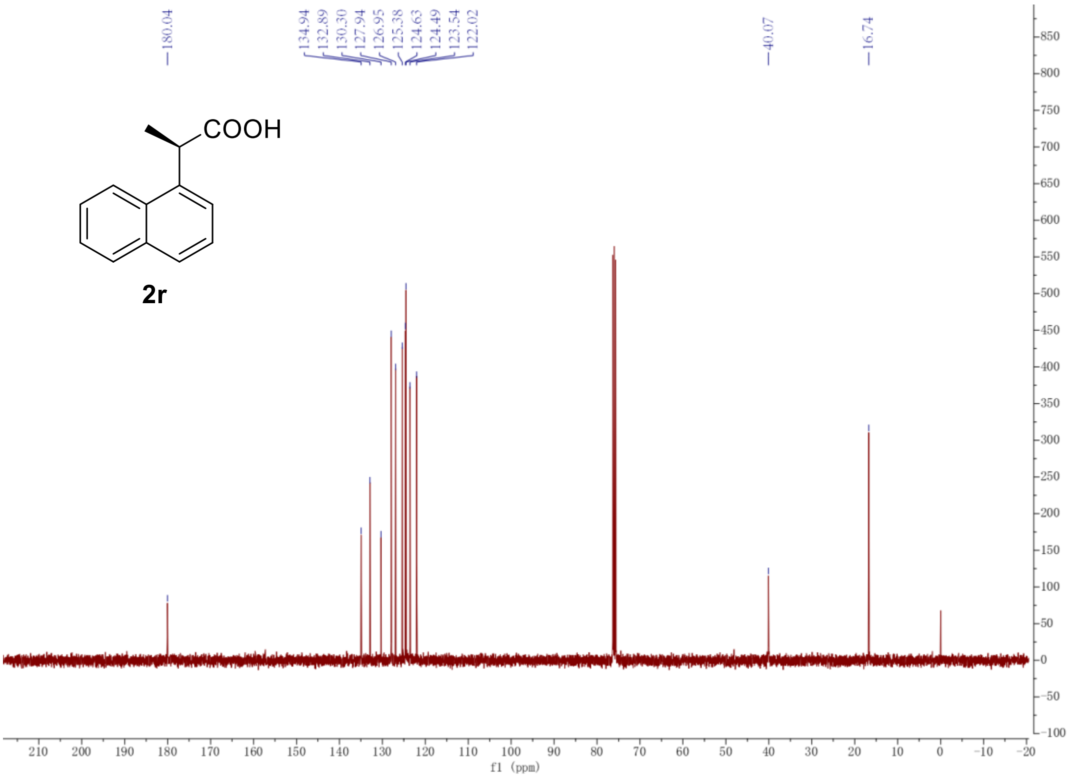

Supplementary Fig. 27 ^{1}H NMR (400 MHz) & ^{13}C NMR (101 MHz) spectra of compound **2r** in $CDCl_3$.

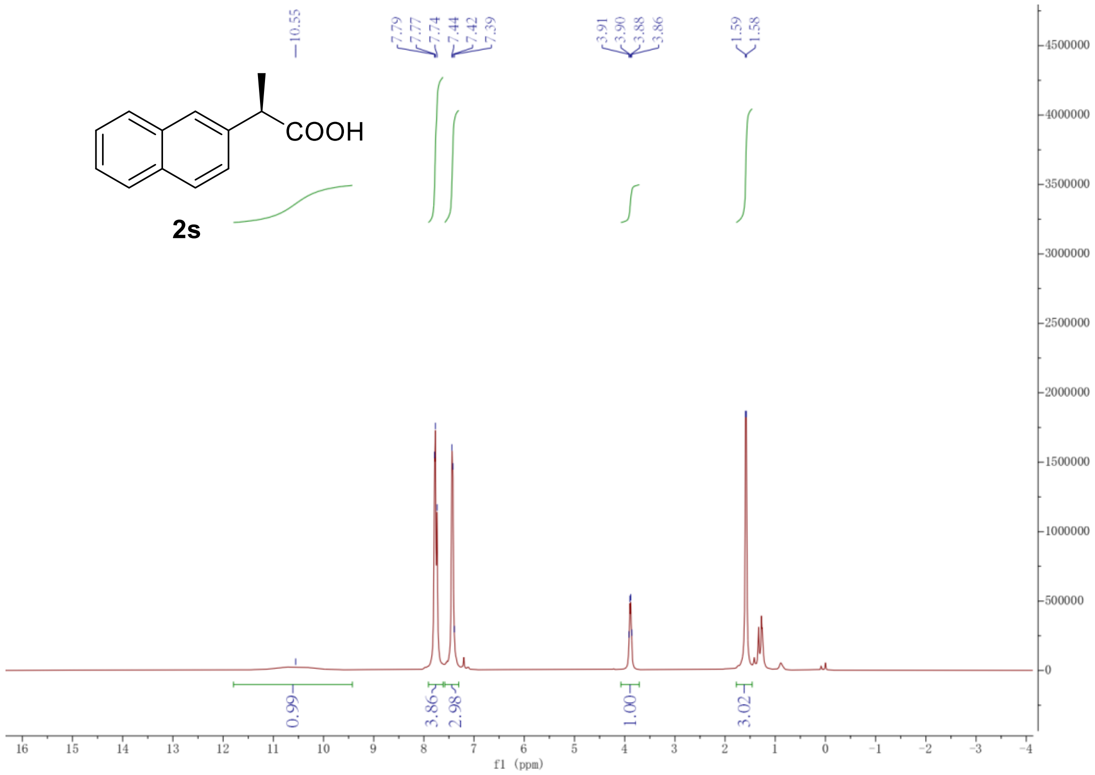

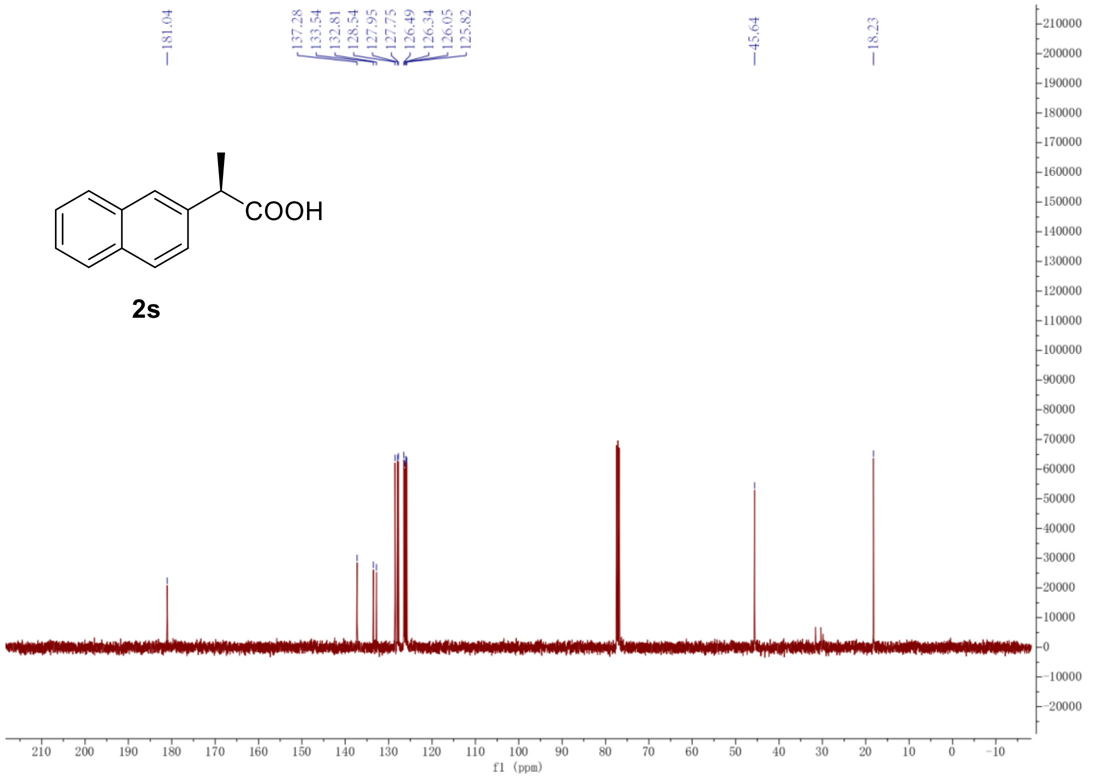

Supplementary Fig. 28 ^{1}H NMR (400 MHz) & ^{13}C NMR (101 MHz) spectra of compound **2s** in $CDCl_3$.

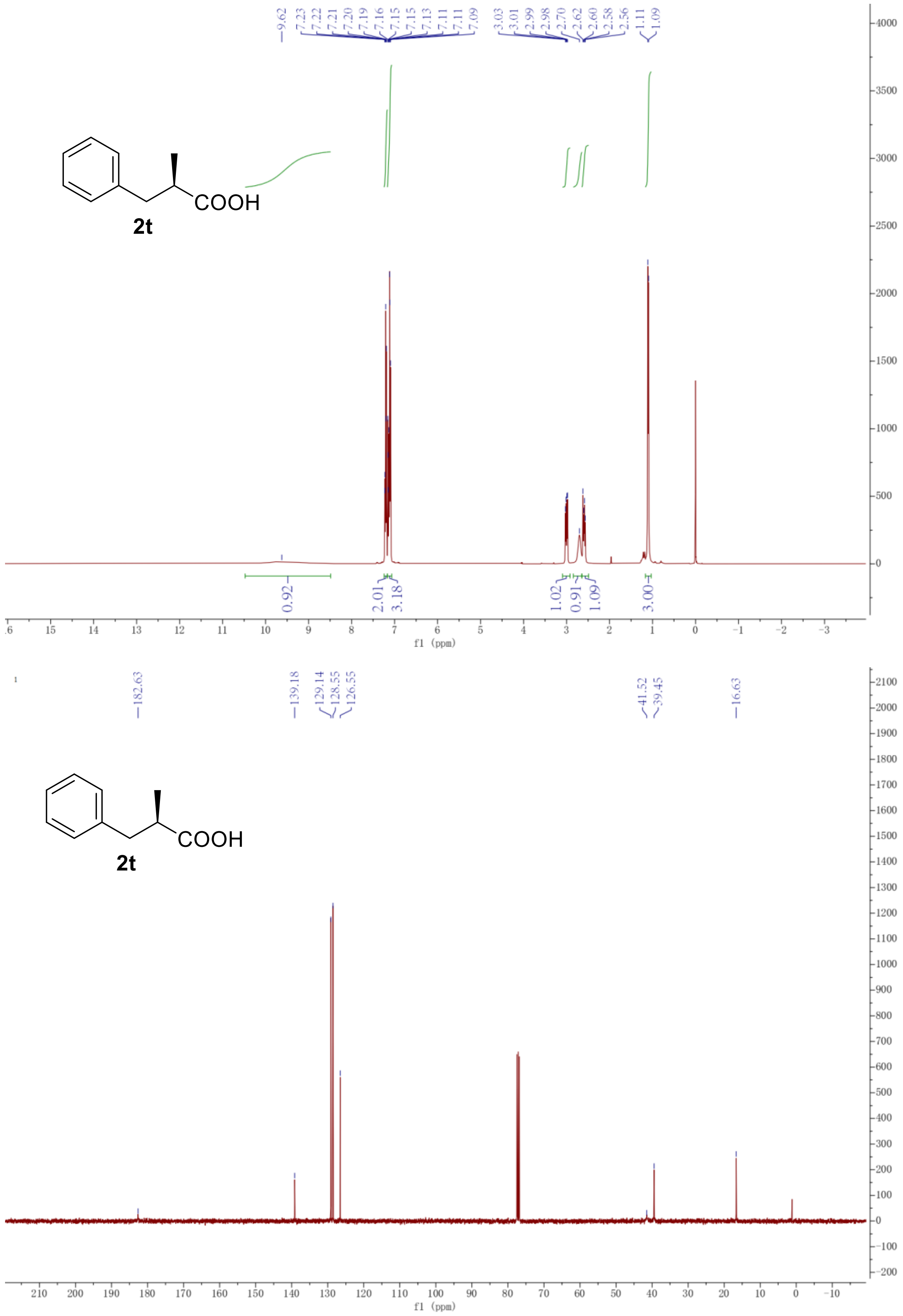

Supplementary Fig. 29 ^{1}H NMR (400 MHz) & ^{13}C NMR (101 MHz) spectra of compound **2t** in $CDCl_3$.

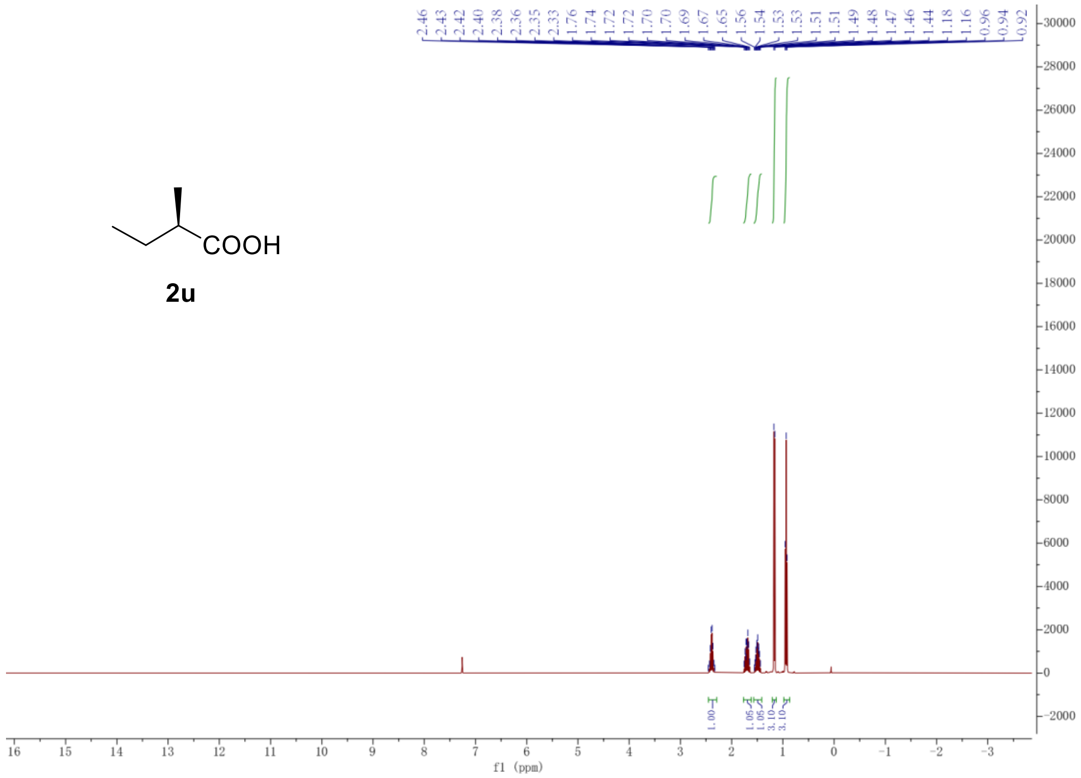

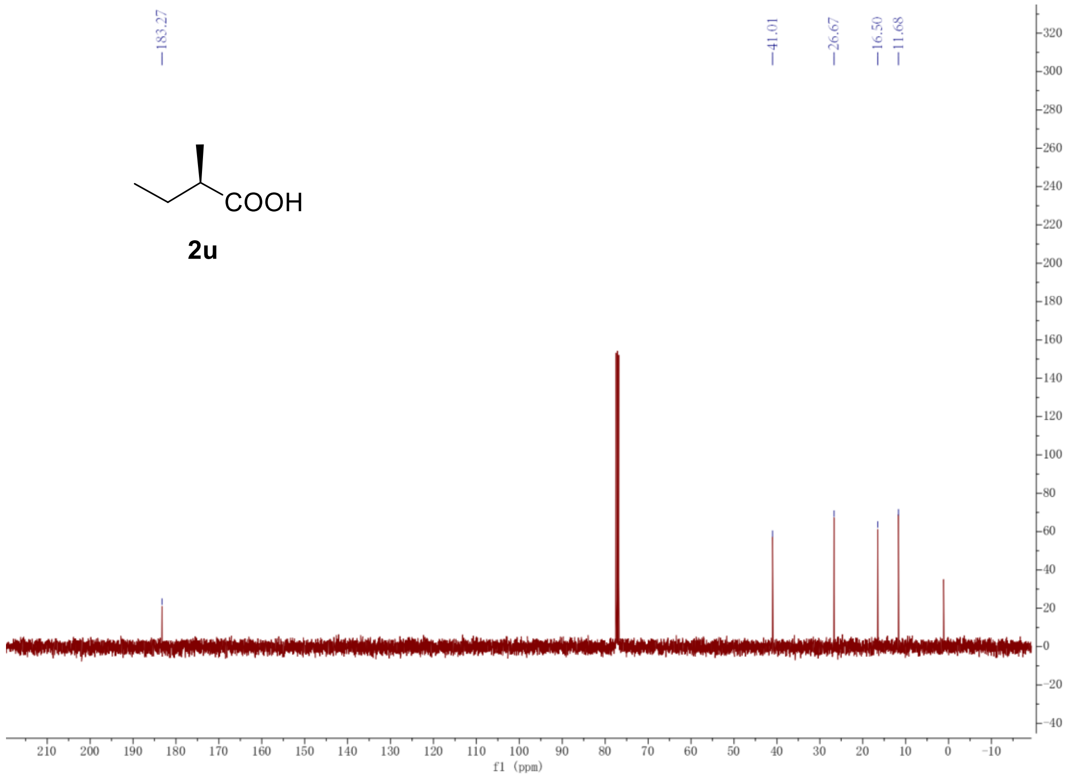

Supplementary Fig. 30 ^{1}H NMR (400 MHz) & ^{13}C NMR (101 MHz) spectra of compound **2u** in $CDCl_3$.

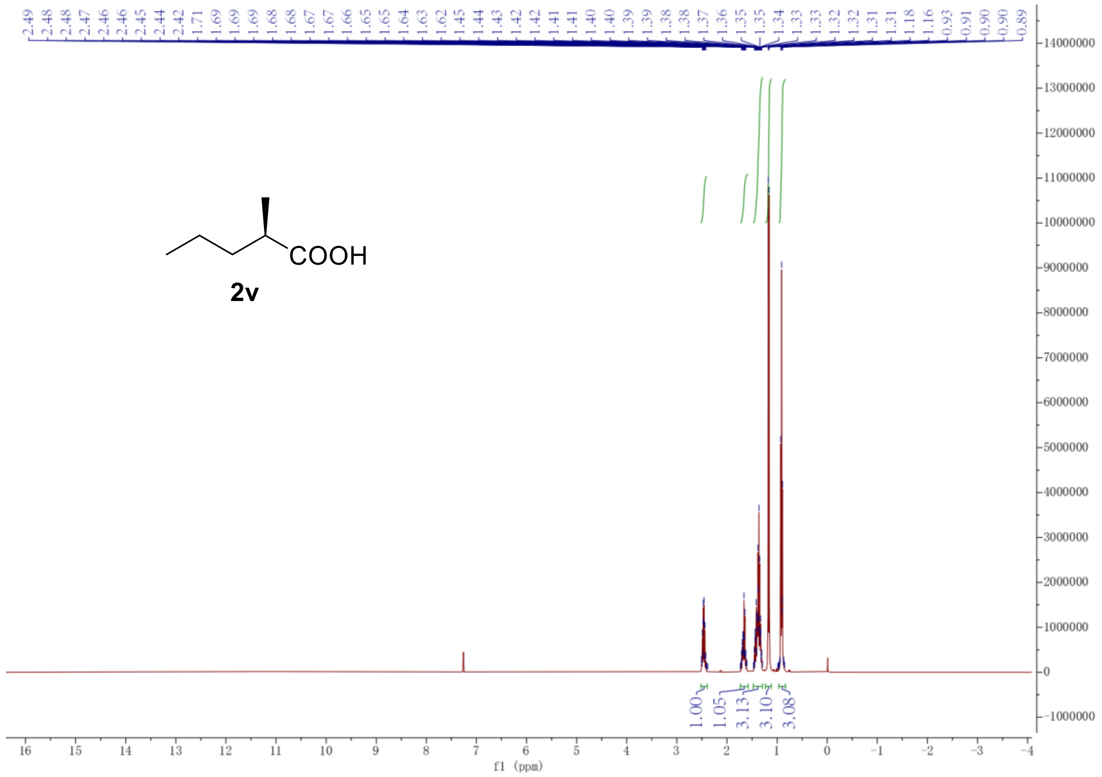

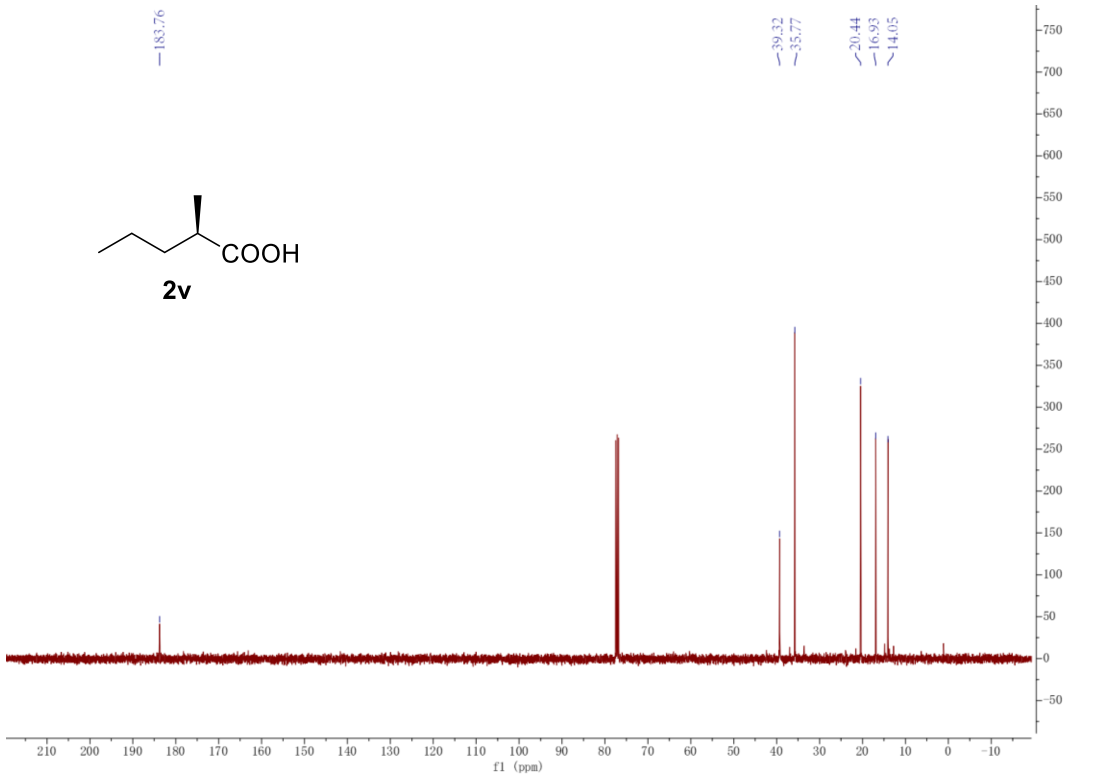

Supplementary Fig. 31 ^{1}H NMR (400 MHz) & ^{13}C NMR (101 MHz) spectra of compound **2v** in $CDCl_3$.

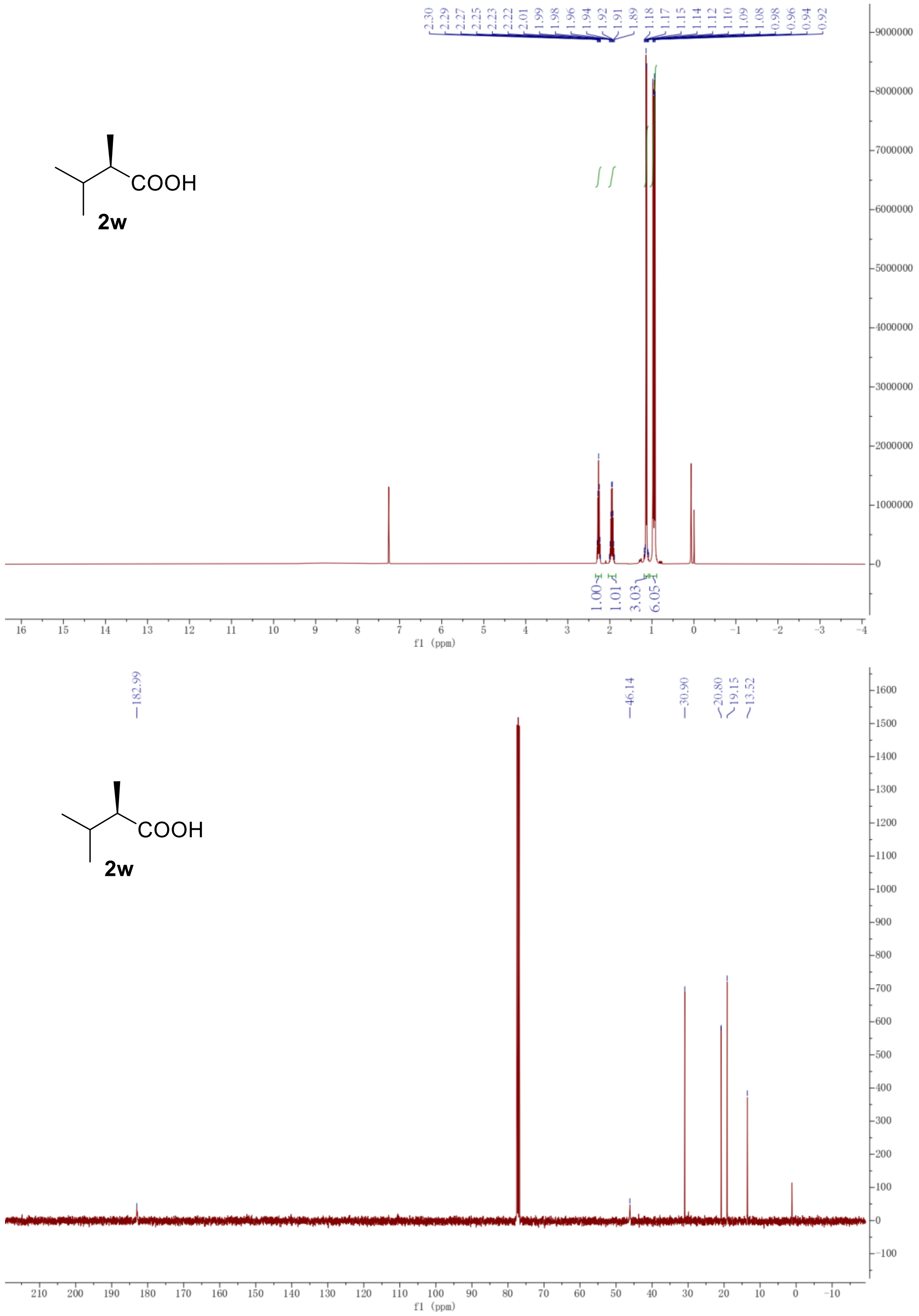

Supplementary Fig. 32 ^{1}H NMR (400 MHz) & ^{13}C NMR (101 MHz) spectra of compound **2w** in $CDCl_3$.

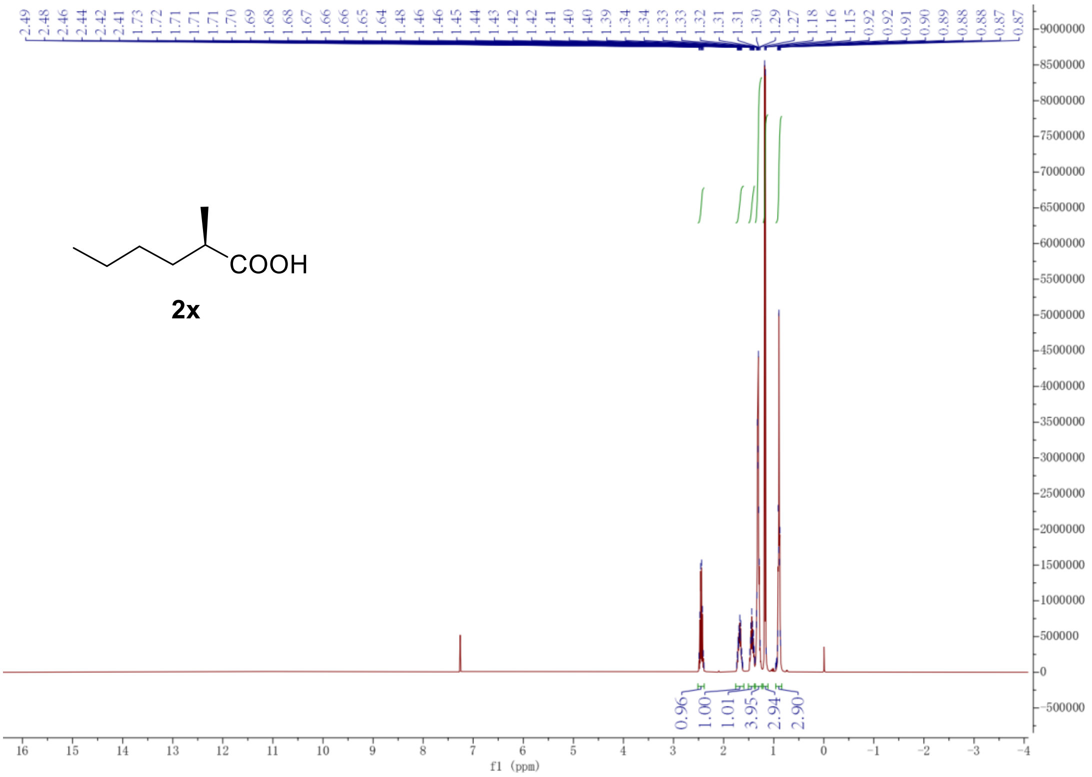

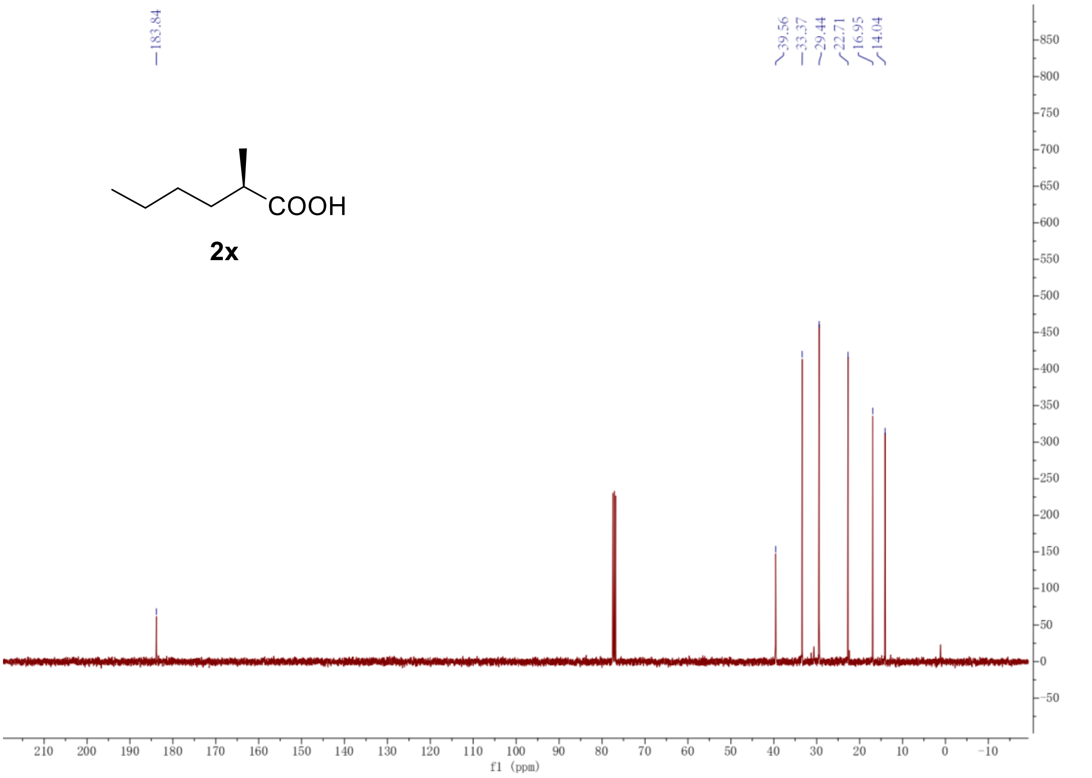

Supplementary Fig. 33 ^{1}H NMR (400 MHz) & ^{13}C NMR (101 MHz) spectra of compound **2x** in $CDCl_3$.

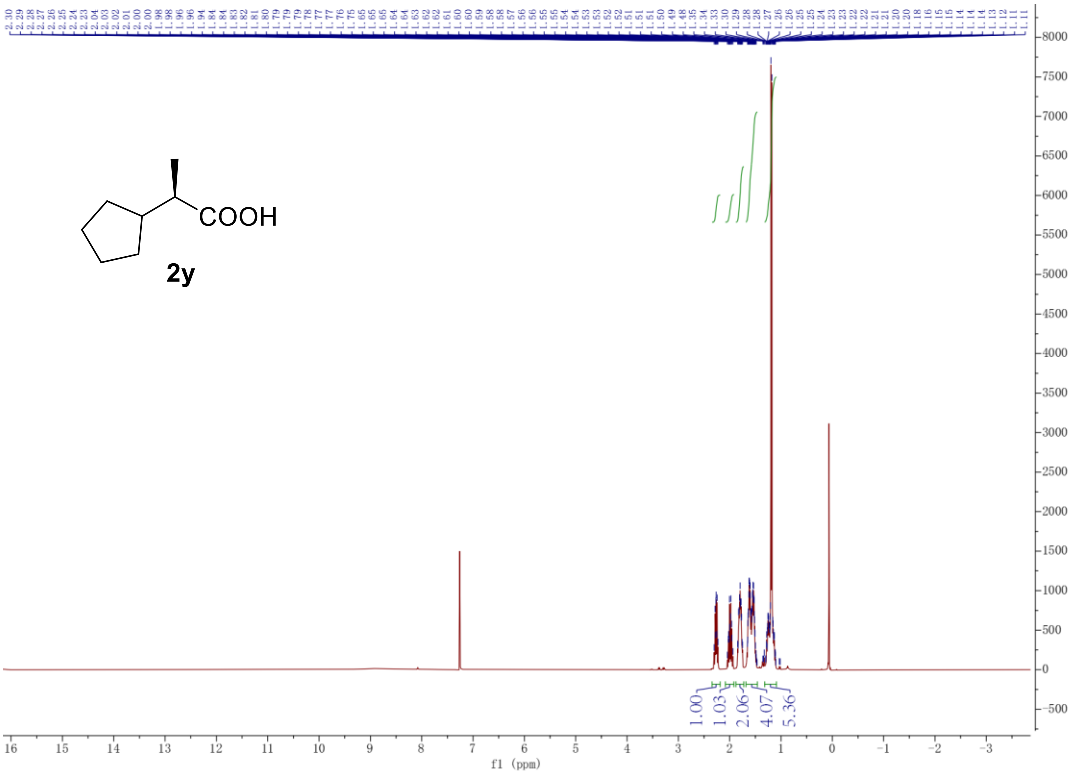

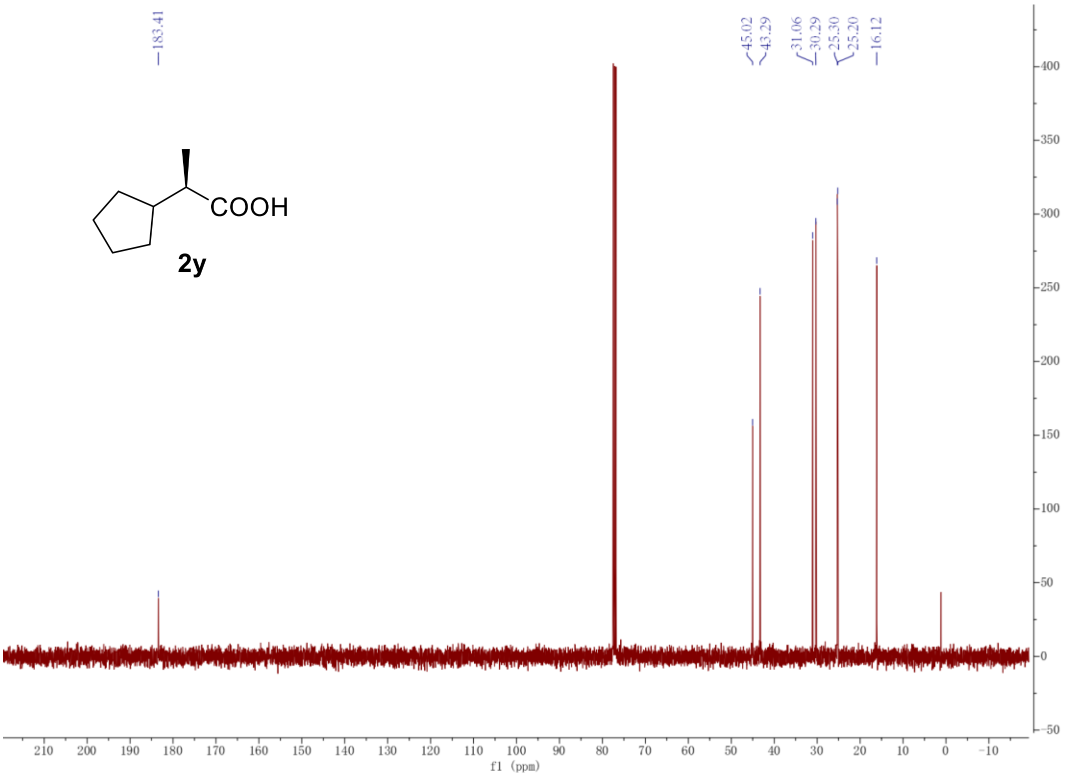

Supplementary Fig. 34 ^{1}H NMR (400 MHz) & ^{13}C NMR (101 MHz) spectra of compound **2y** in $CDCl_3$.

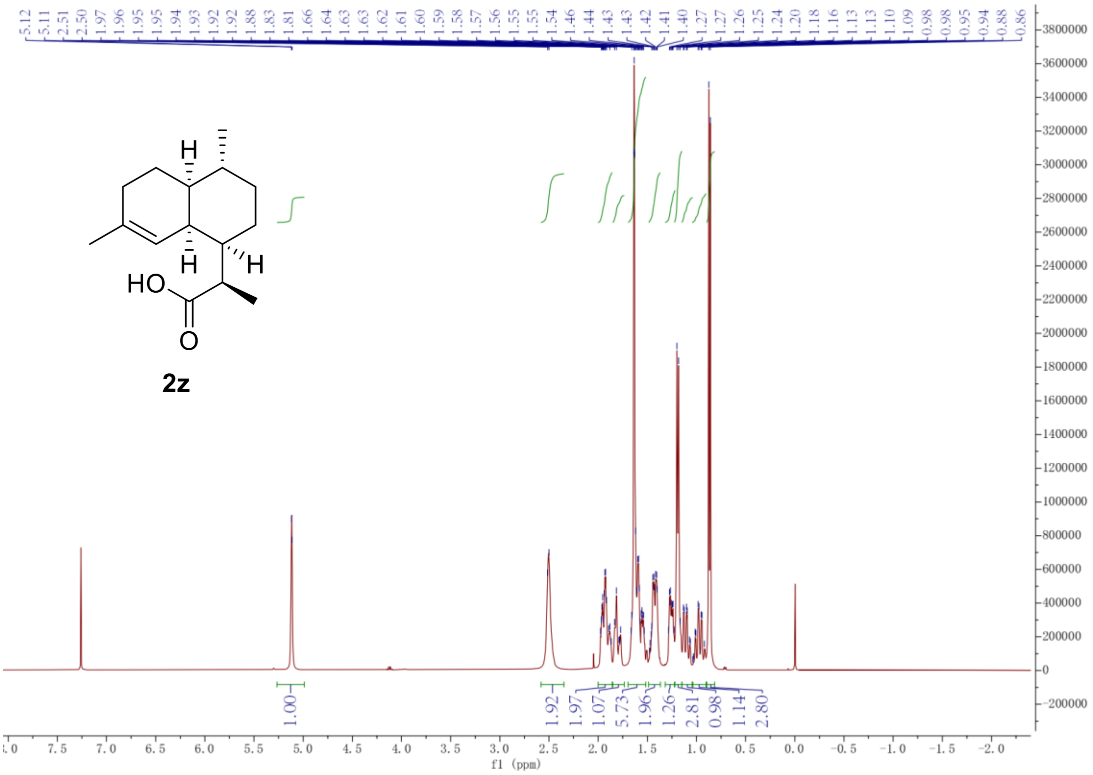

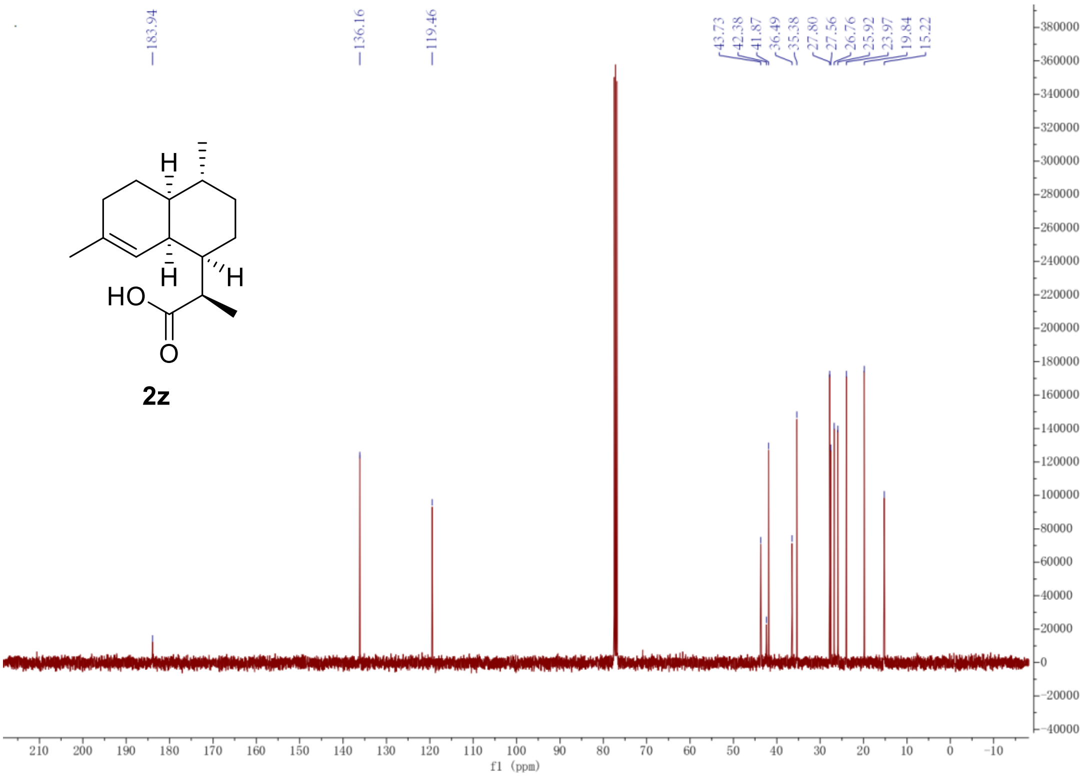

Supplementary Fig. 35 ^{1}H NMR (400 MHz) & ^{13}C NMR (101 MHz) spectra of compound **2z** in $CDCl_3$.

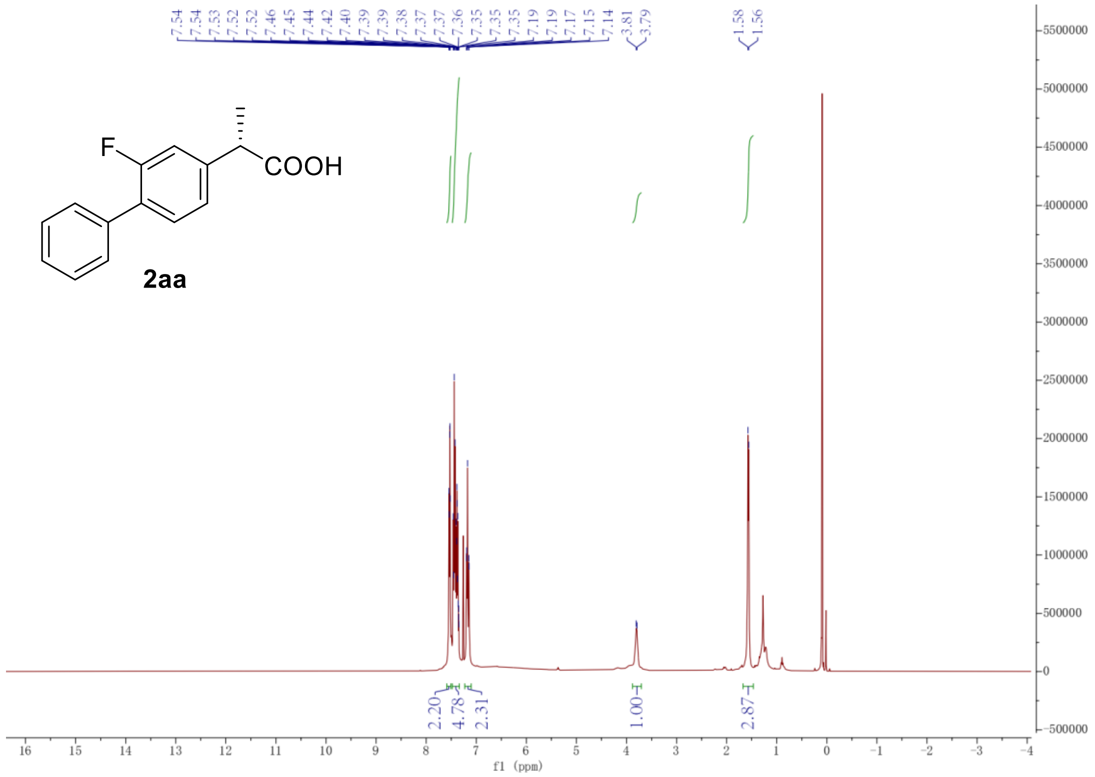
7.54 7.54 7.53 7.52 7.52 7.46 7.45 7.44 7.42 7.40 7.39 7.39 7.38 7.37 7.37 7.36 7.35 7.35 7.35 7.19 7.19 7.17 7.15 7.14 3.81 3.79 1.58 1.56
F
COOH
2aa
2.20 4.78 2.31 1.00 2.87
f1 (ppm)

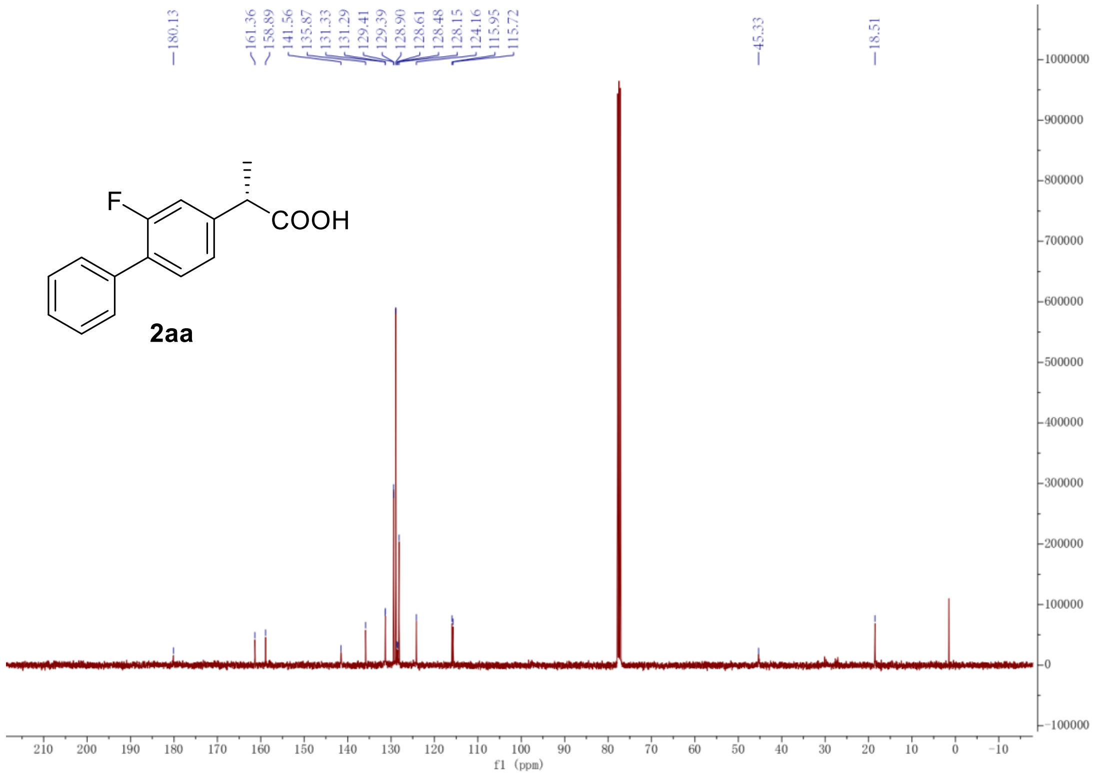
180.13 161.36 158.89 141.56 135.87 131.33 131.29 129.41 129.39 128.90 128.61 128.48 128.15 124.16 115.95 115.72 45.33 18.51
F
COOH
2aa
f1 (ppm)

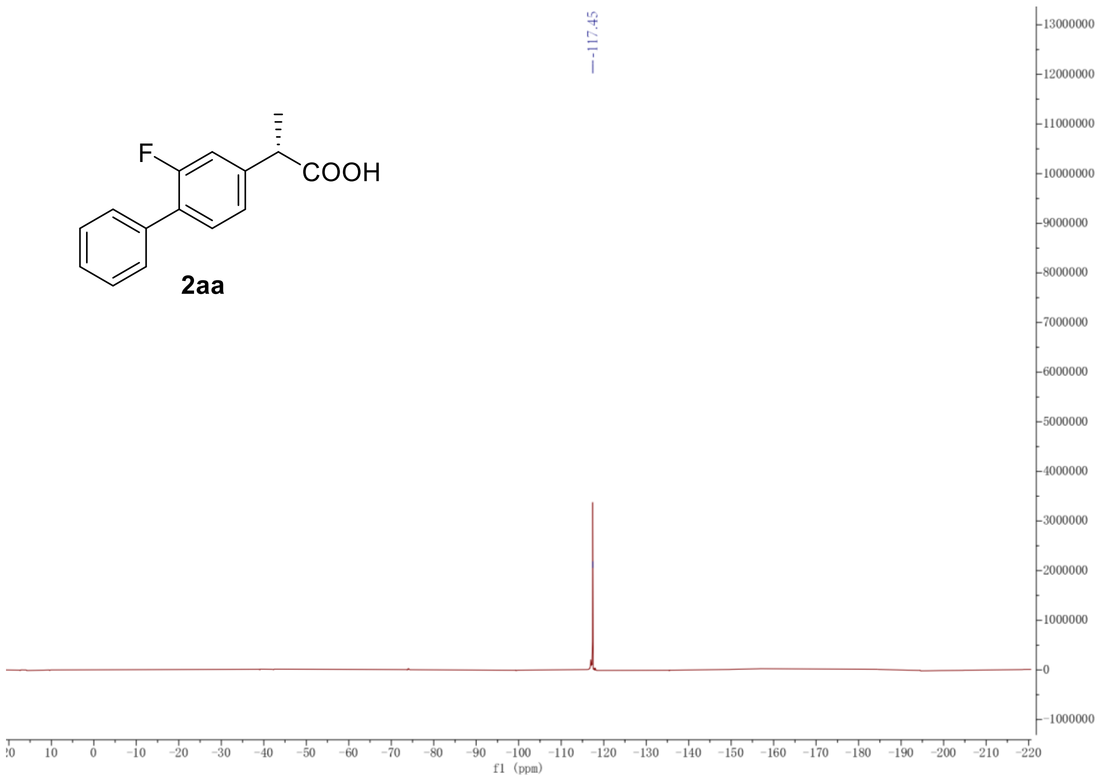

Supplementary Fig. 36 ^{1}H NMR (400 MHz), ^{13}C NMR (101 MHz) & ^{19}F NMR (376 MHz) spectra of compound **2aa** in $CDCl_3$.

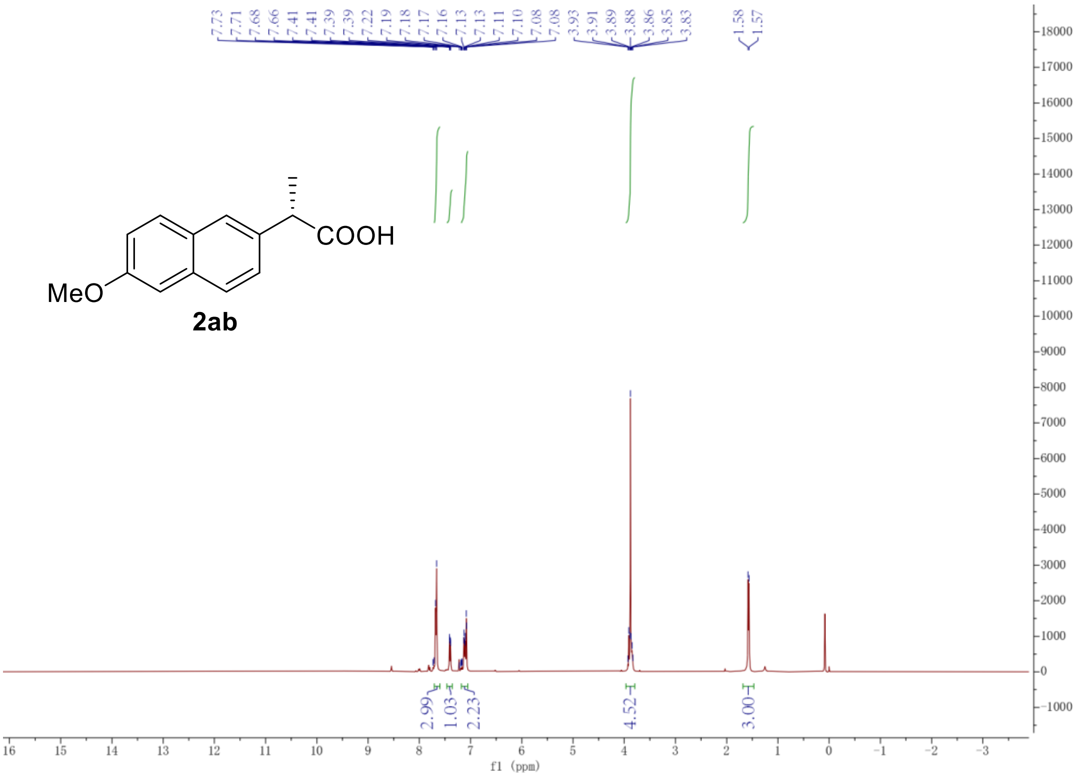

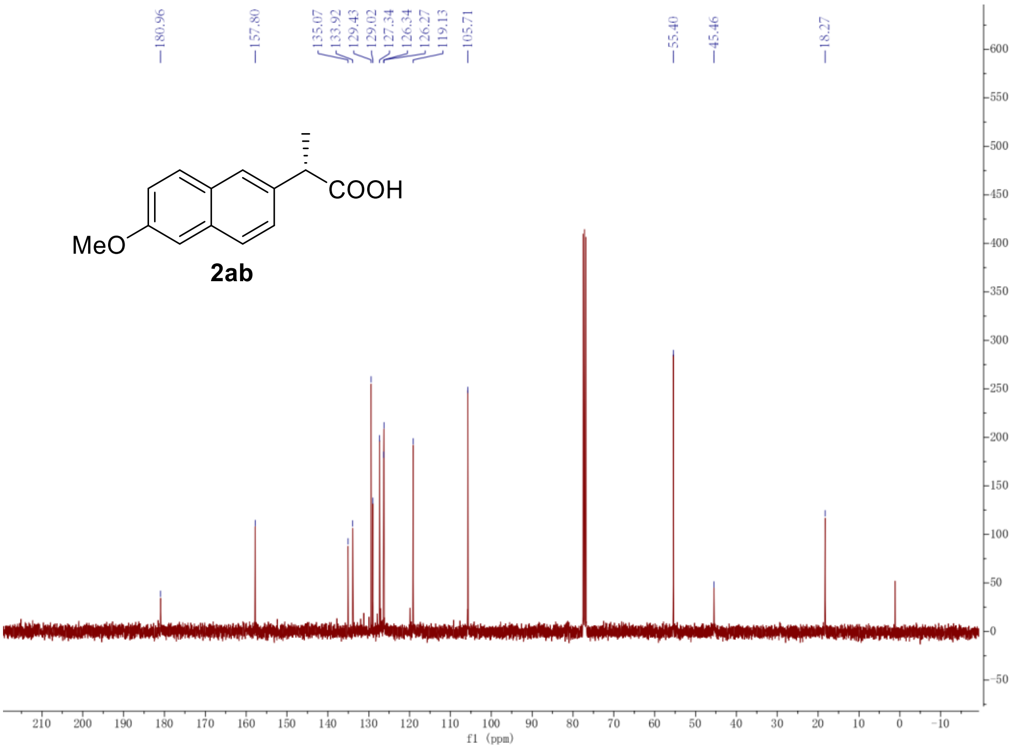

Supplementary Fig. 37 ^{1}H NMR (400 MHz), ^{13}C NMR (101 MHz) & ^{19}F NMR (376 MHz) spectra of compound **2ab** in $CDCl_3$.

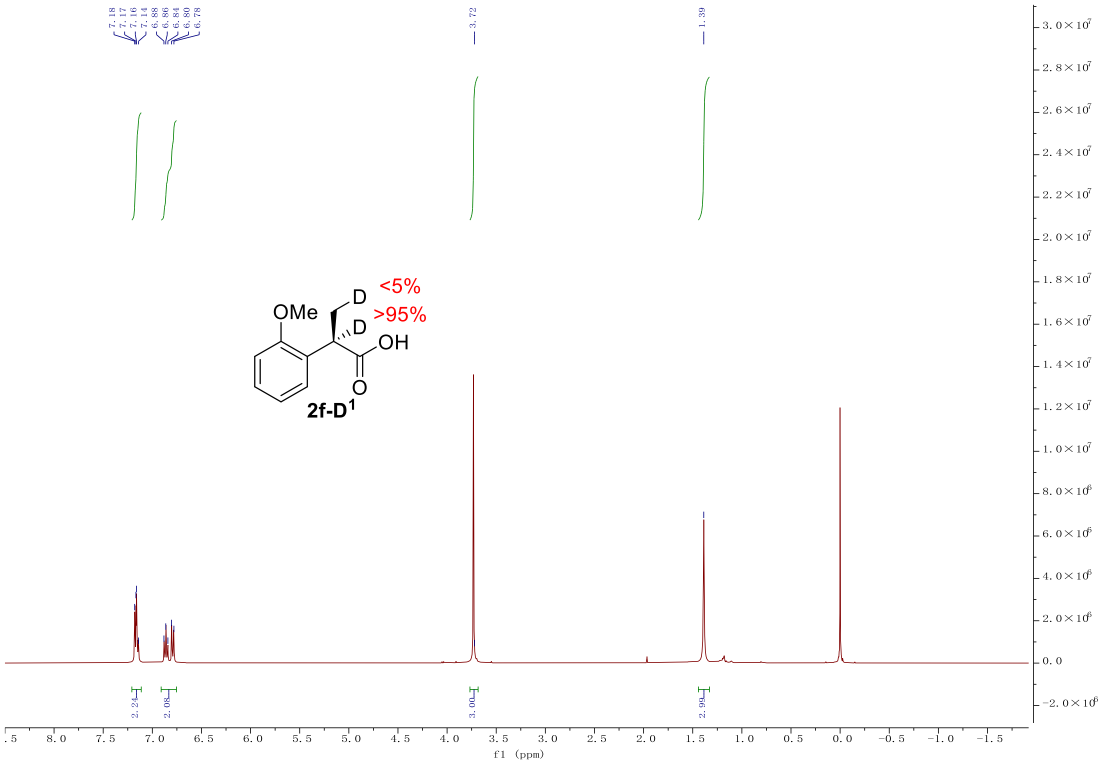

Supplementary Fig. 38 ^{1}H NMR (400 MHz) spectra of compound **2f-D^{1}** in CDCl3.

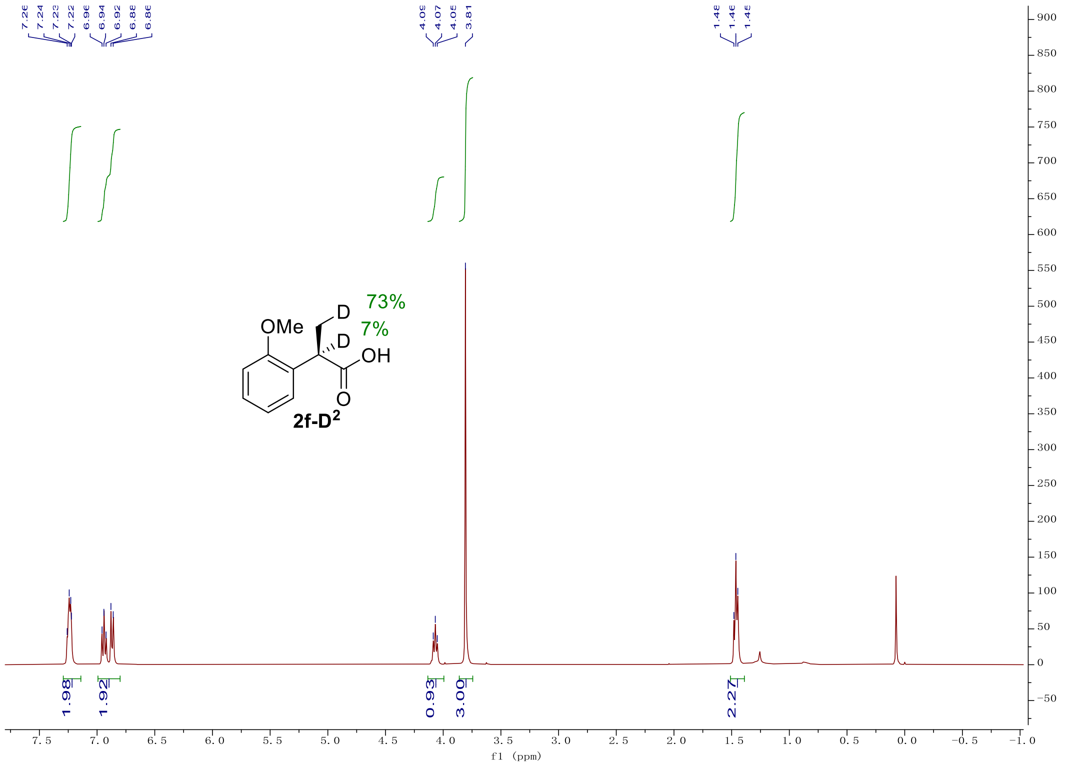

Supplementary Fig. 39 ^{1}H NMR (400 MHz) spectra of compound **2f-D^{2}** in CDCl3.

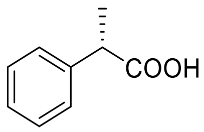

***S*-2a**

96% ee. HPLC conditions: DAICEL Chiralpak OJ-H column, Hexane/*i*PrOH = 99/1, 210 nm, 1.0 mL/min, 25 °C, t_{major} = 13.178 min, t_{minor} = 16.492 min.

Racemate

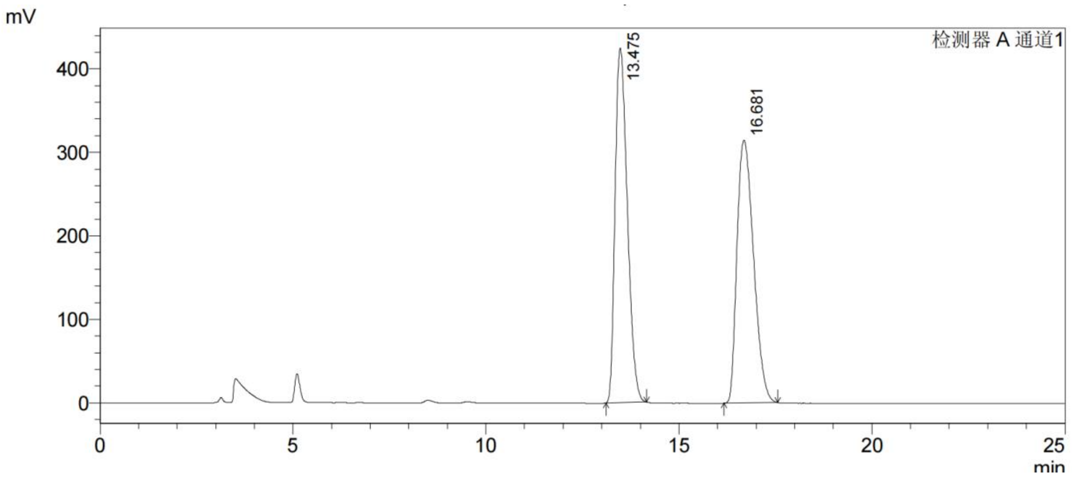

Chiral

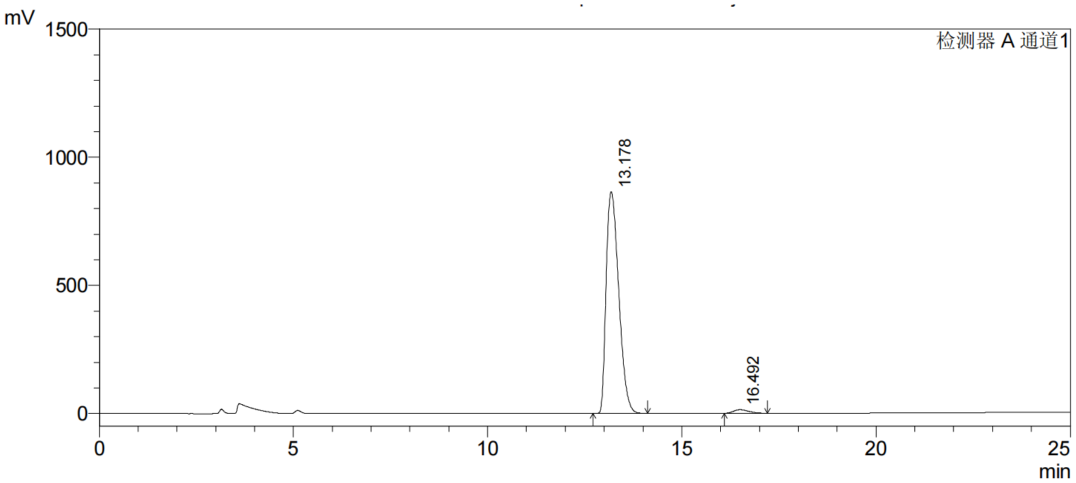

	Retention Time (min)	Relative Area (%)
Peak 1	13.178	98.081
Peak 2	16.492	1.919

Supplementary Fig. 40 HPLC spectra of compound of ***S*-2a**

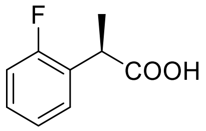

2b

97% ee. HPLC conditions: DAICEL Chiralpak OJ-H column, Hexane/*i*PrOH = 99/1, 210 nm, 1.0 mL/min, 25 ^{o}C, t_{minor} = 9.738 min, t_{major} = 10.930 min.

Racemate

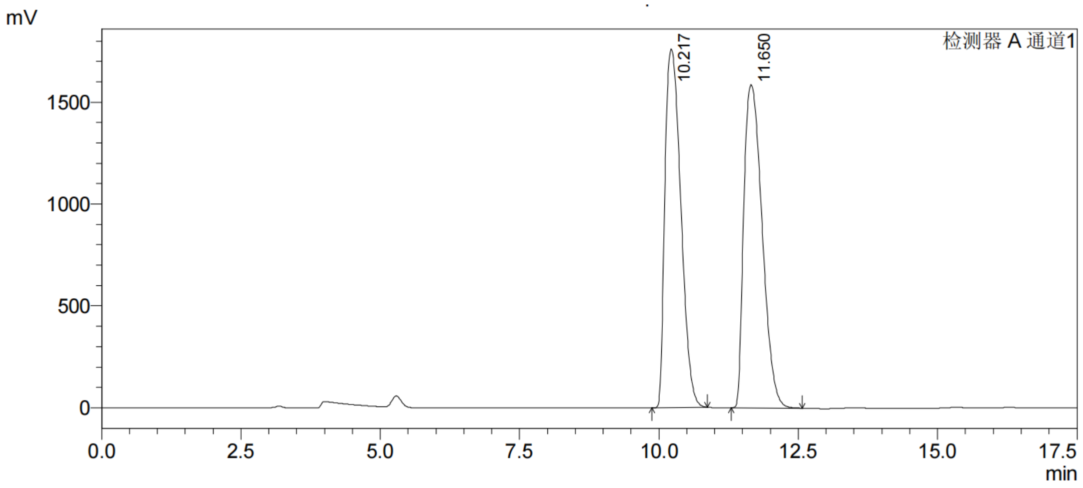

Chiral

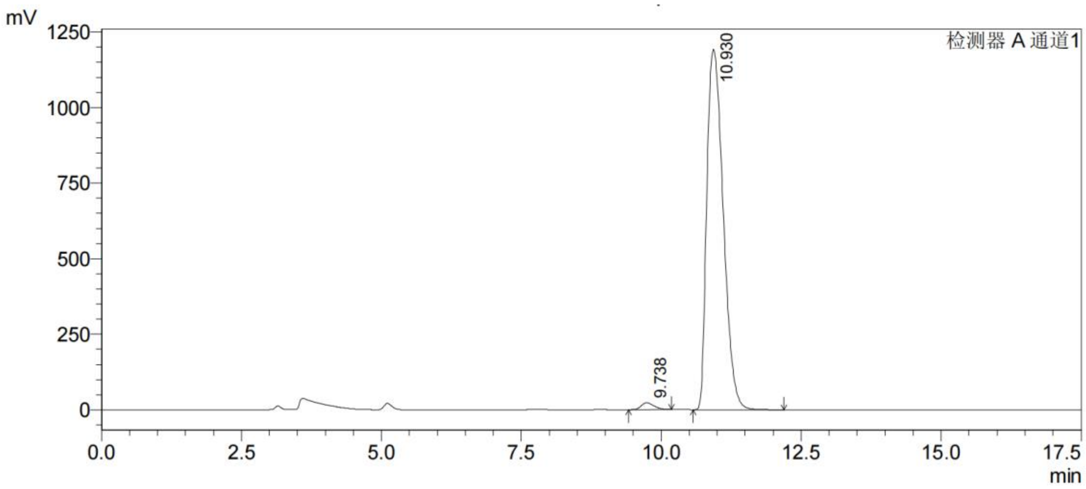

	Retention Time (min)	Relative Area (%)
Peak 1	9.738	1.418
Peak 2	10.930	98.582

Supplementary Fig. 41 HPLC spectra of compound of **2b**

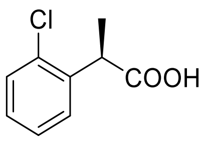

2c

99.4% ee. HPLC conditions: DAICEL Chiralpak OJ-H column, Hexane/*i*PrOH = 99/1, 210 nm, 1.0 mL/min, 25 ^{o}C, t_{major} = 10.174 min, t_{minor} = 11.196 min.

Racemate

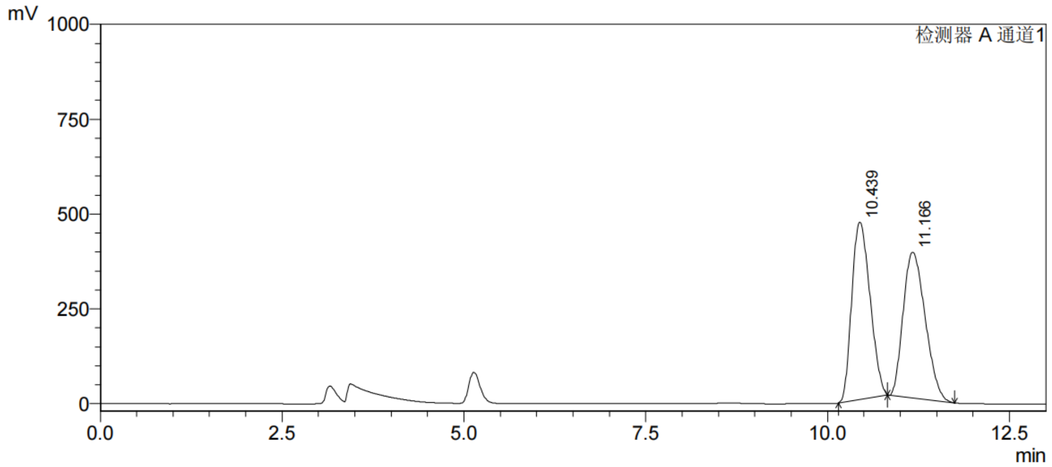

Chiral

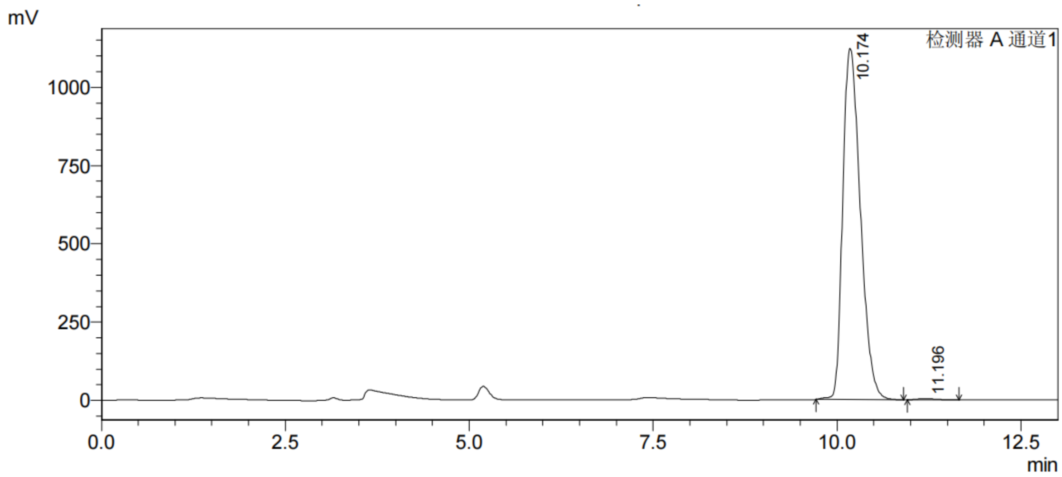

	Retention Time (min)	Relative Area (%)
Peak 1	10.174	99.688
Peak 2	11.196	0.312

Supplementary Fig. 42 HPLC spectra of compound of **2c**

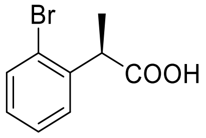

2d

98% ee. HPLC conditions: DAICEL Chiralpak OJ-H column, Hexane/*i*PrOH = 99/1, 210 nm, 1.0 mL/min, 25 °C, t_{major} = 11.199 min, t_{minor} = 13.513 min.

Racemate

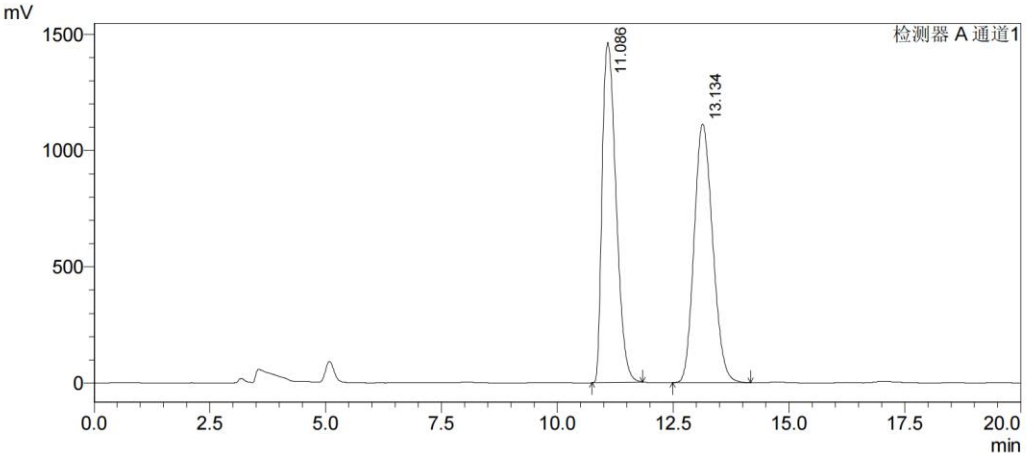

Chiral

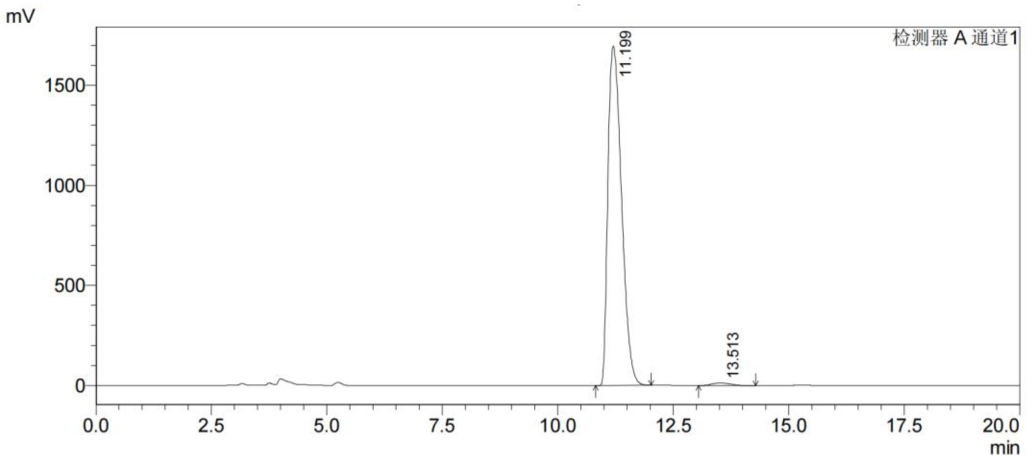

	Retention Time (min)	Relative Area (%)
Peak 1	11.199	98.976
Peak 2	13.513	1.024

Supplementary Fig. 43 HPLC spectra of compound of **2d**

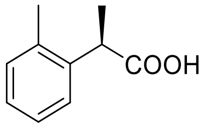

2e

99.1% ee. HPLC conditions: DAICEL Chiralpak OJ-H column, Hexane/*i*PrOH = 99/1, 210 nm, 1.0 mL/min, 25 ^{o}C, t_{major} = 8.829 min, t_{minor} = 9.778 min.

Racemate

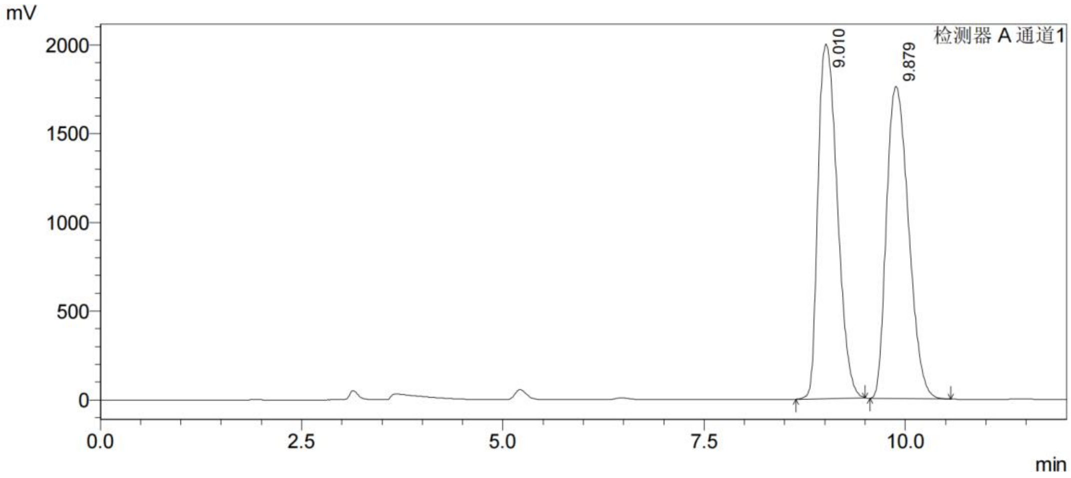

Chiral

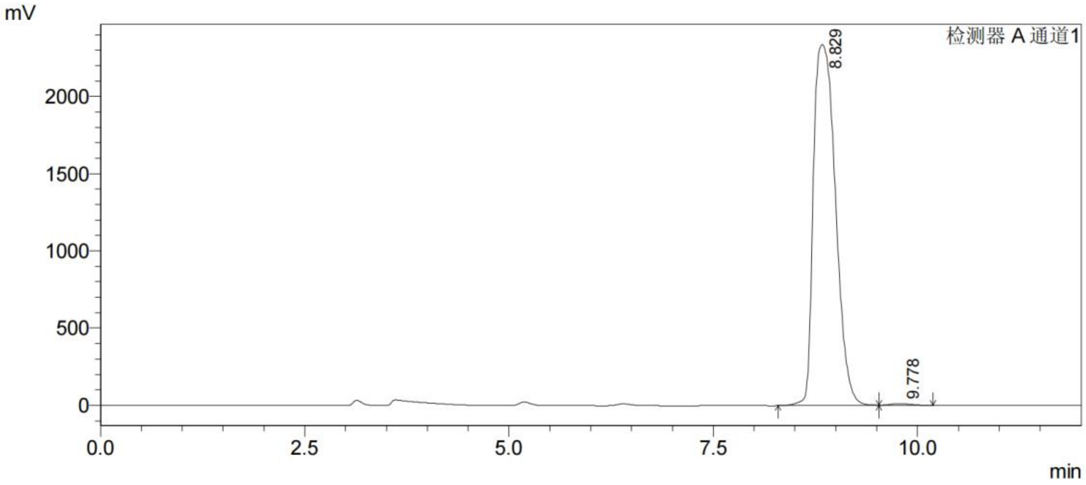

	Retention Time (min)	Relative Area (%)
Peak 1	8.829	99.540
Peak 2	9.778	0.460

Supplementary Fig. 44 HPLC spectra of compound of **2e**

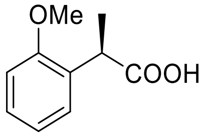

2f

98% ee. HPLC conditions: DAICEL Chiralpak OJ-H column, Hexane/*i*PrOH = 99/1, 210 nm, 1.0 mL/min, 25 ºC, t_{minor} = 13.681 min, t_{major} = 16.288 min.

Racemate

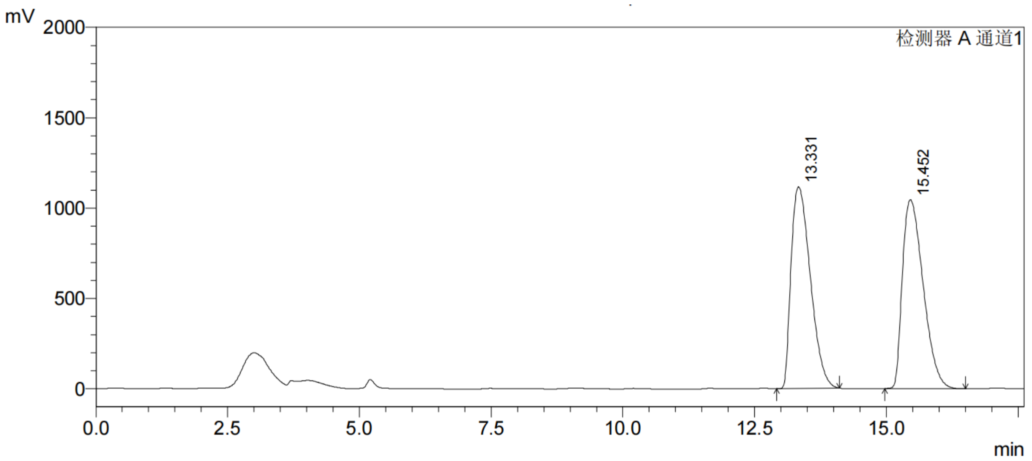

Chiral

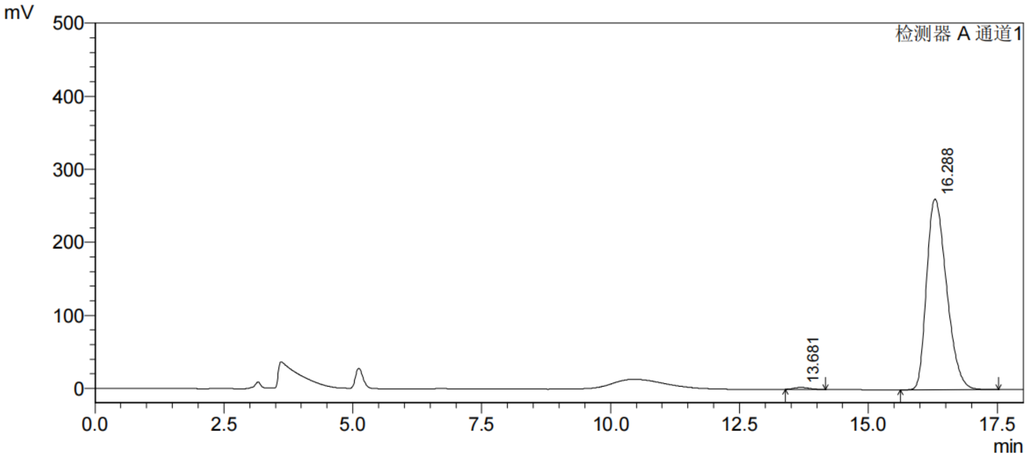

	Retention Time (min)	Relative Area (%)
Peak 1	13.681	0.785
Peak 2	16.288	99.215

Supplementary Fig. 45 HPLC spectra of compound of **2f**

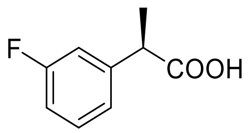

2g

96% ee. HPLC conditions: DAICEL Chiralpak OJ-H column, Hexane/*i*PrOH = 99/1, 210 nm, 1.0 mL/min, 25 °C, t_{minor} = 8.570 min, t_{major} = 9.185 min.

Racemate

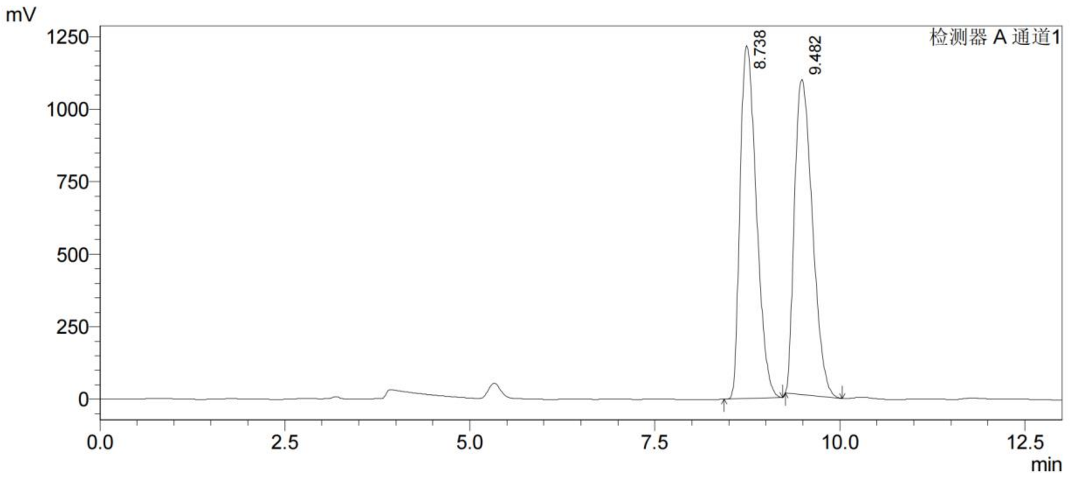

Chiral

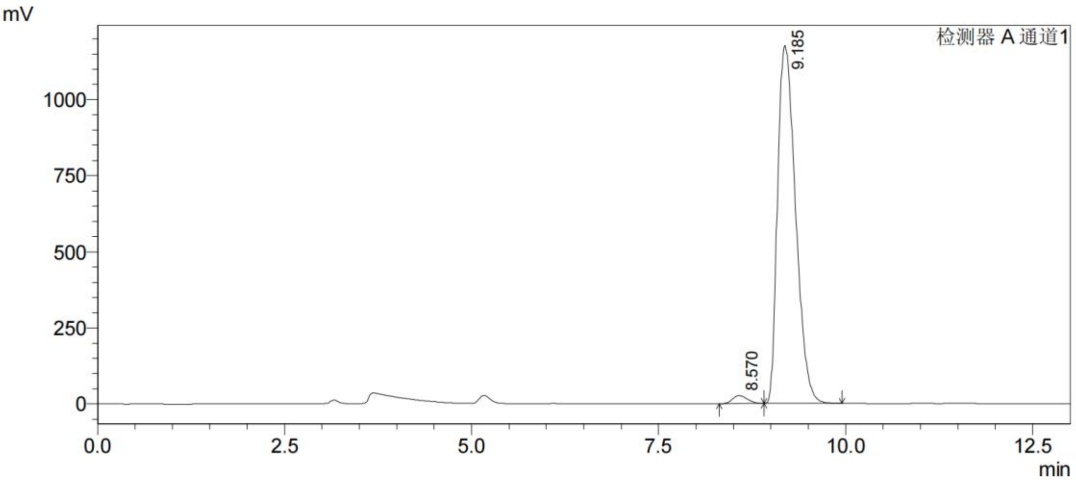

	Retention Time (min)	Relative Area (%)
Peak 1	8.570	1.818
Peak 2	9.185	98.182

Supplementary Fig. 46 HPLC spectra of compound of **2g**

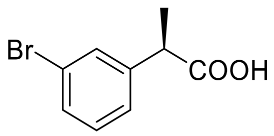

2h

96% ee. HPLC conditions: DAICEL Chiralpak OJ-H column, Hexane/*i*PrOH = 99/1, 210 nm, 1.0 mL/min, 25 ^{o}C, t_{minor} = 9.255 min, t_{major} = 9.711 min.

Racemate

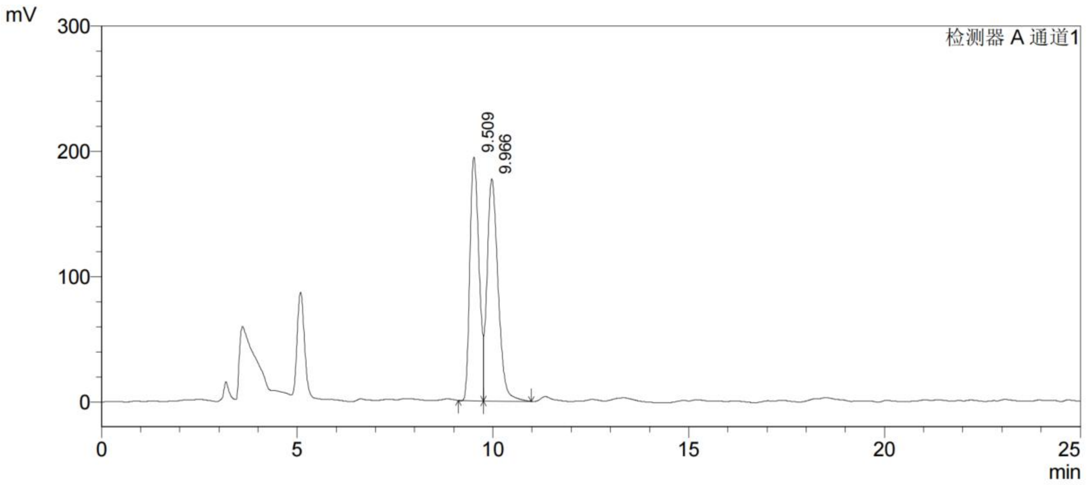

Chiral

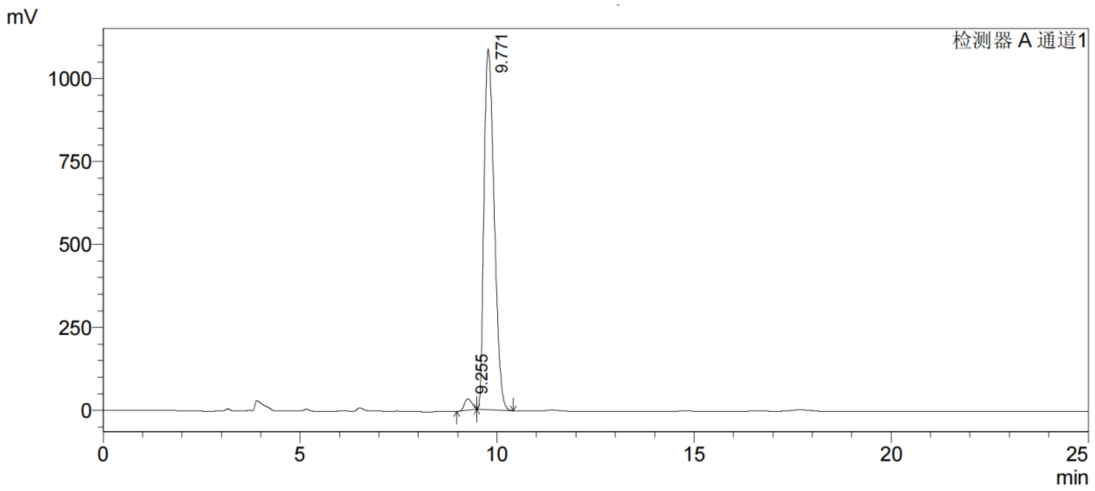

	Retention Time (min)	Relative Area (%)
Peak 1	9.255	2.192
Peak 2	9.711	97.808

Supplementary Fig. 47 HPLC spectra of compound of **2h**

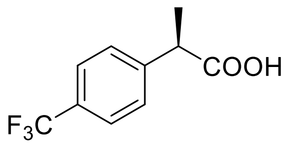

2i

96% ee. HPLC conditions: DAICEL Chiralpak OJ-H column, Hexane/*i*PrOH = 99/1, 210 nm, 1.0 mL/min, 25 ^{o}C, t_{major} = 6.025 min, t_{minor} = 7.259 min.

Racemate

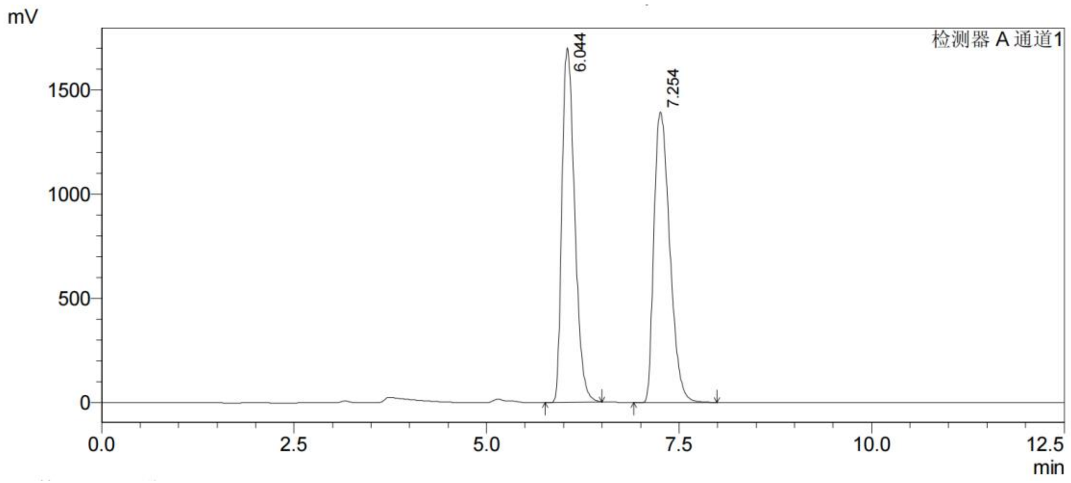

Chiral

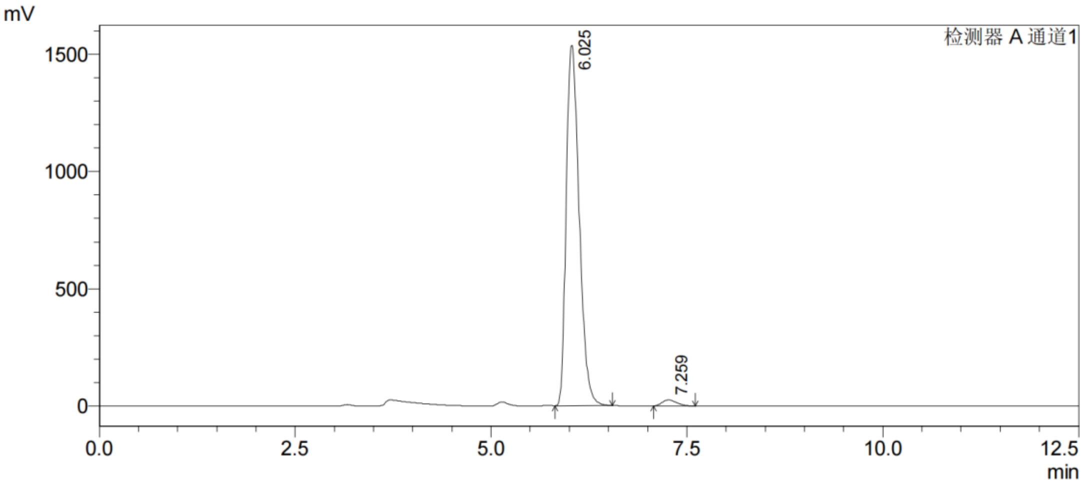

	Retention Time (min)	Relative Area (%)
Peak 1	6.025	98.122
Peak 2	7.259	1.878

Supplementary Fig. 48 HPLC spectra of compound of **2i**

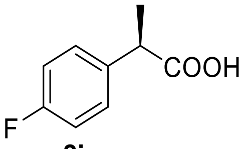

2j

95% ee. HPLC conditions: DAICEL Chiralpak OJ-H column, Hexane/*i*PrOH = 99/1, 210 nm, 1.0 mL/min, 25 ^{o}C, t_{major} = 10.727 min, t_{minor} = 11.900 min.

Racemate

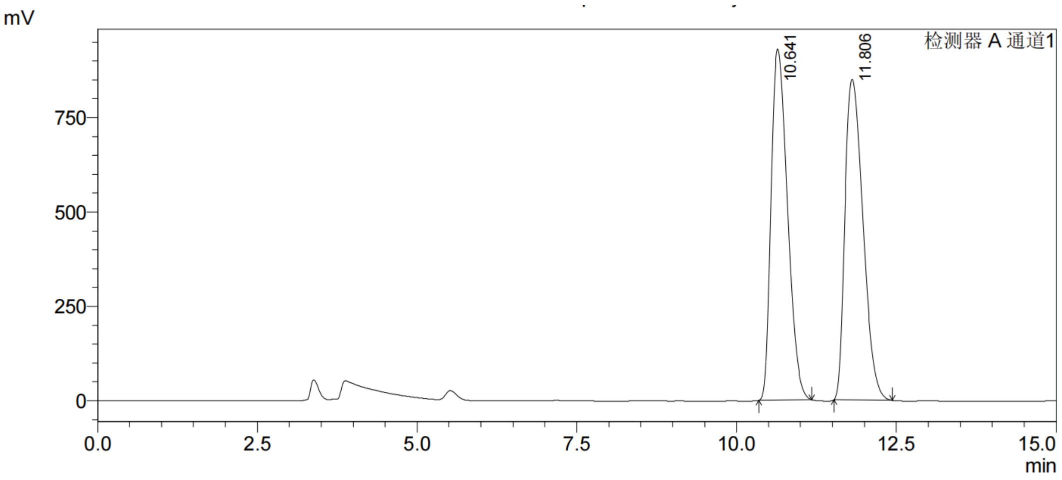

Chiral

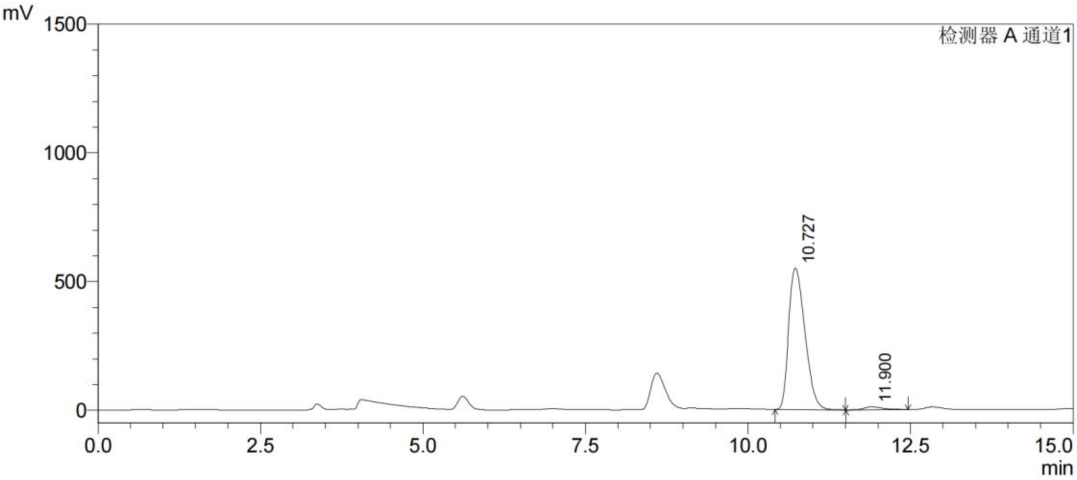

	Retention Time (min)	Relative Area (%)
Peak 1	10.727	97.605
Peak 2	11.900	2.395

Supplementary Fig. 49 HPLC spectra of compound of **2j**

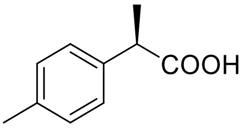

2k

96% ee. HPLC conditions: DAICEL Chiralpak OJ-H column, Hexane/*i*PrOH = 99/1, 210 nm, 1.0 mL/min, 25 ^{o}C, t_{major} = 13.731 min, t_{minor} = 16.157 min.

Racemate

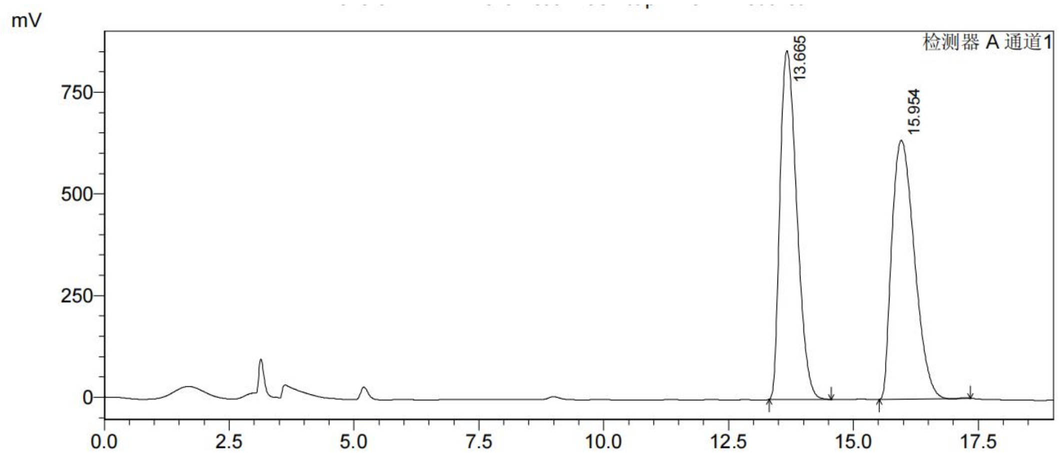

Chiral

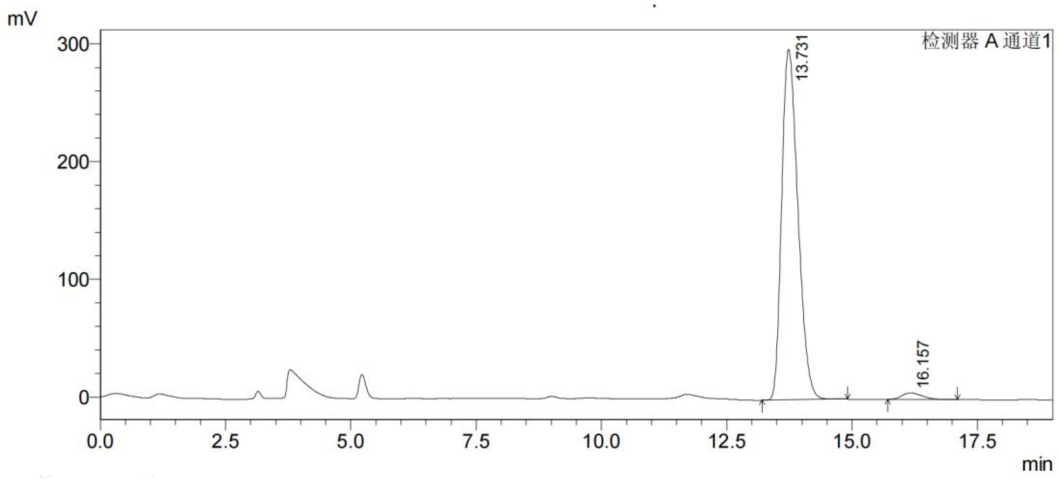

	Retention Time (min)	Relative Area (%)
Peak 1	13.731	97.737
Peak 2	16.157	2.263

Supplementary Fig. 50 HPLC spectra of compound of **2k**

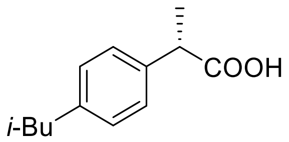

***S*-2l**

96% ee. HPLC conditions: DAICEL Chiralpak OJ-H column, Hexane/*i*PrOH = 99/1, 210 nm, 1.0 mL/min, 25 °C, t_{major} = 8.333 min, t_{minor} = 9.236 min.

Racemate

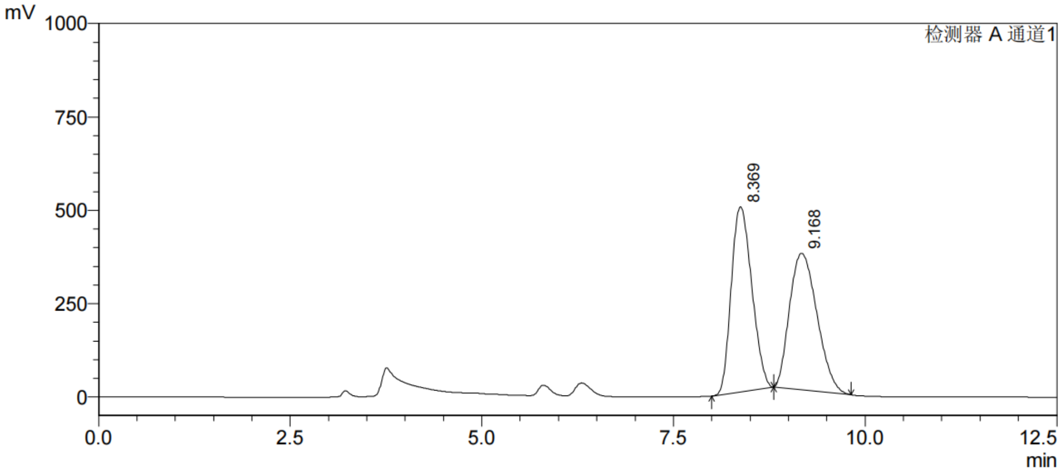

Chiral

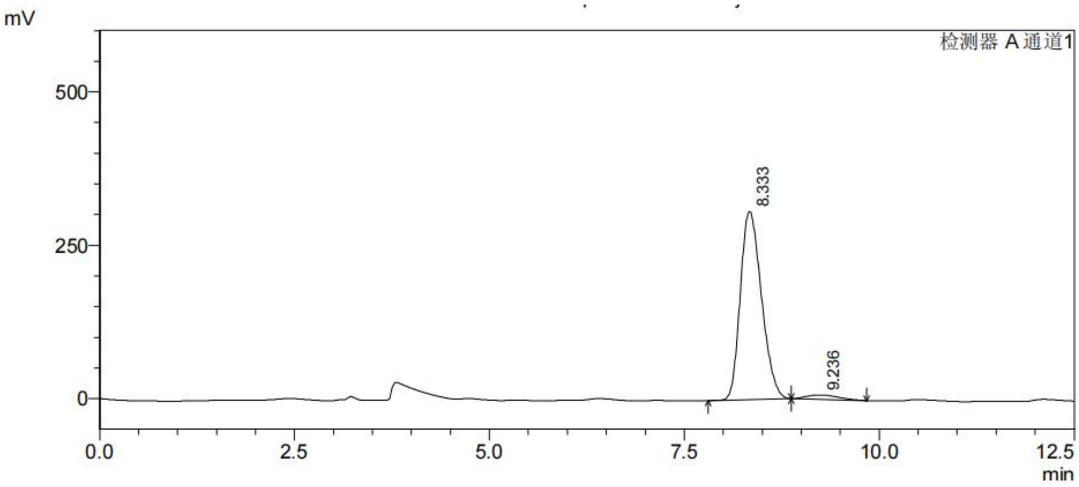

	Retention Time (min)	Relative Area (%)
Peak 1	8.333	98.036
Peak 2	9.236	1.964

Supplementary Fig. 51 HPLC spectra of compound of ***S*-2l**

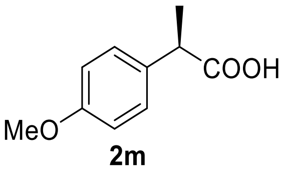

2m

96% ee. HPLC conditions: DAICEL Chiralpak OJ-H column, Hexane/*i*PrOH = 99/1, 210 nm, 1.0 mL/min, 25 ^{o}C, t_{minor} = 26.306 min, t_{major} = 28.620 min.

Racemate

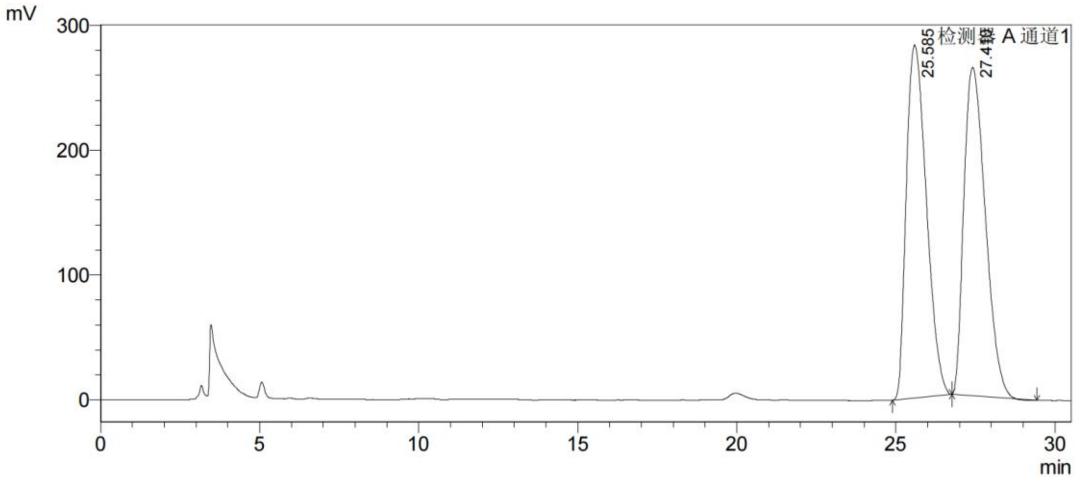

Chiral

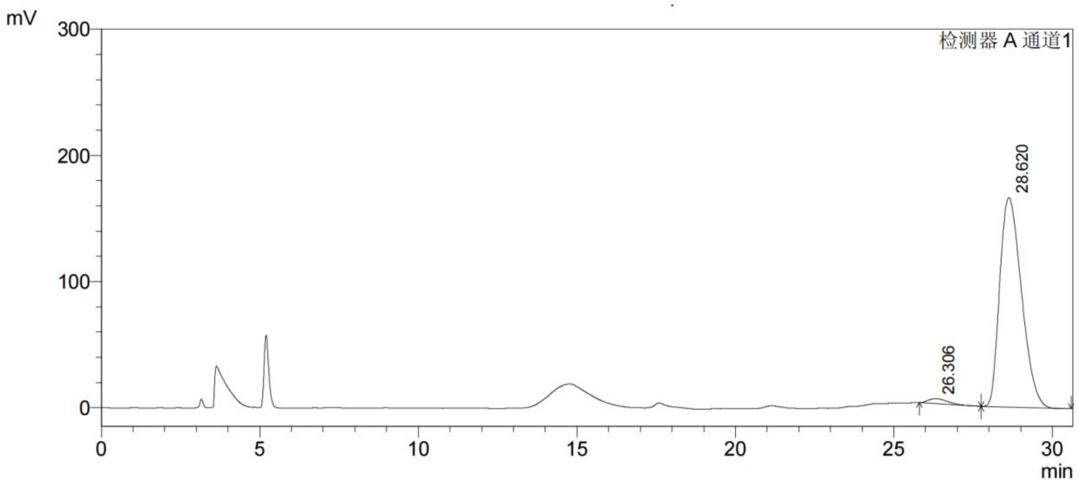

	Retention Time (min)	Relative Area (%)
Peak 1	26.306	2.060
Peak 2	28.620	97.940

Supplementary Fig. 52 HPLC spectra of compound of **2m**

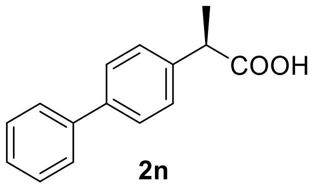

2n

92% ee. HPLC conditions: DAICEL Chiralpak OJ-H column, Hexane/*i*PrOH = 99/1, 210 nm, 1.0 mL/min, 25 ^{o}C, t_{major} = 26.412 min, t_{minor} = 34.517 min.

Racemate

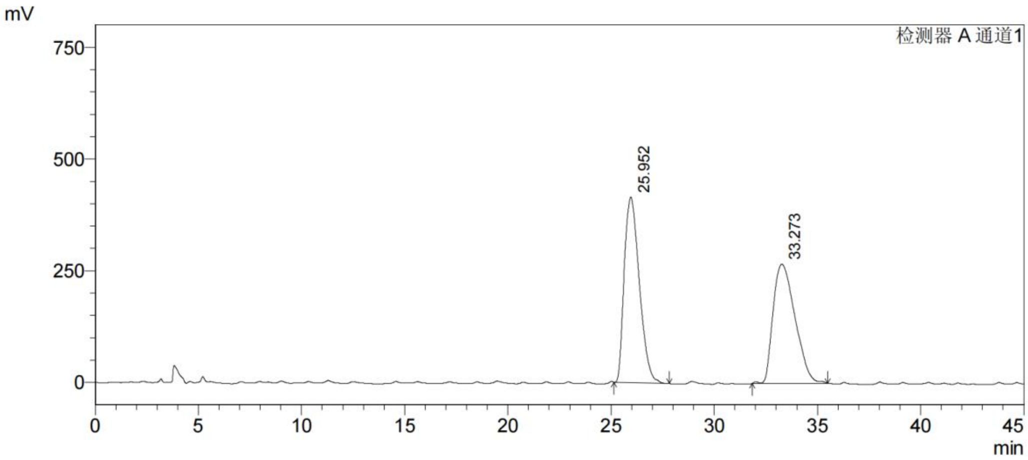

Chiral

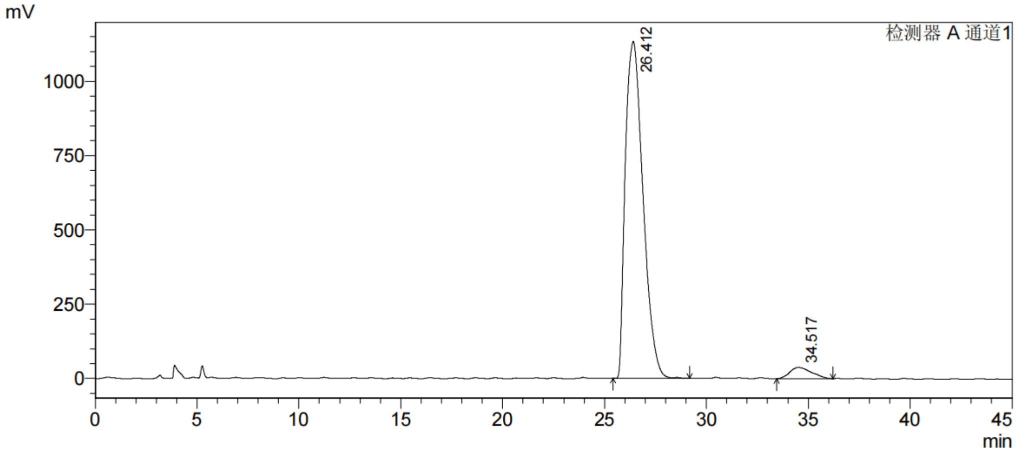

	Retention Time (min)	Relative Area (%)
Peak 1	26.412	96.057
Peak 2	34.517	3.943

Supplementary Fig. 53 HPLC spectra of compound of **2n**

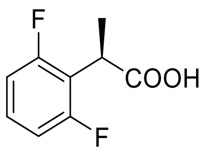

2o

99.2% ee. HPLC conditions: DAICEL Chiralpak OJ-H column, Hexane/*i*PrOH = 99/1, 210 nm, 1.0 mL/min, 25 ^oC, t_{major} = 8.233 min, t_{minor} = 8.809 min.

Racemate

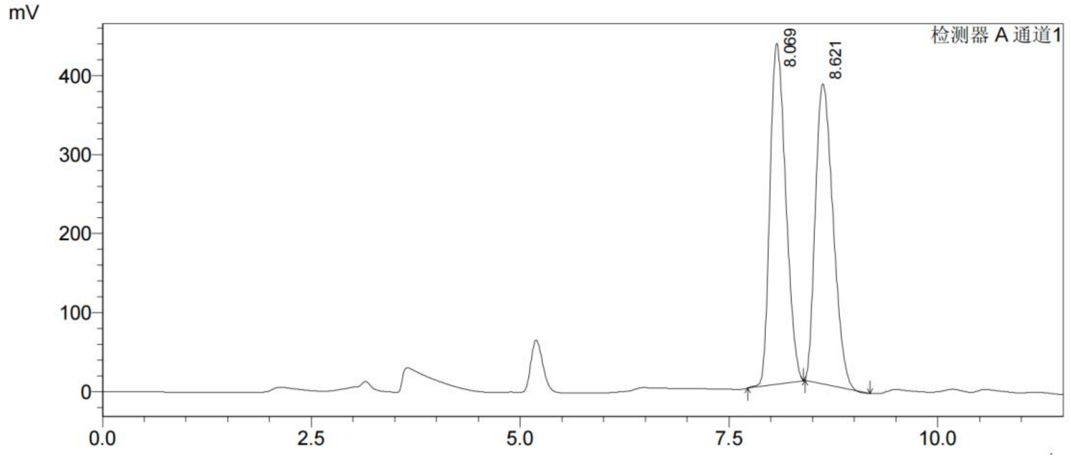

Chiral

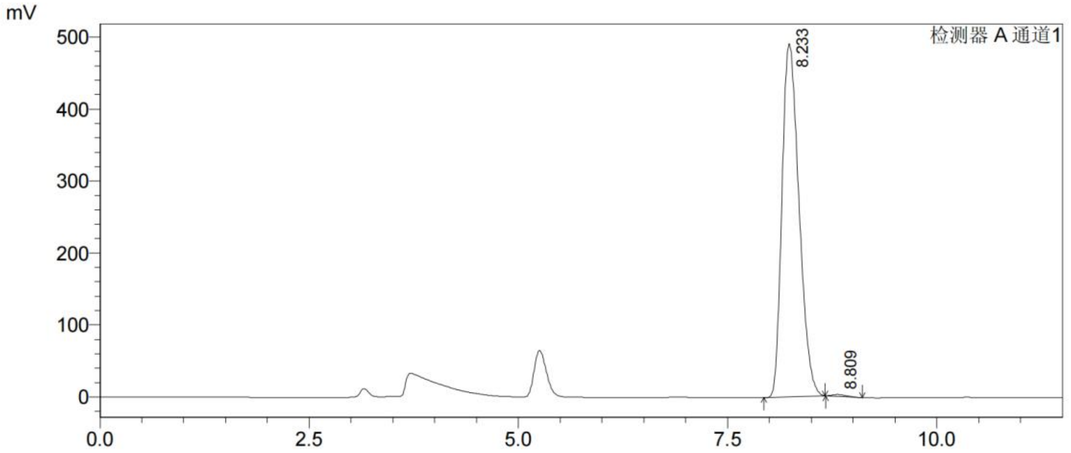

	Retention Time (min)	Relative Area (%)
Peak 1	8.233	99.581
Peak 2	8.809	0.419

Supplementary Fig. 54 HPLC spectra of compound of **2o**

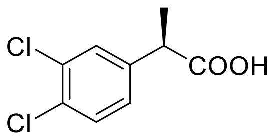

2p

95% ee. HPLC conditions: DAICEL Chiralpak OJ-H column, Hexane/*i*PrOH = 99/1, 210 nm, 1.0 mL/min, 25 ^{o}C, t_{major} = 9.207 min, t_{minor} = 9.902 min.

Racemate

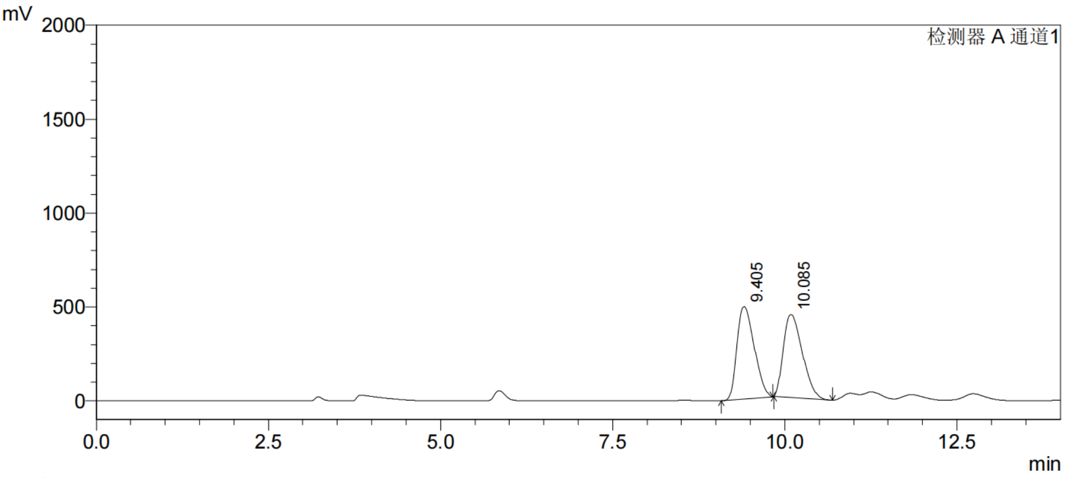

Chiral

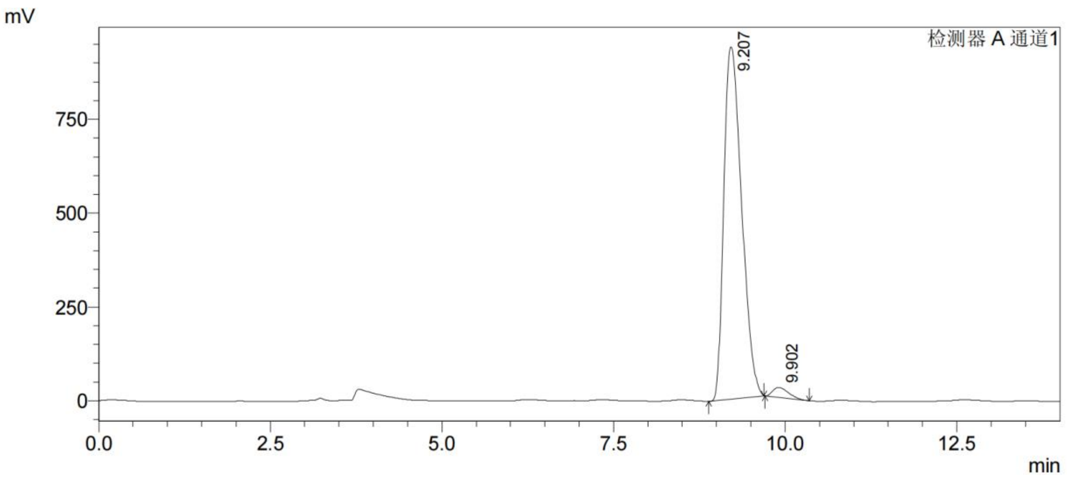

	Retention Time (min)	Relative Area (%)
Peak 1	9.207	97.538
Peak 2	9.902	2.462

Supplementary Fig. 55 HPLC spectra of compound of **2p**

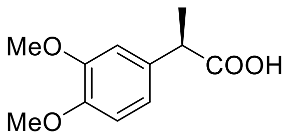

2q

95% ee. HPLC conditions: DAICEL Chiralpak OJ-H column, Hexane/*i*PrOH = 80/20, 210 nm, 1.0 mL/min, 25 °C, t_{major} = 12.119 min, t_{minor} = 14.275 min.

Racemate

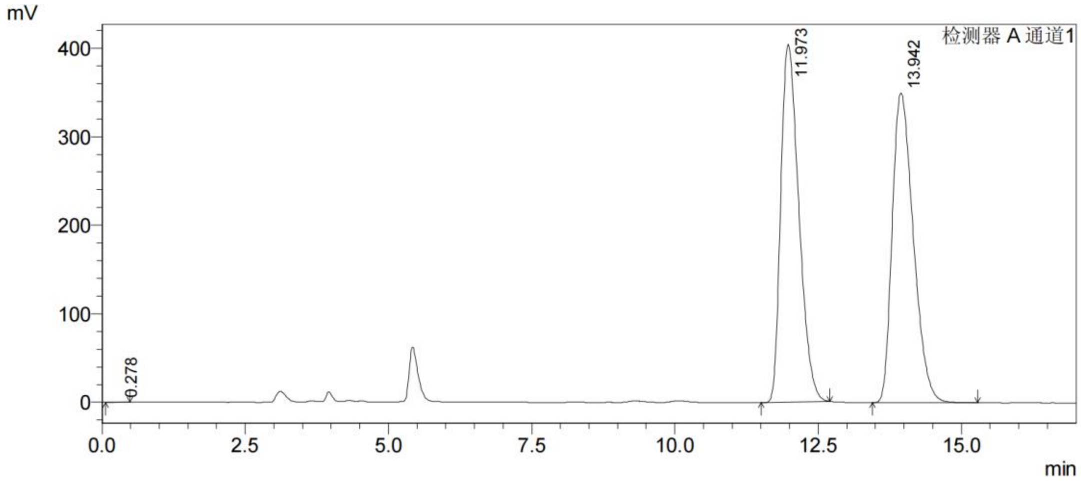

Chiral

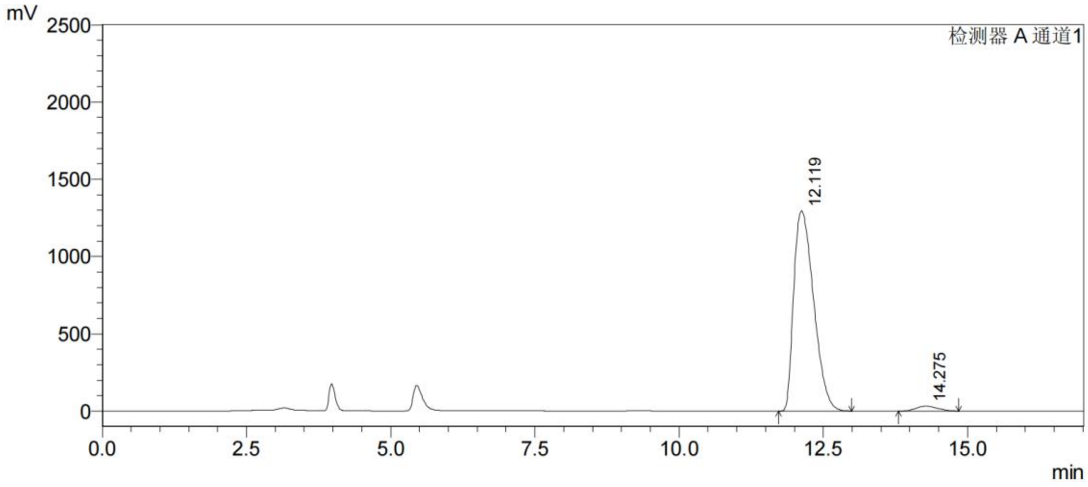

	Retention Time (min)	Relative Area (%)
Peak 1	12.119	97.414
Peak 2	14.275	2.586

Supplementary Fig. 56 HPLC spectra of compound of **2q**

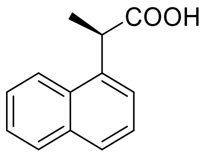

2r

99% ee. HPLC conditions: DAICEL Chiralpak OJ-H column, Hexane/*i*PrOH = 99/1, 210 nm, 1.0 mL/min, 25 ^{o}C, t_{minor} = 17.880 min, t_{major} = 20.106 min.

Racemate

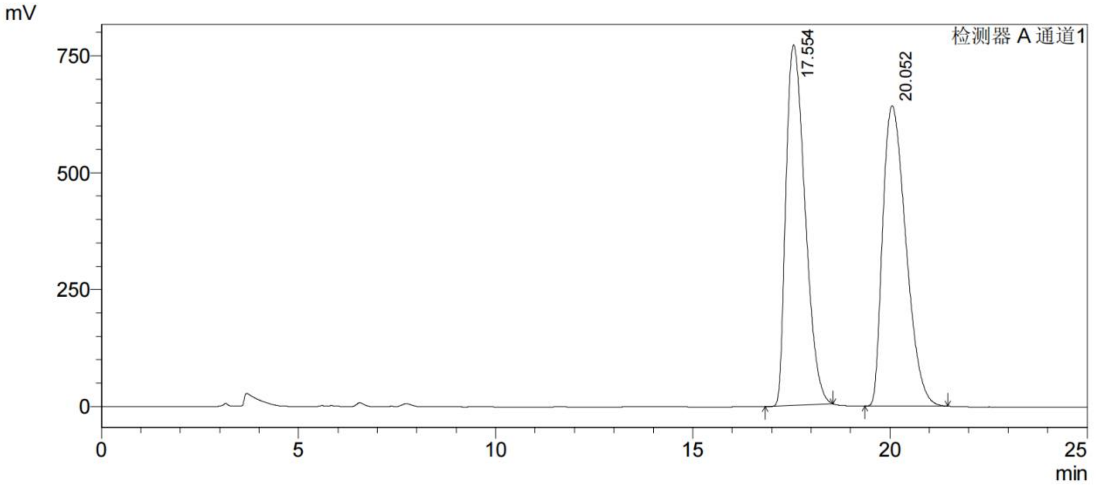

Chiral

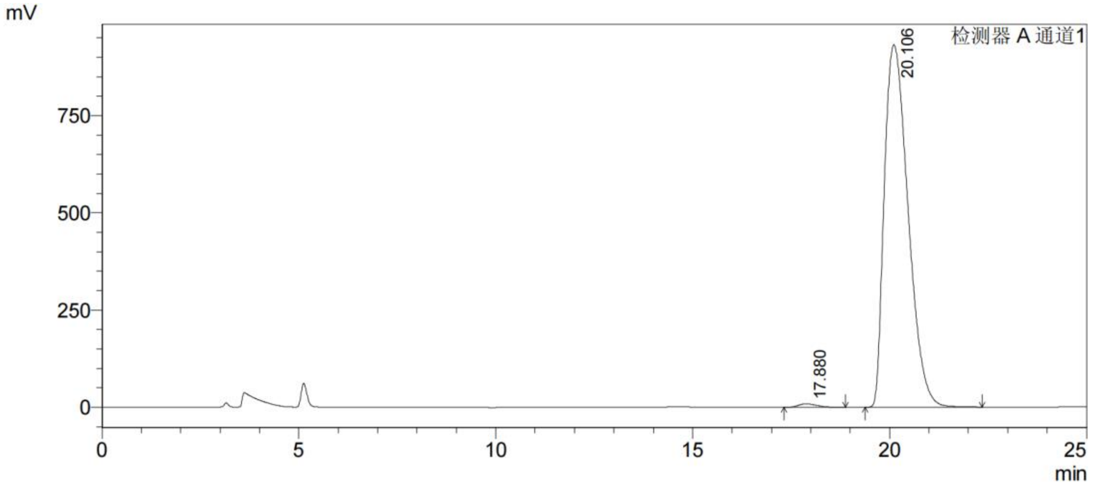

	Retention Time (min)	Relative Area (%)
Peak 1	17.880	0.726
Peak 2	20.106	99.274

Supplementary Fig. 57 HPLC spectra of compound of **2r**

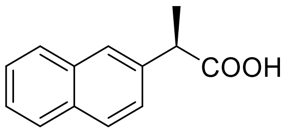

2s

95% ee. HPLC conditions: DAICEL Chiralpak OJ-H column, Hexane/*i*PrOH = 99/1, 210 nm, 1.0 mL/min, 25 ^{o}C, t_{minor} = 23.405 min, t_{major} = 25.048 min.

Racemate

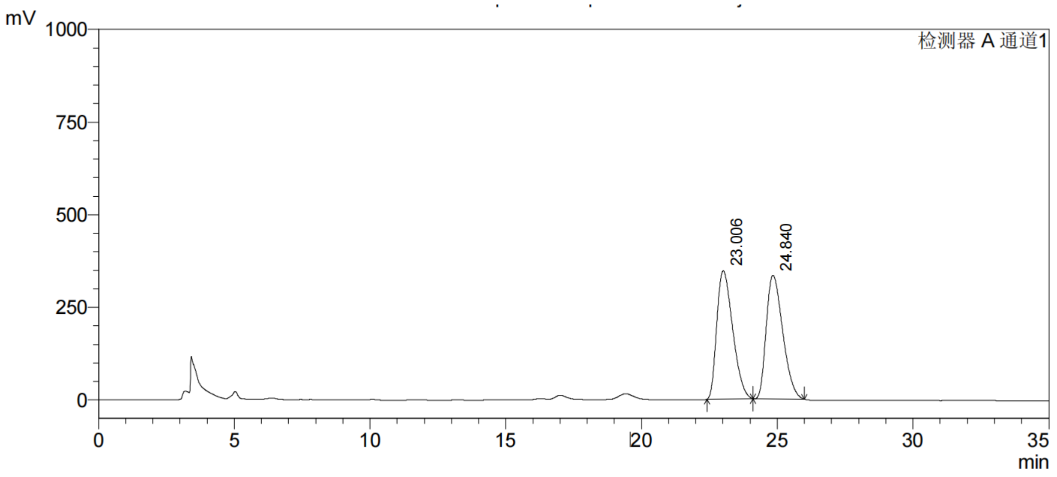

Chiral

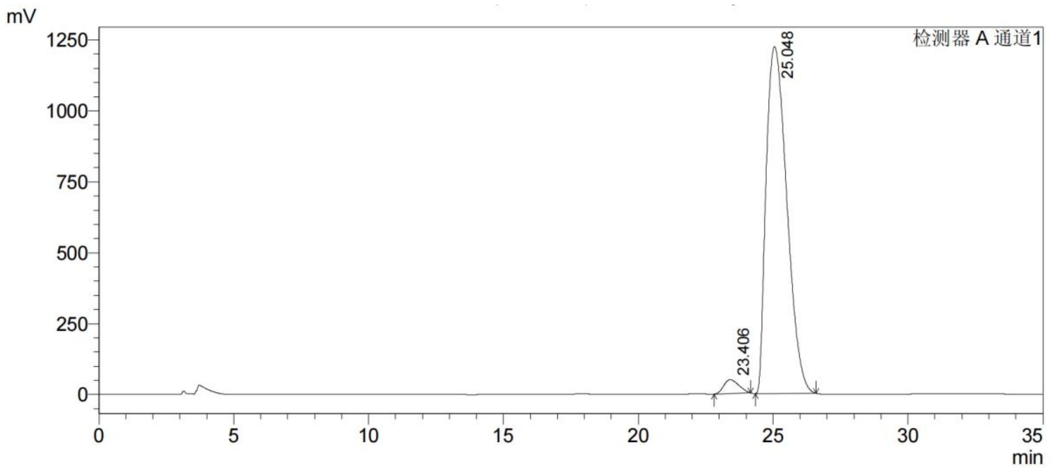

	Retention Time (min)	Relative Area (%)
Peak 1	23.406	2.817
Peak 2	25.048	97.813

Supplementary Fig. 58 HPLC spectra of compound of **2s**

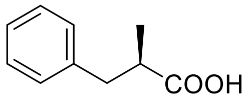

2t

90% ee. HPLC conditions: DAICEL Chiralpak OJ-H column, Hexane/*i*PrOH = 99/1, 210 nm, 1.0 mL/min, 25 ^{o}C, t_{major} = 9.093 min, t_{minor} = 10.159 min.

Racemate

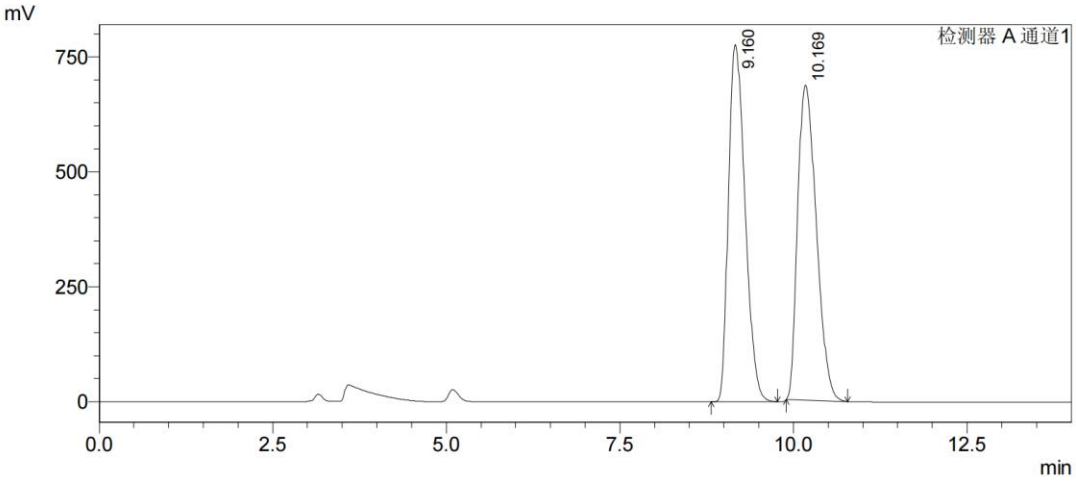

Chiral

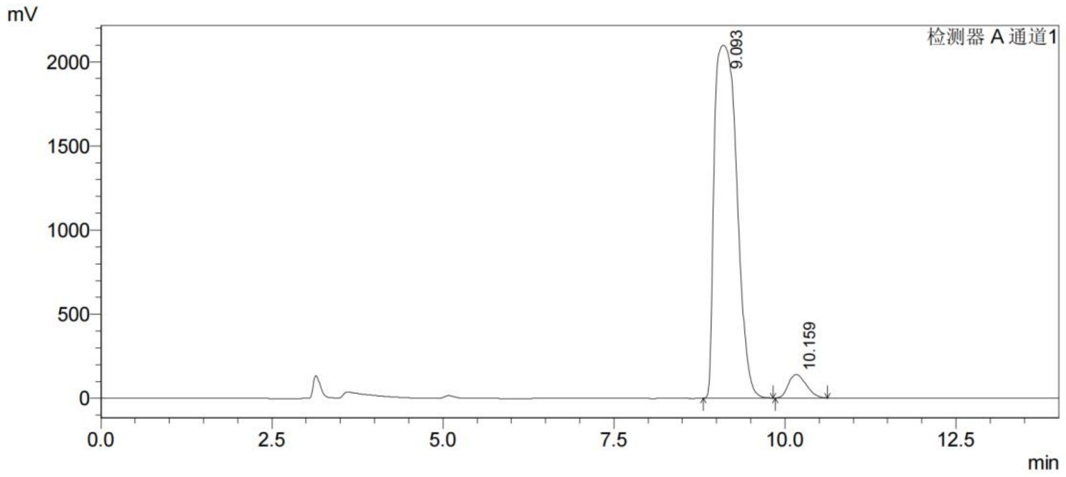

	Retention Time (min)	Relative Area (%)
Peak 1	9.093	94.959
Peak 2	10.159	5.041

Supplementary Fig. 59 HPLC spectra of compound of **2t**

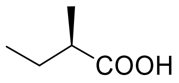

2u

94% ee. HPLC conditions: DAICEL Chiralpak AD-H column, Hexane/*i*PrOH = 98/2, 256 nm, 1.0 mL/min, 25 ^{o}C, t_{minor} = 32.759 min, t_{major} = 35.005 min.

Racemate

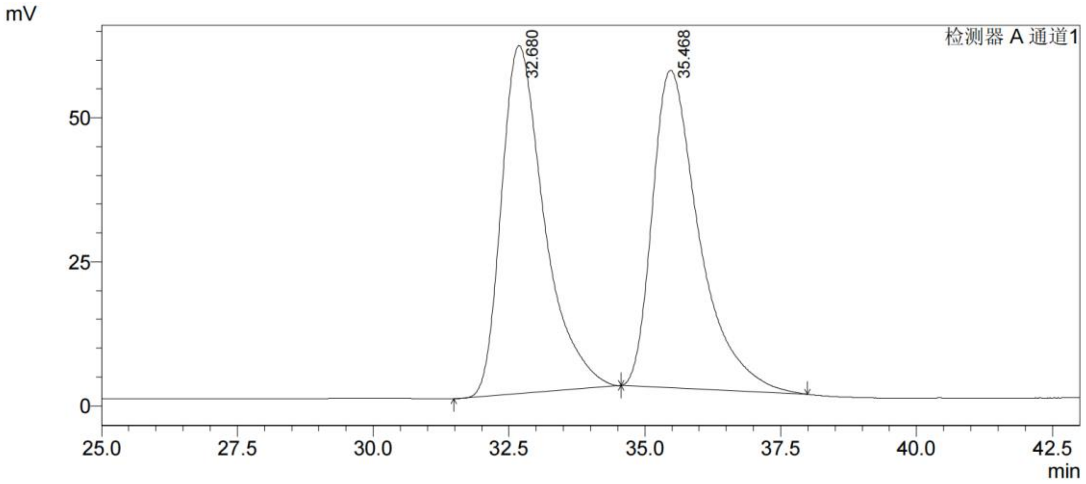

Chiral

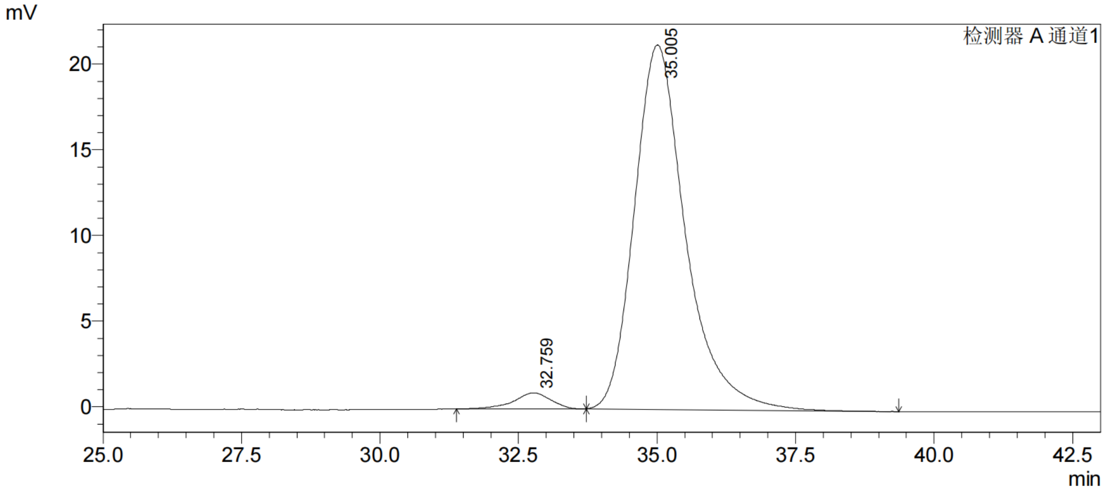

	Retention Time (min)	Relative Area (%)
Peak 1	32.759	3.085
Peak 2	35.505	96.915

Supplementary Fig. 60 HPLC spectra of compound of **2u**

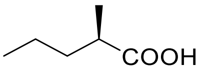

2v

94% ee. HPLC conditions: DAICEL Chiralpak AD-H column, Hexane/*i*PrOH = 95/5, 256 nm, 1.0 mL/min, 25 ºC, t_{minor} = 11.763 min, t_{major} = 12.984 min.

Racemate

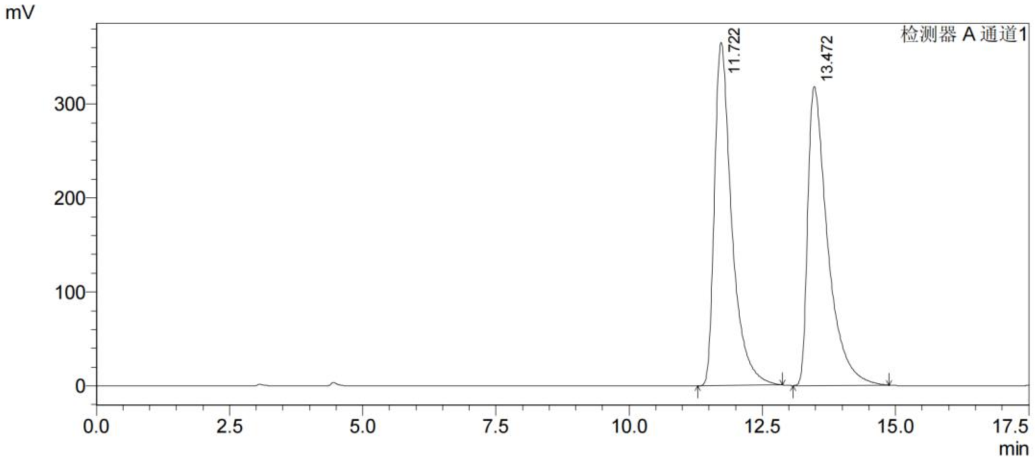

Chiral

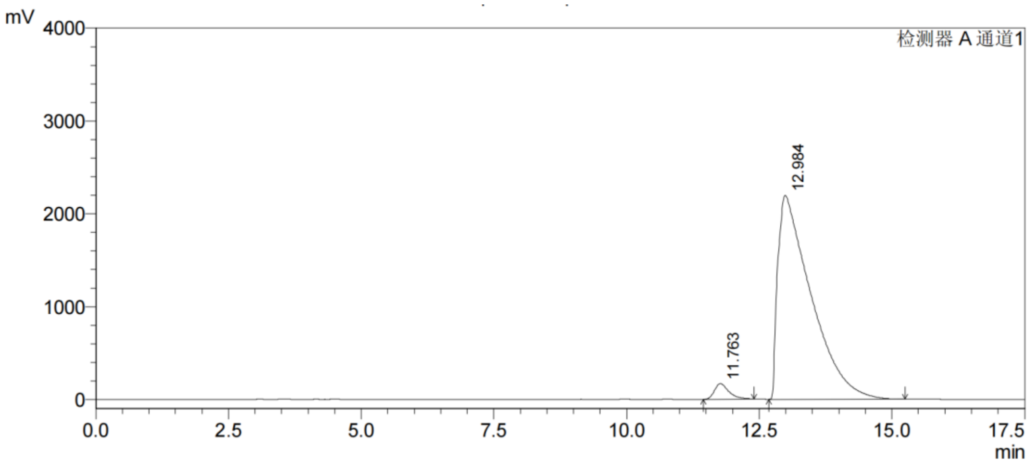

	Retention Time (min)	Relative Area (%)
Peak 1	11.763	3.189
Peak 2	12.984	96.811

Supplementary Fig. 61 HPLC spectra of compound of **2v**

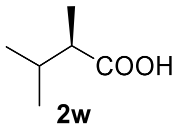

2w

91% ee. HPLC conditions: DAICEL Chiralpak AD-H column, Hexane/*i*PrOH = 98/2, 256 nm, 1.0 mL/min, 25 ^{o}C, t_{minor} = 28.509 min, t_{major} = 36.631 min.

Racemate

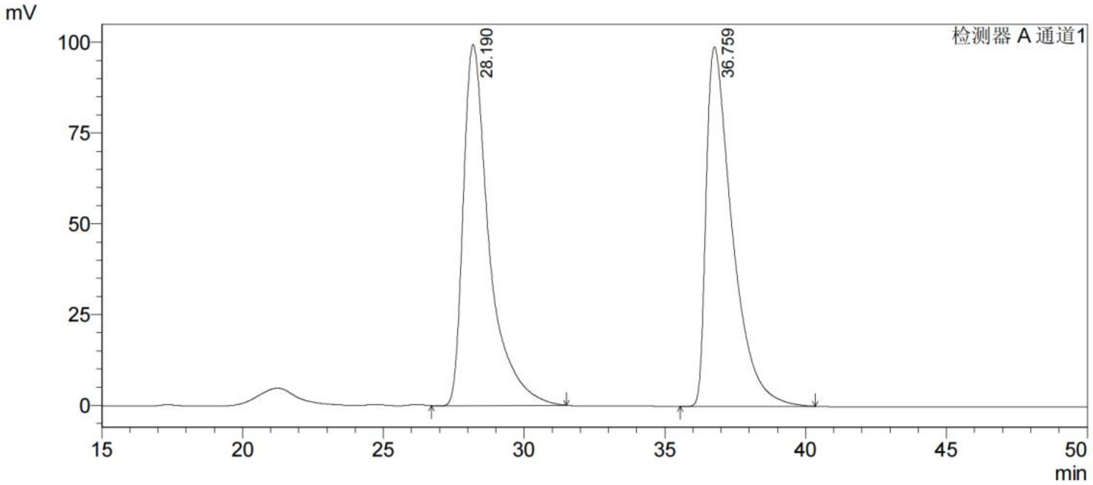

Chiral

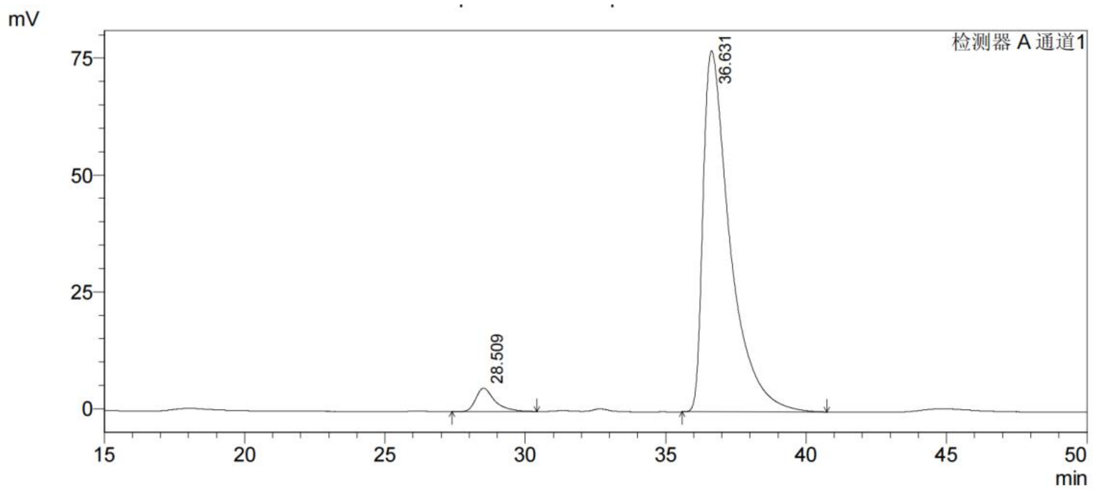

	Retention Time (min)	Relative Area (%)
Peak 1	28.509	4.342
Peak 2	36.631	95.658

Supplementary Fig. 62 HPLC spectra of compound of **2w**

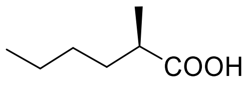

2x

95% ee. HPLC conditions: DAICEL Chiralpak AD-H column, Hexane/*i*PrOH = 98/2, 256 nm, 1.0 mL/min, 25 ^{o}C, t_{minor} = 29.363 min, t_{major} = 33.076 min.

Racemate

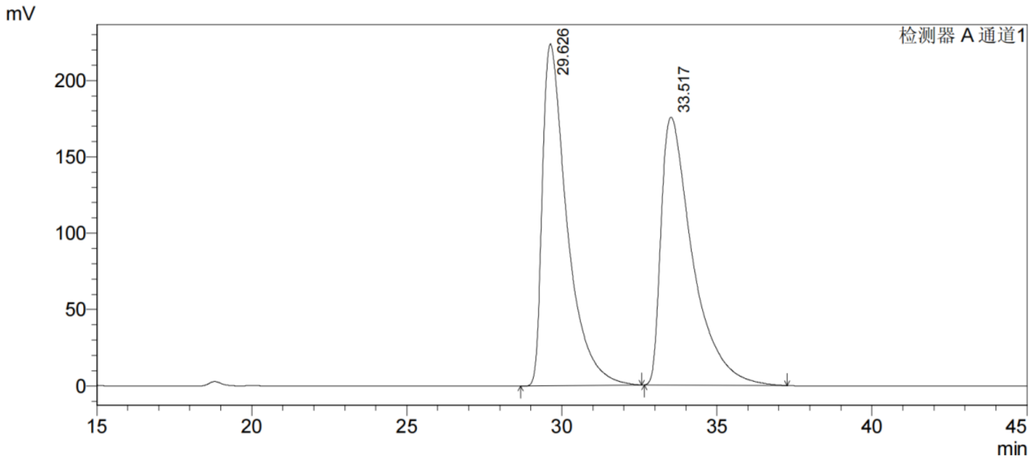

Chiral

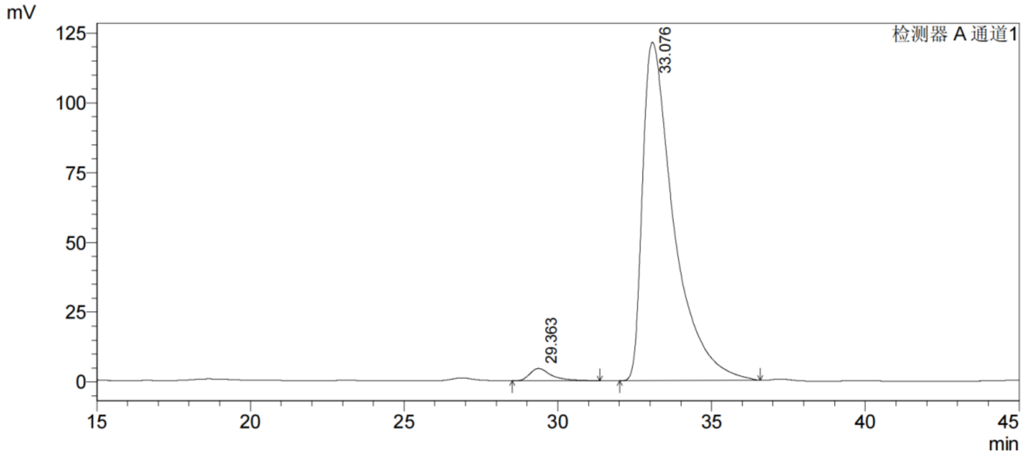

	Retention Time (min)	Relative Area (%)
Peak 1	29.363	2.511
Peak 2	33.076	97.489

Supplementary Fig. 63 HPLC spectra of compound of **2x**

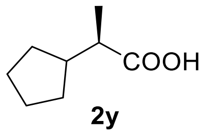

2y

91% ee. HPLC conditions: DAICEL Chiralpak AD-H column, Hexane/*i*PrOH = 98/2, 256 nm, 1.0 mL/min, 25 ^{o}C, t_{minor} = 38.279 min, t_{major} = 49.647 min.

Racemate

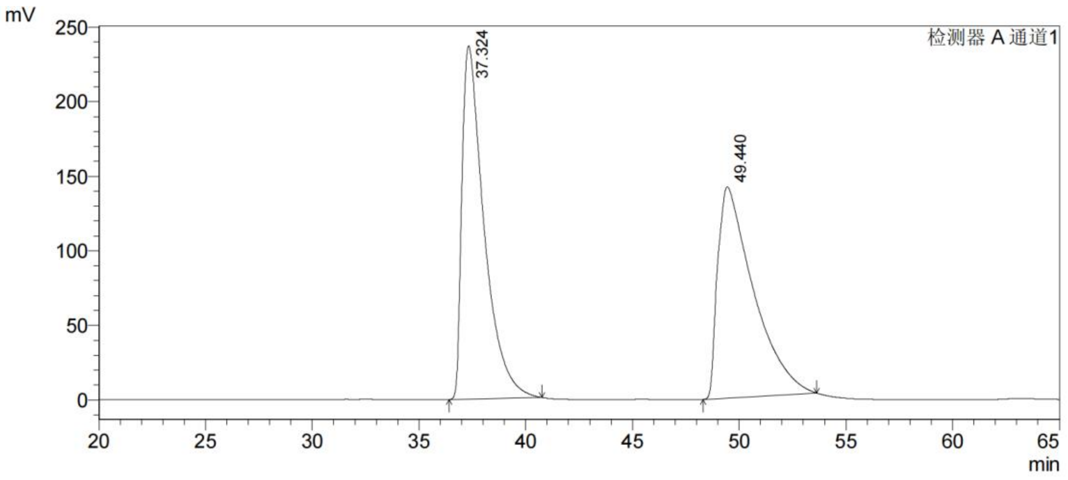

Chiral

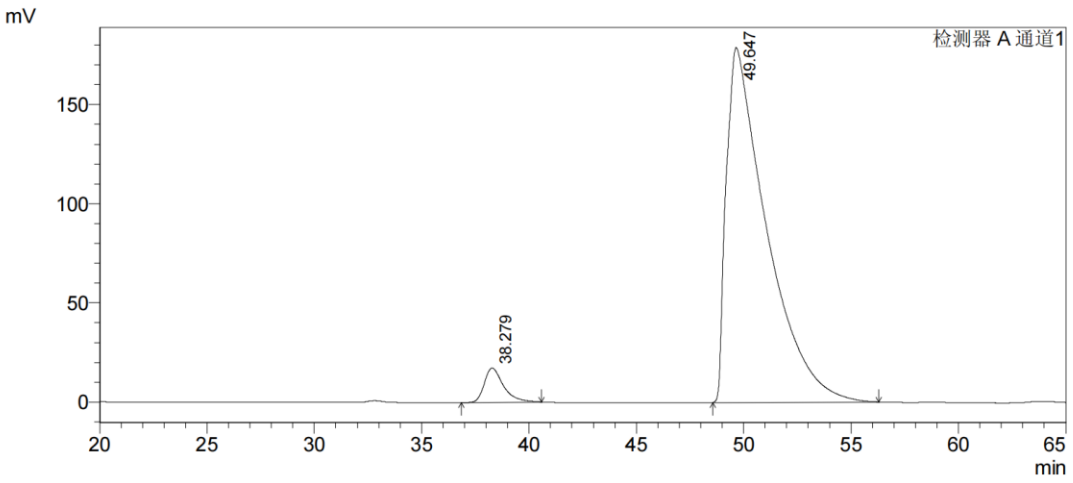

	Retention Time (min)	Relative Area (%)
Peak 1	38.279	4.401
Peak 2	49.647	95.599

Supplementary Fig. 64 HPLC spectra of compound of **2y**

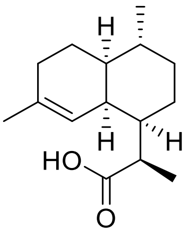

2z

99.8:0.2 dr. HPLC conditions: Agilent Eclipse XDB-18 column, aqueous H_3PO_4 (0.1%)/MeCN = 50/50, 210 nm, 1.0 mL/min, 25 ^oC, t_{major} = 28.674 min, t_{minor} = 29.907 min. Sample using $NaBH_4$ as a nonchiral reductant[9]

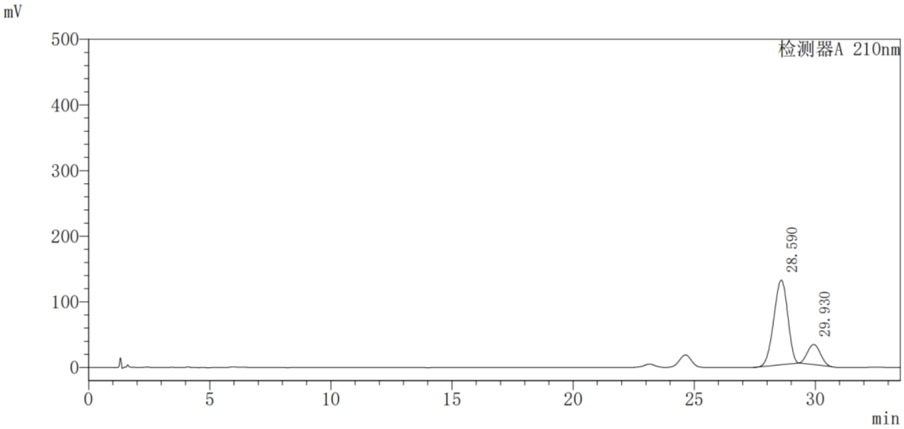

	Retention Time (min)	Relative Area (%)
Peak 1	28.590	81.834
Peak 2	29.930	18.166

Chiral

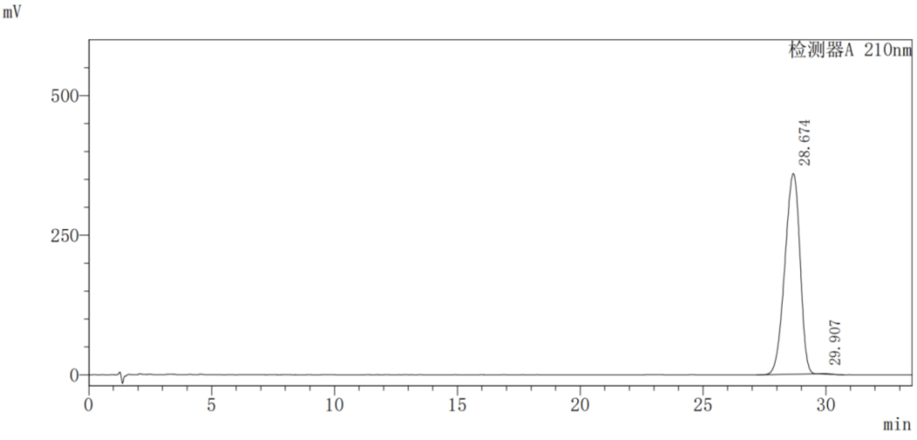

	Retention Time (min)	Relative Area (%)
Peak 1	28.674	99.855
Peak 2	29.907	0.145

Supplementary Fig. 65 HPLC spectra of compound of **2z**

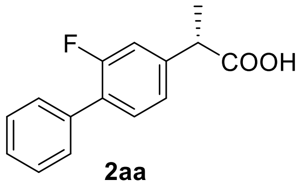

2aa

95% ee. HPLC conditions: DAICEL Chiralpak OJ-H column, Hexane/*i*PrOH = 99/1, 210 nm, 1.0 mL/min, 25 ^{o}C, t_{minor} = 17.878 min, t_{major} = 21.941 min.

Racemate

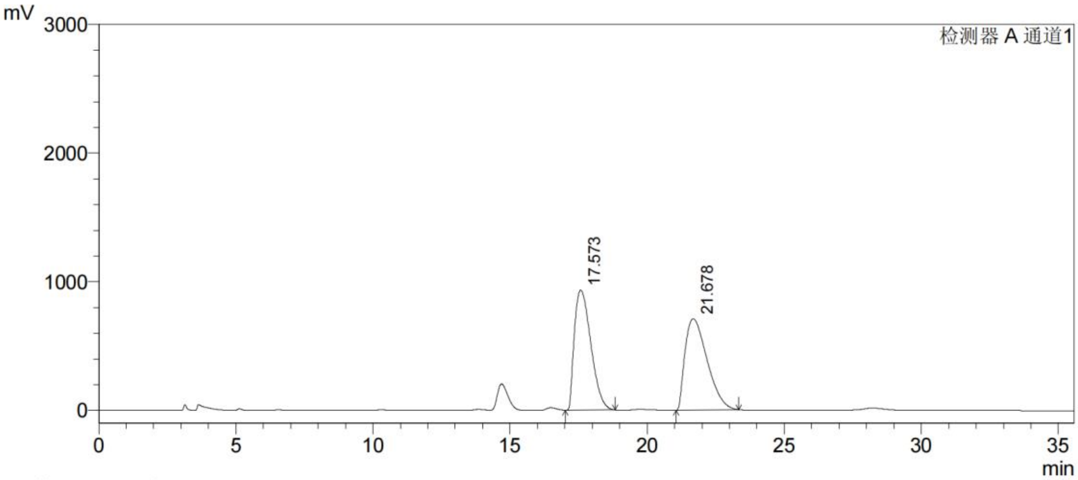

Chiral

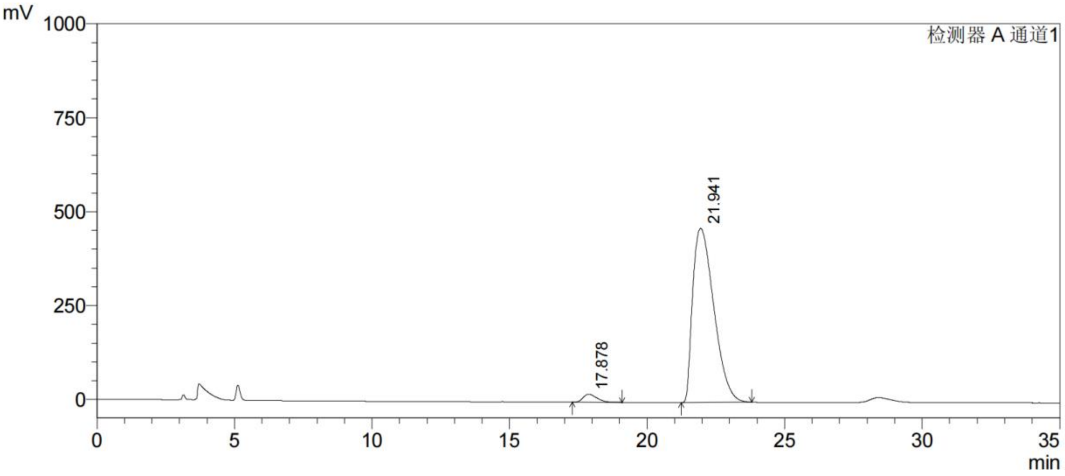

	Retention Time (min)	Relative Area (%)
Peak 1	17.878	2.917
Peak 2	21.941	97.803

Supplementary Fig. 66 HPLC spectra of compound of **2aa**

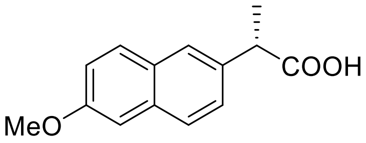

2ab

95% ee. HPLC conditions: DAICEL Chiralpak AD-H column, Hexane/*i*PrOH = 99/1, 210 nm, 1.0 mL/min, 25 ^{o}C, t_{major} = 11.616 min, t_{minor} = 12.751 min.

Racemate

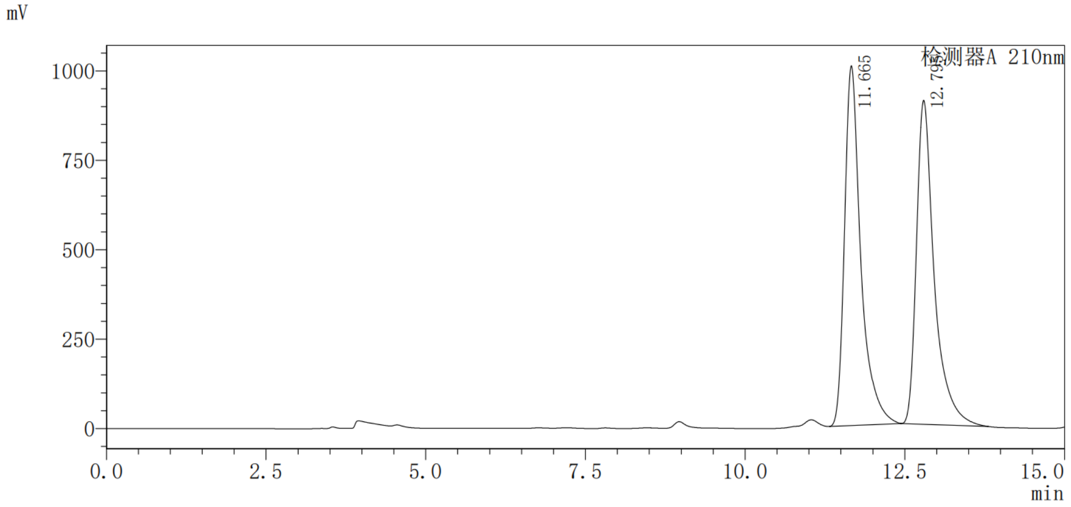

Chiral

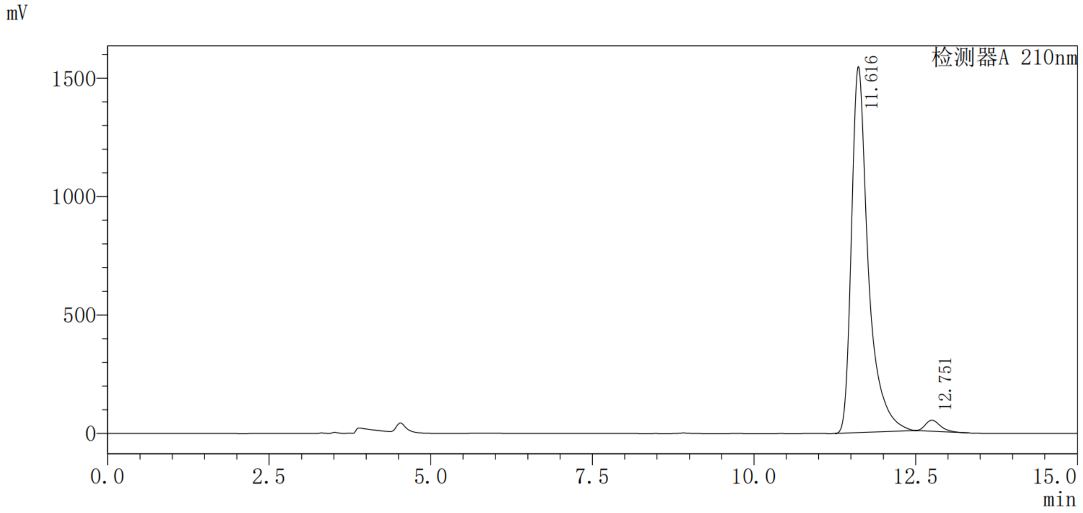

	Retention Time (min)	Relative Area (%)
Peak 1	11.616	97.355
Peak 2	12.751	2.645

Supplementary Fig. 67 HPLC spectra of compound of **2ab**

Supplementary References

1. Yang, Q.-L. *et al.* Electrochemistry-Enabled Ir-Catalyzed Vinylic C-H Functionalization. *J. Am. Chem. Soc.* **141**, 18970-18976 (2019).
2. Rana, N. K. & Singh, V. K. Enantioselective Enolate Protonation in Sulfa-Michael Addition to α-Substituted *N*-acryloyloxazolidin-2-ones with Bifunctional Organocatalyst. *Org. Lett.* **13**, 6520-6523 (2011).
3. Du, X. *et al.* Cobalt-catalyzed Highly Enantioselective Hydrogenation of α,β-Unsaturated Carboxylic Acids. *Nat. Commun.* **11**, 3239 (2020).
4. Yu, S., Hong, C., Liu, Z. & Zhang, Y. Synthesis of Cyclopentenones through Rhodium-Catalyzed C-H Annulation of Acrylic Acids with Formaldehyde and Malonates. *Org. Lett.* **23**, 5054-5059 (2021).
5. Basha, K. S. & Balamurugan, R. Gold(I)-Catalyzed Regioselective Hydroarylation of Propiolic Acid with Arylboronic Acids. *Org. Lett.* **25**, 4803-4807 (2023).
6. Hou, J., Xie, J.-H. & Zhou, Q.-L. Palladium-catalyzed Hydrocarboxylation of Alkynes with Formic Acid. *Angew. Chem. Int. Ed.* **54**, 6302-6305 (2015).
7. Kurtz, R. R. & Houser, D. J. A 1,6-Eliminative Epoxide Cleavage in the Synthesis of an Ibuprofen Metabolite. *J. Org. Chem.* **46**, 202-203 (2002).
8. Zhong, H., Shevlin, M. & Chirik, P. J. Cobalt-Catalyzed Asymmetric Hydrogenation of α,β-Unsaturated Carboxylic Acids by Homolytic H_2 Cleavage. *J. Am. Chem. Soc.* **142**, 5272-5281 (2020).
9. Li, J. *et al.* Asymmetric Hydrogenation of α-Substituted Acrylic Acids Catalyzed by a Ruthenocenyl Phosphino-oxazoline-Ruthenium Complex. *Org. Lett.* **18**, 2122-2125 (2016).
10. Shahid Islam, M., Ahmad, S., Attu, M. R., Foerstering, F. H. & Mahmun Hossain, M. Concise Synthesis of 2-Arylpropanoic Acids and Study of Unprecedented Reduction of 3-Hydroxy-2-arylpropenoic Acid Ethyl Ester to 2-Arylpropenoic Acid Ethyl Ester by BH_3·THF. *Helv. Chim. Acta* **98**, 1273-1286 (2015).
11. Smith, C. R. & RajanBabu, T. V. Catalytic Asymmetric Synthesis Using Feedstocks: an Enantioselective Route to 2-Arylpropionic Acids and 1-Arylethyl Amines via Hydrovinylation of Vinyl Arenes. *J. Org. Chem.* **74**, 3066-3072 (2009).
12. Tintori, C. *et al.* Development and in Vitro Evaluation of a Microbicide Gel Formulation for a Novel Non-Nucleoside Reverse Transcriptase Inhibitor Belonging to the *N*-Dihydroalkyloxybenzyloxopyrimidines (*N*-DABOs) Family. *J. Med. Chem.* **59**, 2747-2759 (2016).
13. Reed, J. H. & Cramer, N. 1,3,2-Diazaphospholenes Catalyze the Conjugate Reduction of Substituted Acrylic Acids. *ChemCatChem* **12**, 4262-4266 (2020).
14. Liu, W. *et al.* A Ligand-Directed Catalytic Regioselective Hydrocarboxylation of Aryl Olefins with Pd and Formic Acid. *Org. Lett.* **19**, 1748-1751 (2017).

15. Yu, J., Zhu, H., Zhang, X. & Chen, G.-Q. Development of C_2-Symmetric Chiral Diphosphine Ligands for Highly Enantioselective Hydrogenation Assisted by Ion Pairing. *Org. Lett.* **24**, 2744-2749 (2022).
16. Krimmer, S. G. *et al.* Rational Design of Thermodynamic and Kinetic Binding Profiles by Optimizing Surface Water Networks Coating Protein-Bound Ligands. *J. Med. Chem.* **59**, 10530-10548 (2016).
17. Clayman, P. D. & Hyster, T. K. Photoenzymatic Generation of Unstabilized Alkyl Radicals: An Asymmetric Reductive Cyclization. *J. Am. Chem. Soc.* **142**, 15673-15677 (2020).
18. Adamo, C. & Barone, V. Toward reliable density functional methods without adjustable parameters: The PBE0 model. *J. Chem. Phys.* **110**, 6158-6170 (1999).
19. Grimme, S., Ehrlich, S. & Goerigk, L. Effect of the damping function in dispersion corrected density functional theory. *J. Comput. Chem.* **32**, 1456-1465 (2011).
20. Grimme, S., Antony, J., Ehrlich, S. & Krieg, H. A consistent and accurate ab initio parametrization of density functional dispersion correction (DFT-D) for the 94 elements H-Pu. *J. Chem. Phys.* **132**, 154104 (2010).
21. Weigend, F. & Ahlrichs, R. Balanced basis sets of split valence, triple zeta valence and quadruple zeta valence quality for H to Rn: Design and assessment of accuracy. *Phys. Chem. Chem. Phys.* **7**, 3297-3305 (2005).
22. Li, X. & Frisch, M. J. Energy-Represented Direct Inversion in the Iterative Subspace within a Hybrid Geometry Optimization Method. *J. Chem. Theory Comput.* **2**, 835-839 (2006).
23. Fukui, K. The path of chemical reactions—the IRC approach. *Acc. Chem. Res.* **14**, 363-368 (1981).
24. Kesharwani, M. K., Brauer, B. & Martin, J. M. Frequency and zero-point vibrational energy scale factors for double-hybrid density functionals (and other selected methods): can anharmonic force fields be avoided? *J. Phys. Chem. A* **119**, 1701-1714 (2015).
25. Grimme, S. Supramolecular binding thermodynamics by dispersion-corrected density functional theory. *Chem. Eur. J.* **18**, 9955-9964 (2012).
26. Lu, T. & Chen, Q. Shermo: A general code for calculating molecular thermochemistry properties. *Comput. Theor. Chem.* **1200,** 113249 (2021).
27. Marenich, A. V., Cramer, C. J. & Truhlar, D. G. Universal solvation model based on solute electron density and on a continuum model of the solvent defined by the bulk dielectric constant and atomic surface tensions. *J. Phys. Chem. B* **113**, 6378-6396 (2009).
28. Lu, T. & Chen, F. Multiwfn: a multifunctional wavefunction analyzer. *J.* Comput*. Chem.* **33**, 580-592 (2012).
29. Lu, T. & Chen, Q. Independent gradient model based on Hirshfeld partition: A new method for visual study of interactions in chemical systems. *J. Comput. Chem.* **43**, 539-555 (2022).
